# ADGRE5-centered Tsurv model in T cells recognizes responders to neoadjuvant cancer immunotherapy

**DOI:** 10.3389/fimmu.2024.1304183

**Published:** 2024-01-26

**Authors:** Jian Li, Zhouwenli Meng, Zhengqi Cao, Wenqing Lu, Yi Yang, Ziming Li, Shun Lu

**Affiliations:** Shanghai Lung Cancer Center, Shanghai Chest Hospital, Shanghai Jiaotong University, School of Medicine, Shanghai, China

**Keywords:** non-small cell lung cancer, neo-adjuvant immunotherapy therapy, tumor microenvironment, ScRNA-seq, multimodal

## Abstract

**Background:**

Neoadjuvant immunotherapy with anti-programmed death-1 (neo-antiPD1) has revolutionized perioperative methods for improvement of overall survival (OS), while approaches for major pathologic response patients’ (MPR) recognition along with methods for overcoming non-MPR resistance are still in urgent need.

**Methods:**

We utilized and integrated publicly-available immune checkpoint inhibitors regimens (ICIs) single-cell (sc) data as the discovery datasets, and innovatively developed a cell-communication analysis pipeline, along with a VIPER-based-SCENIC process, to thoroughly dissect MPR-responding subsets. Besides, we further employed our own non-small cell lung cancer (NSCLC) ICIs cohort’s sc data for validation in-silico. Afterward, we resorted to ICIs-resistant murine models developed by us with multimodal investigation, including bulk-RNA-sequencing, Chip-sequencing and high-dimensional cytometry by time of flight (CYTOF) to consolidate our findings *in-vivo*. To comprehensively explore mechanisms, we adopted 3D ex-vivo hydrogel models for analysis. Furthermore, we constructed an ADGRE5-centered Tsurv model from our discovery dataset by machine learning (ML) algorithms for a wide range of tumor types (NSCLC, melanoma, urothelial cancer, etc.) and verified it in peripheral blood mononuclear cells (PBMCs) sc datasets.

**Results:**

Through a meta-analysis of multimodal sequential sc sequencing data from pre-ICIs and post-ICIs, we identified an MPR-expanding T cells meta-cluster (MPR-E) in the tumor microenvironment (TME), characterized by a stem-like CD8^+^ T cluster (survT) with STAT5-ADGRE5 axis enhancement compared to non-MPR or pre-ICIs TME. Through multi-omics analysis of murine TME, we further confirmed the existence of survT with silenced function and immune checkpoints (ICs) in MPR-E. After verification of the STAT5-ADGRE5 axis of survT in independent ICIs cohorts, an ADGRE5-centered Tsurv model was then developed through ML for identification of MPR patients pre-ICIs and post-ICIs, both in TME and PBMCs, which was further verified in pan-cancer immunotherapy cohorts. Mechanistically, we unveiled ICIs stimulated ADGRE5 upregulation in a STAT5-IL32 dependent manner in a 3D ex-vivo system (3D-HYGTIC) developed by us previously, which marked Tsurv with better survival flexibility, enhanced stemness and potential cytotoxicity within TME.

**Conclusion:**

Our research provides insights into mechanisms underlying MPR in neo-antiPD1 and a well-performed model for the identification of non-MPR.

## 1 Introduction

Sustainable responses to multiple advanced carcinomas acquired through ICI-based combination therapies have paved the way toward neo-ICI downstaging for the enlargement of resectable cancers. Atezolizumab (atezo), nivolumab (nivo) (1), pembrolizumab (pembro) (2) and durvalumab (durvo) (3) have all achieved satisfactory MPR rates as monotherapies (4) or in combination with chemotherapy (chemo) (5) and other immunotherapies (6). The NEOSTAR trial (1) even demonstrated MPR rates of up to 50% (11/22) in the nivo plus ipilimumab (ipi) and CT arms compared to 32.1% (7/22) in the nivo plus chemotherapy arm. Meanwhile, immunosuppressive macrophages can potentially lead to resistance to neoadjuvant chemo+ICIs in recurrent glioblastoma (7). A multicellular community organized by cDC2 and specific cancer-associated fibroblast (CAF) subtypes was also associated with Non in neoadjuvant ICIs in pancreatic ductal adenocarcinoma (PDAC) (8).

Accordingly, distinguishing the MPR from the Non is of clinical significance, but heterogeneity across the TME in different tumor types under various treatment regimens has hindered current investigations considerably, among which the diversity of CD8^+^ tumor-infiltrating lymphocytes (TILs) might still be the biggest obstacle. Adrienne et al. demonstrated that tissue-resident memory CD8^+^ T cells (CD8^+^ Trm cells) serve as clonally expanded TILs (T-E) in response to nivo monotherapy or ipi+nivo combination (2 cycles) in head and neck squamous cell carcinomas (HNSCC) (9), with hallmark genes ITGAE (CD103) and HOBIT (ZNF683), as well as ICs such as LAG3, CD223 and TIGIT. In addition, a signature derived from CD8^+^ Trm cells predicts a favorable prognosis in HNSCC patients receiving ICI therapy, the molecular spectrum of which resembles that of T-E in breast cancer (BC) patients treated with a 1-cycle pembro (10). In contrast, precursor-exhausted CD8^+^ TILs (Tpex) with high GZMK expression and diminished ICs accumulated within the responsive (R) TME after chemo+nivo in non-small cell lung cancer (NSCLC) (11), while the nonresponsive (NR) TME was filled with exhausted Trm cells instead. Although Caushi et al. investigated tumor antigen-specific CD8^+^ TILs in MPRs with resectable early-stage NSCLC after 2 cycles of nivo monotherapy and confirmed a similar Tpex pattern (12), another study focused on metastatic NSCLC under chemo+nivo claimed that T-E was dominated by Tex (13). Such inconsistency could be attributed to the delicate balance between Tpex and Tex (14), which are intertwined with different metastatic stages (15), treatment regimens (1), biopsy sampling timepoints (16) and intrinsic tumor-type features (13).

From another perspective, heterogeneity among CD8^+^ TILs responding to ICIs is merely the tip of the iceberg. Correspondingly, explorations of non-T-cell populations, such as IgG^+^ plasma B cells (17), CXCL12^+^ CAFs (18) and tumor stem cells (CSCs) (19), have generated various signatures with the potential to predict the ICI response. However, the sensitivity and accuracy of such models vary across different tumor types and omics datasets.

Herein, to simplify the research background and control variables mentioned above, we started with the integration of longitudinal single-cell transcriptomics sequencing (sc) of NSCLC CD3^+^ TILs from patients who underwent 2 cycles of nivo monotherapy and further leveraged large-scale corroboration profiling in pan-cancer ICI-sc and bulk RNA sequencing (bulk RNA-seq) datasets. The MPR-E characterized by survT was found to be enriched in the MPR cohort compared to the Non or pre, which was the same as what was observed in the murine TME by cytometry by time of flight (CYTOF) and bulk-RNA-seq analysis. We identified adhesion G protein-coupled receptor E5 (ADGRE5) as a key feature of survT in the MPR by using a cell communication analysis pipeline that we developed ourselves. Based on the SCENIC algorithm and Chip-seq analysis, a STAT5-regulated, IL32-dependent ADGRE5 pathway was established and verified in our own independent NSCLC neo-PD1 cohorts. Afterwards, we generated an ADGRE5-centered Tsurv model with good performance in distinguishing the MPR from Non among multiple tumor types, pre-ICIs and post-ICIs. Taking advantage of the 3D hydrogel-based tumor-immune cell coculture system (3D-HYGTIC) constructed by us (20), we demonstrated that the PD-1-triggered increase in ADGRE5 expression was dependent upon IL32, which was suppressed by a STAT5 inhibitor. Finally, concomitant delivery of anti-PD-1 and ADGRE5^+^survT rescued the resistance to ICIs to some extent, indicating the potential ability of ADGRE5^+^survT in clinical settings.

## 2 Materials and methods

### 2.1 Datasets

Two NSCLC scRNA-seq cohorts were adopted as discovery sets (GSE179994 (11) and GSE173351 (12)). All other data, including PIC-seq (**Supplementary Figures S5, 7**), validation set for VIPER-pro, Chip-seq dataset, and datasets for the Tsurv model (section 3.7), are available in **Supplementary Table S1**, **S4**.

### 2.2 Data pre-processing and integration

Seurat R package (v3.2.3) (21) was firstly utilized for pre-processing of collected datasets as aforementioned, including filtering, integration, normalization, and Louvain clustering. The filtering of genes was restricted to mitochondrial genes. Afterward, “NormalizeData” and “ScaleData” were performed. Then, “FindVariableFeatures” (method of “vst”) was conducted to select 3,000 highly-variable genes (HVG). HVG went through principal component analysis (PCA) to identify the top 50 principal components (PCs). To eliminate batch effects, the calculated PCA matrix was fed into the ‘RunHarmony’ function (Harmony (v1.0) (22)) in Seurat using default parameters with patient ID as the batch key for ten iterations. After Harmony integration, UMAP visualization and the Shared Nearest Neighbor (SNN) graph construction were made using PCs 1 to 40 and k=25 nearest neighbors. Then the Louvain clustering algorithm was used to cluster cells (function of “FindClusters” with resolution=0.94). Fixed parameters mentioned above were chosen after the iteration of a list of an arithmetic progression of parameters to ensure optimized distinct compartments of sub-clusters.

### 2.3 Machine-based manual annotation of cell sub-clusters

scPred combined unbiased feature selection from a reduced-dimension space with machine learning probability-based prediction (23). We first built a cell classifier from a dataset with prior cell type annotation from Zhang et al. Then, we trained it using the scPred method. Next, we classified cell types using the scPredict function. SCINA (24) is an algorithm that can automatically detect and assign cell types. We used prior knowledge of cell type signatures (25) and set the sensitivity cutoff to 0.9 while disallowing unknown cell types. According to these, we first determined the general distribution pattern of naive, memory, effector, exhausted, proliferated, and regulated cells within CD4 and CD8; then, a more detailed manual annotation was performed based on the correlation calculated by the SingleR function from SingleR, and we identified subclusters with reference to the TIL atlas proposed by Zhang et al.

### 2.4 Genes enrichment analysis

#### 2.4.1 DEGs

scCODE (single-cell Consensus Optimization of Differentially Expressed gene detection) (26), is an R package to automatically optimize DE gene detection for each experimental scRNA-seq dataset proposed by Xin Zou et al. We use two exclusion criteria to obtain immune-related gene sets (scCODE-irFilter):

1) The gene symbols contain “KRT”, “TMEM”, “HIST”, “TUB”, “ANK”, “APB”, “BAI”, “BAR”, “BEX”, “BMP”, “orf”, “FAM”, “LINC”, “MMP”, “MMR”, “RBM”, “SNA”, “ZNF”, “ZBT”;
2) Biological Process (GO) annotation from Metascape website contains “actin”, “nuclea”, “nucleu”, “histone”, “spermine”, “transport”, “voltage”, “cation”, “development”, “transmitter”, “kinetochore”, “wound”, “spermidine”, “tubulin”, “biological_process”, “synapse”, “micro”, “DNA”, “RNA”, “neuron”, “axon”, “motor”, “muscle”, “filament”, “contraction”, “skeletal”, “nerve”, “collagen”, “vessel”, “rhombomere”, “endothelial”, “embryo”, “hemidesmosome”, “meiotic”.

Genes differentially expressed in MPR vs Non for cells in MPR-E were identified using the scCODE package (log2FC>0.2, FDR<0.05, adjusted by the Benjamini-Hochberg method) and were filtered with the criteria above, as well as DEGs for Viper.

To identify genes that were differentially expressed among Pre, Non-MPR, and MPR in mouse CD8T cells, we used the limma, EdgeR, and Deseq2 methods (log2FC>0.5, FDR<0.05, adjusted by the Benjamini-Hochberg method). We selected the intersection of the DEGs identified by all three methods. These DEGs were then subjected to GO analysis using the clusterProfiler (27) tool. For constructing a transcriptional regulatory network, we used the up-regulated DEGs in MPR compared to Non as input into the string website and visualized the network using the Cytoscape platform.

### 2.5 Pathway analysis

The pathway activities of C7 and C8 gene sets from MSigDB were evaluated by irGSEA, a tool having integrated all single cell rank-based gene set enrichment analysis with “UCell” methods, as well as the gene sets in section 3.1.

Using the DEGs mentioned above, we executed GO (Gene Ontology), KEGG (Kyoto Encyclopedia of Genes and Genomes), and Reactome enrichment analyses using the ‘clusterProfiler’ R package in section 3.1 and 3.4. We conducted functional annotation analysis based upon biological processes (BP), molecular function (MF), and cellular component (CC). Benjamini-Hochberg method was used to make adjustments to the p-value, with a p-value <0.05 defined as statistically significant.

To perform gene set enrichment analysis (GSEA), we first ranked the gene list by log2FC and input it into the pre-ranked GSEA function in the ‘clusterProfiler’ R package. We used the DEGs between MPR and Non (section 3.4) and up-regulated markers from CD8.c05/11/12 (**Supplementary Figure S1, 2F**) as the gene set.

### 2.6 Monocle3

We learned trajectory graphs in CD4 and CD8 TIL separately with Monocle3 (28), while inheriting cluster discrimination from Seurat aforementioned, giving each individual cell a pseudotime value, which represents the distance (relative time) from the original root defined manually with reference to literature and function of “get_earliest_principal_node”. Signatures scoring along the order of cells was calculated by irGSEA introduced above (**Supplementary Table S3**) (29). Scores were computed and visualized by Complexheatmap (30).

### 2.7 Cell communication analysis pipeline

#### 2.7.1 CellChat

CellChat R package version 1.4.0 (31) was used for L-Rs investigation within 20 subpopulations identified previously, with the ‘CellChatDB.human’ L-Rs interaction database as reference data. Separate CellChat objects for pre, Non, and MPR were conducted, later with comparison analysis to infer differentially enriched L-Rs interactions using the ‘compareInteractions’ function. Dominant sender and receiver cells in 2D space were portraited by the ‘netAnalysis_signalingRole_scatter’ function. The strength of L-Rs interactions was compared using ‘netVisual_heatmap’ function, and visualized using the ‘netAnalysis_signalingRole_heatmap’ function, with options set to “all”, “incoming”, and “outgoing”. To directly compare L-Rs interactions we used the ‘RankNet’ function. To visualize interactions among subpopulations, we used ‘netVisual_aggregate’ function with the ‘layout = “circle”‘ option. Finally, for the specific pathway, we used the ‘netAnalysis_signalingRole_network’ function to show interaction patterns in different groups.

To perform CellChat on VIPER, we calculated the minimum value of each gene in the normalized matrix from VIPER, added the absolute minimum value to the entire matrix, except for zero values, and used this as the input data for CellChat.

#### 2.7.2 CellphoneDB

To investigate L-Rs among clusters from each meta-cluster, we analyzed L–Rs using CellphoneDB (v.3.1.0) and database (v4.0.0) (32). L-Rs appeared within over 10% of cells of certain clusters were extracted for illustration. We focused on L-Rs having consistent performances in Cellchat and NicheNet, then compared differences between log (mean (Ligand)) and log (mean (Receptor)) in MPR versus Non.

### 2.8 NicheNet

To focus on intercellular interactions, we performed the Differential NicheNet (33), which is an extension of the default NicheNet, to improve the performance of resolutions for comparing between different niches and better predicting niche-specific L-Rs pairs. We used DEGs (pre-ICIs versus post-ICIs) as targets to figure out what ligands from CD4 T cells in MPR-E and PD1-R potentially influenced CD8.c05/11/12. We showed the prioritization scores of the top 50 ligands (as for their highest scoring receptor) (section 3.2). in the post niche (score averaged over the 3 analyses), and of all the postcondition L-Rs pairs with a prioritization score ≥ the score of the pre. DEGs of cd8.c05/11/12 between post and pre were selected as our interested gene sets filtered by lfc_cutoff = 0.15, expression_pct = 0.1 and pvalue < 0.05 as commended.

### 2.9 SCENIC

Based on the single-cell RNA-seq results, we used the SCENIC or pySCENIC (34) to infer the regulatory network of TFs based upon old version databases (https://resources.aertslab.org/cistarget/databases/old/). Each regulatory network was considered a regulon. By analyzing the regulon activity in each cell type, we identified differences in the regulatory activity of TFs among sub-clusters, and then used GENIE3 to target filtered genes with significance. Finally, Rcistarget was adopted for determining regulons based on the StarGet dataset, with AUCell to quantify the activity of regulons.

### 2.10 VIPER

The regulatory network in this study was reverse-engineered using the ARACNe-AP algorithm (35). We generated networks for each patient’s immune cells from each cluster and integrated the networks. The relative activity of each protein represented in the network was inferred using the VIPER algorithm v1.26.0 (36), similar to the master regulator inference algorithm (MARINA) (37), which uses the MR targets inferred by the ARACNe algorithm. In addition to calculating the enrichment of ARACNe-predicted targets in signatures, statistical significance for VIPER-pro filtering, including p-value and normalized enrichment score (NES), was estimated by comparison to a null model generated by permuting the samples uniformly at random 1,000 times.

### 2.11 Chip-seq analysis

NCBI site(ftp://ftp.ncbi.nlm.nih.gov/biosample) was used for downloading datasets (**Supplementary Table S4**. database) to construct a Chip-seq database. Sequencing data in every SRX were organized into the format of fastq (method of “fastq-dump” in SRA Toolkit (ver.2.3.0) by default settings. Produced fastq data were aligned afterward using Bowtie2 (38) (ver.2.2.2) with default parameters and then integrated to make our Chip-seq database. Reads underwent alignments to hg38 (H. sapiens) or mm9 (M. musculus). Genome regions (BED) of ADGRE5 and random permutation of ADGRE5 were counted with the “intersect” function within BEDTools2 (39) (ver.2.23.0). Two-tailed Fisher’s test was adopted for P-value calculation between them (null hypothesis meaning same proportion at peak-call of the two datasets). peak-caller MACS2 (40) (v2.2.7) was used to determine thresholds for statistical significance values. Significantly enriched TFs responsible for ADGRE5 regulation were depicted (**Supplementary Table S4**. ADGRE5).

### 2.12 Proteomics analysis

The raw mass spectrometric files were proceeded by MaxQuant computational platform (41) (version 2.4.0.0) using sequencing data from Uniprot-Swiss-prot database (FDR < 1% for either peptides or proteins). Potential contaminants were then filtered, as well as for reverse hits and those that were conclusively identified by site. Log2 scale transformation was conducted for LFQ values, then pooling of three technical replicates and the average was taken, and proteins were filtered for at least three valid values in any of the experimental groups that existed. For missing values, imputation was performed by a normal distribution (width =0.4; shift 1.6), based upon the assumption of setting expression of missing proteins adjacent to the detection limit. We then used the ANOVA function (Perseus) with FDR <0.05 and S0 of0.4 to find out feature proteins that marked prominent differences among various CD8^+^ T cells sub-clusters. Finally, a Protein ruler in Perseus was adopted to calculate protein copy numbers per cell through standardization to histone MS signaling on the whole.

### 2.13 Construction of Tsurv model

We developed a signature discovery module (section 3.7) to identify signatures capable of ICIs therapy response identification and ICIs prognosis prediction.

To obtain genes that can best predict the ICB therapy response status, we first collected genes up-regulated in MPR in all analysis pipelines: scCODE, Monocle3, Cell-communication pipeline, VIPER and SCENIC. Taking advantage of the PIC dataset (**Supplementary Table S1**. PIC_bulk_RNA_seq), we then adopt batch operation of ROC analyses for PR/PD classification and OS prediction analyses for each gene separately and simultaneously, and only kept those with significance (P-value<0.05). To be noticed, we separated PIC datasets according to cancer types, and then integrated sequencing data as well as patients’ prognosis information in a cancer-type-specific manner. For example, with all melanoma-derived data integrated, we then further dissected it into training sets and validation sets, while all the other cancer types were handled as validation datasets. Meanwhile, for sc-sequencing datasets (**Supplementary Table S1**. single_cell), we utilized them as independent validation datasets separately without integration.

To construct the Tsurv signature, feature selection algorithms (particle swarm algorithm (PSA) and recursive feature elimination (RFE)) were applied. In addition, machine learning algorithms (logistic regression (LR), random forest (RF) and support vector machine (SVM)) were adopted for label classification. We traversed all permutations of feature selection algorithms and classification algorithms aforementioned to determine the optimal combination. RFE combined with LR was selected because of their optimal performance in cross-validations (4-, 6-, 8-, 10-fold).

### 2.14 CYTOF

Murine TIL were sorted by MACS (Miltenyi Biotec, Cat: 130-110-618) as has been suggested in bio-protocol. Cells were restimulated with 50ngml^−1^ phorbol 12-myristate 13-acetate (Sigma–Aldrich) and 500ngml^−1^ ionomycin (Sigma–Aldrich) in the presence of 1× Brefeldin A (BD Biosciences) for 4h at 37°C. 3×10^6^ Cells per condition (pre, Non, MPR) were stained with 100μL of 250nM cisplatin (Fluidigm) for 5min on ice, and then incubated in Fc receptor blocking solution before surface antibodies staining (30 mins on ice). Cells were then fixed in 200μL of intercalation solution (Maxpar Fix and Perm Buffer containing 250nM 191/193Ir, Fluidigm) overnight. After fixation, cells were stained with intracellular antibodies cocktail for 30 min on ice. After adding 20% EQ beads (Fluidigm), cells were acquired on a mass cytometer (Helios, Fluidigm).

Antibodies were acquired from eBioscience, Biolegend, R&D systems and BD Biosciences (**Supplementary Table S2**). Labeling of antibodies by indicated metal tag was performed through the MaxPAR antibody Labelling kit (Fluidigm). The optimal concentration was decided, then a doublet-filtering scheme was adopted for debarcoding from raw data. Afterward, bead normalization method was used to control batch effects, and debris, doublets and dead cells were removed to get single living cells. CATALYST R package (1.24.0) was used for NRS calculation, FlowSOM-based cell population identification, as well as umap-based dimensional reduction visualization.

### 2.15 qRT-PCR

CD8^+^ T cells in TILs were sorted by beads (StemCell). Trizol (Thermo Fisher) was used to extract RNA (2*10^6 CD8^+^ T cells). PrimeScript RT reagent Kit (Takara Bio) was adopted for reverse transcription reactions. Afterward, TB Green Premix Ex Taq II (Takara Bio) was adopted for quantifying genes from cDNA. Amplification of Genes (primers in **Supplementary Table S6**) was conducted by >40 cycles at 95C for 15 s, and then 60C for 15 s to finally 72C for 45 s. Normalization of genes to Gadph was conducted and then comparisons among different groups (MPR, Non, pre) were performed.

### 2.16 Cell culture

The Lewis lung carcinoma cell line (LLC) was used for the murine tumor model and 3D HYGTIC model. LLC was purchased from the Shanghai Institute for Biological Sciences Chinese Academy of Sciences (Shanghai, China). Complete medium for LLC was prepared as: Dulbecco’s modified eagle medium (DMEM) with high glucose (Hyclone, Cat: SH30027.02) with heat-inactivated 10% fetal bovine serum (FBS, Gemini, Cat: SH3015103) and 1% penicillin/streptomycin (P/S, GIBCO, Cat No 15140-122) at 37C (5% CO2 atmosphere).

### 2.17 Anti-PD-1 resistant murine models

LLC were injected subcutaneously (s.c.) into 6 to 8-week-old mice (3*10^5 LLC for each). Volumes of tumors were measured starting from day 7 and were calculated by length x width^2)/2. Anti-mouse CD279 (RMP1-14, BioXcell) (200 mg/100 μL) were injected from day 6 every day intraperitoneal (i.p.), with tumor volume monitored. Mice in the control group were treated with 200mg/100 μL isotype IgG2 (i.p.). For ACT therapy, mice in the MPR group (n:5) (determined by monitoring tumor volume through two paired t-tests) were sacrificed. Tumor specimens were digested and handled by Percoll gradient centrifugation to get TIL. KLRD1^neg^ ADGRE5^+^ CD8^+^ TIL was sorted (survT) (BD Fortessa AriaIII, BD Biosciences), with the other cells collected as control group cells, and survT and control group cells were cryopreserved till use. Antibodies for sorting were in **Supplementary Table S2** sheet 2. From day16 to day21, survT (to Non-survT group mice, n=3) or control group cells (to Non group mice, n=3) were injected intratumorally together with anti-mouse CD279 as aforementioned, with mice sacrificed at day21 (tumor volume <2000mm^3^).

### 2.18 bulk-RNA-seq analysis

CD8^+^ T cells in TIL were sorted by MACS beads (StemCell). The total RNA of purified CD8^+^ T cells was extracted with Trizol (ThermoFisher). RNA quality was assessed on an Agilent 2100 Bioanalyzer (Agilent Technologies). Eukaryotic mRNA was enriched by Oligo(dT) beads. Enriched mRNA was reversely transcribed into cDNA by NEBNext Ultra RNA Library Prep Kit for Illumina (Cat: 7530). After end-repairing, A-base-adding and ligating of cDNA fragments to adapters, PCR-amplified cDNA was sequenced with Illumina Novaseq6000 (Gene Denovo Biotechnology Co.). Afterward, Fastqc was used for quality control, and GRCm39 from Ensemble release 107 using STAR (v.2.5.2b) was adopted for reads mapping. We first computed DEG between MPR and Non with DEseq2, EdgeR and limma and conducted an intersection to get DEG_up and DEG_down. DEGs along the T cells differentiation trajectory from Monaco et al. (https://www.proteinatlas.org/) were recorded. We grouped them into Tn-Teff or Tex due to their relative expression (scaling to 0-1 by “minmax” algorithm among clusters) (HPA). At the same time, we assessed their prognostic value in PIC (**Supplementary Table S1**) and divided them into bad prognosis, not significant and good prognosis groups based on their HR by Cox proportional hazards regression models and P value by log-rank test (PIC). Then we used the DEG_up for GSEA analysis across pre and MPR or Non. Afterward, we built the PPI signature by developing the PPI networks with the intersection genes from DEG_up, PIC, HPA, and ADGRE5-CD55 axis on the STRING website (https://string-db.org/).

### 2.19 Flow cytometry and sorting

#### 2.19.1 Flow cytometry

Mouse TILs or splenocytes, as well as human TIL and PBMC, were stained with Zombie Violet (Biolegend) viability dye solution (1:1000) with specific antibodies, incubated at 4°C for 38 min, washed and fixed before intracellular staining. Stimulation for intracellular staining was conducted as in CYTOF aforementioned by Cyto-Fast™ Fix/Perm Buffer Set (Biolegend, Cat: 426803). For intra-nuclear TFs staining, True-Nuclear™ Transcription Factor Buffer Set (Biolegend, Cat: 424401) was used. Antibodies in flow cytometry as well as for sorting were listed in **Supplementary Table S2**. BD LSR Fortessa X20 (Beckton Dickson) was the facility for the collection of stained cells. Fcs data were processed through FlowJo (10.0.1).

#### 2.19.2 Sorting

CD45^+^ TIL or CD8^+^ TIL for CYTOF, bulk-RNA-seq, flow cytometry or 3D HYGTIC experiments were sorted by MACS beads (Stem cell) or by flow cytometry sorting, after confirming its purity by flow cytometric analysis. Cells (diluted in FACS) density was counted by hemacytometer manually. ≥ 10x10^6^ cells were centrifuged, and then resuspended with specific antibodies (**Supplementary Table S2**) and incubated at RT for 15 minutes in dark for sorting. Using BD FACS Aria III (BD Biosciences), ≥1x10^6^ cells for each condition would be sorted. Sorted cells were washed with 0.25% BSA and counted.

### 2.20 Immune cells proliferation assay

Lyophilized CFSE dye (Biolegend, cat: 423801) was prepared to 5mM stocking solution, then a 1:1,000 dilution ratio was adopted for working solution immediately before use. Cells (1*10^7 cells/ml) sorted above-mentioned or dissociated from 3D HYGTIC were centrifuged (2,000 rpm), resuspended, and incubated in CFSE dyeing solution further at room temperature in dark for 20 min. After quenching with FACS 5 times, cells were collected for further staining or loaded for flow cytometry immediately.

### 2.21 Immunofluorescence

Fresh tumor tissues were fixed and dehydrated as has been proposed. For mIF, tumor sections from the following independent ICI cohort (2 patients for MPR, Non, and pre respectively) were collected. OCT embedding agent (Servicebio) was used to embed tissues before staining. Briefly, 3% BSA (diluted in PBS) was adopted for blocking non-specific binding, then anti-human ADGRE5 Ab (1:150) (Absin, Cat: abs132702) was incubated at 4°C overnight. PBS (pH 7.4) washing of slides 3 times was conducted. Slides were then incubated with Cy5-goat anti-rabbit (1:300) for 70 min in dark (room temperature). After washing by PBS, anti-human CD8α Ab (1:300, Absin, Co: abs158658) staining was performed. Then counterstain of the DAPI solution (1:4000) was performed. Leica Sp8 laser scanning confocal microscope and ImageJ software were used for imaging and image-data processing respectively.

### 2.22 Dissociation of human NSCLC tumor specimen

Single-cell suspensions from collected biopsies were obtained according to previously published literatures (16). Briefly, biopsies or surgery fragments (diagnosed by pathologists according to intra-operative frozen section) were processed by mechanical dissection thoroughly then in enzymatic cocktail at 37C in a shaker (45rpm). 2 patients for each group (MPR, Non, pre) were used for single cell dissociation, and patients’ tissues within the same group were gathered together to obtain enough cells for 3D HYGTIC and sc sequencing. Enzymatic cocktail was configured from human tumor dissociation kit (Miltenyi Biotec, Cat: 130-095-929). 100 mm strainer was first used to achieve cell bulks (patient-derived tumor fragments (PDTF), meaning dozens of cells gathered together) for the convenience of 3D HYGTIC construction. Then 40um strainer was used to obtain single-cell suspension. Percoll gradient (GE Healthcare) centrifuge and subsequent MACS were adopted for further enrichment of CD45^+^ TIL for downstream 5’ single-cell RNA-seq.

### 2.23 sc-RNA-sequencing for the independent ICI cohort

The sc-RNA sequencing was performed using Chromium Single Cell Reagent Kits (v3) (10x Genomics). CD45^+^ TIL in the former step was kept on ice for <4h, and library construction was performed within 6h from the extraction of the tumor specimen. 10x Chromium Next GEM Chip K was loaded with cells from the six samples (20,000 cells per sample, for every condition: MPR, Non, pre, there were 2 samples pooled together) respectively. The reverse transcription and cDNA amplification were performed. Qsep (BiOptic) and Qubit HS dsDNA kit (ThermoFisher) were adopted for measurement of size distribution and DNA concentration respectively. Expression libraries were constructed, and sequenced by Illumina Novaseq, obtaining >55,000 reads per cell for every expression library.

### 2.24 3D HYGTIC model construction

3D HYGTIC model was constructed as has been previously described (20). Briefly, PDTF from human tumor fragments, or tumor cells sorted from murine TME (from cell clumps below Percoll layer) by tumor isolation kit (Miltenyi Biotec, Cat: 130-110-187) (MDTF), were collected for 3D tumor-microspheres construction. 5μl GelMA-PEO mixture containing 40,000 cells/μl was pipetted to make microspheres in BIOFLOAT™ 96-well plate (faCellitate, Cat: F202003), and was cultured and grown till day 7 in organoid culture medium. TIL was separated and T cells were isolated as aforementioned. Isolated T cells were cultured in T cells expanding medium (TEM, which was prepared as follows: ImmunoCult-XF T Cell Expansion Medium (10981, Stemcell) supplemented with 1000 IU/mL Recombinant IL-2 (human: 200-02; mice: 212-12; PeproTech). 0.5×10^6^ expanded T cells after 2 days culturing in TEM were added to each 3D tumor-microsphere containing PDTF or MDTF and set up as 3D-HYGTIC, with 50ng/ml mIL-2 and no CD3/28 addition. For sorted TIL above-mentioned (**Supplementary Table S2** sheet2), 3D-HYGTIC was constructed without TIL expansion to exclude its interference on proliferation monitoring by Ki67 and CFSE.

## 3 Results

### 3.1 Meta-cluster MPR-E expansion for MPR images compared to non or pre-images

In the TME, which is a complex ecological biosystem, multiple cell types, including CD36^+^ CAFs (42), plasma cells (17), and cancer stem cells (CSCs) (19), interact with each other and influence ICIs prognosis (43). Since T cells are major targets of neo-antiPD1 (44), we used sorted T-cell scRNA-seq data from the NSCLC TME from Zhang et al. (11) and Caushi et al. (12). Considering that post-ICI specimens were obtained after 2 cycles of mono-nivolumab treatment, we were able to strictly exclude other covariations, such as tumor types (16), metastases (45, 46), ICI combination strategies (47), and ICI cycles, which were all correlated with heterogeneous changes in TILs. After filtering doublets and red blood cells, 164,754 CD3^+^ T cells were collected from 23 MPR samples, 34 non-MPR samples and 33 pre-ICI samples. To enable a reasonable comparison between MPRs (33,432 cells) and non-MPRs (117,284 cells), we sampled 45% of the non-MPRs.

**Figure 1:**
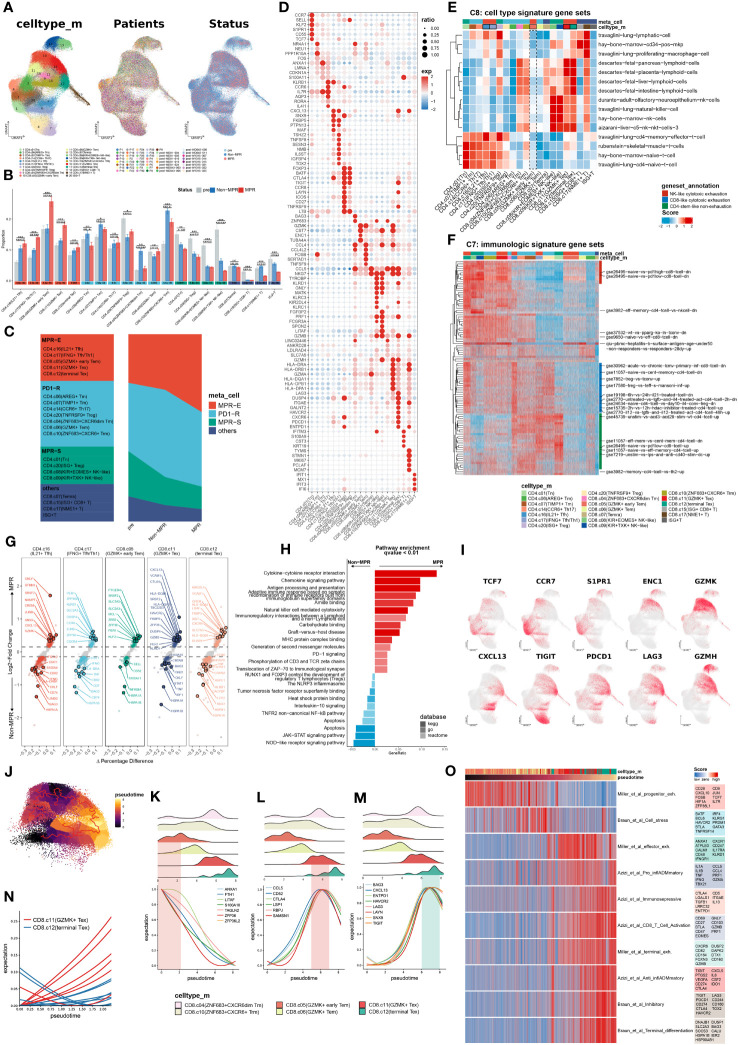
Meta-cluster MPR-E expanding in MPR compared to Non or pre. **(A)** Uniform manifold approximation and projection (UMAP) of cell clusters, patients ID and status from integrated scRNA-seq data of 164,754 sorted CD3^+^ T cells, which are further defined in **(F)**. **(B)** Comparison of the frequency of 20 clusters respectively in all CD4^+^ T cells and CD8^+^ T cells from pre, Non, MPR. Statistical testing between any pair of two group by two-sided t-test and Kruskal-Wallis’s rank sum test for three groups (bottom) (*p < 0.05, **p < 0.01, ***p < 0.001). **(C)** Definitions of the four meta-clusters for CD3^+^ T cells. MPR-E, MPR-expanding. PD1-R, PD1-responding. MPR-S, MPR-shrinking. **(D)** Heat map of selected MSigDB C8 gene sets scores in each cluster. **(E)** Heat map of selected MSigDB C7 gene sets scores in each cluster. **(F)** Dot plot of differentially expressed genes found in each cluster. **(G)** Volcano plot showing differentially expressed genes between MPR and Non from MPR-E clusters; each colored dot denoting an individual gene with adjusted P value < 0.05 (scCODE) and |log (Fold change)≥ 0.2. **(H)** Pathway enrichment analysis of DEGs from MPR-E meta cluster between MPR and Non in KEGG, GO and REACTOME database and pathways with q-value < 0.01 were shown here. **(I)** UMAP feature plots showing the expression levels of certain genes. **(J)** 60,939 CD8^+^ T cells colored by pseudotime inferred by Monocle3. **(K–N)**. The distribution of CD8^+^ T cells along with the pseudotime (upper panel) and fitting curve for the expression levels of certain signature genes along the pseudotime (lower panel). **(O)**. Heat map of CD8^+^ T cells related signatures scores per cell in CD8.c05/06/11/12 along the pseudo-time. Select genes in each genesets are shown.

With the guidance of the Scpred (23) and SCINA (24) clustering methods (**Supplementary Figure S1A**, **S1B**, methods), we manually identified 20 subtypes of CD3^+^ TILs (**Figures 1A, D**) with reference to the atlas of TILs (TIL atlas) proposed by Zhang et al. (48) Certain subtypes were well recognized due to obvious marker overexpression, such as FOXP3 in TNFRSF9^+^ Tregs, ISG15 in ISG^+^ Tregs, NKG7 in CD8.c08 (KIR^+^EOMES^+^NK-like), and MKI67 and NME1 in NME1^+^ CD8.c17 (NME^+^T) (**Supplementary Figure S1D**). Since exclusive marker detection was rather difficult for other subtypes, we performed correlation analysis with the TIL atlas (**Supplementary Figure S1H**) and successfully identified the other clusters, among which a transition from stem-like CD4.c01, (T naïve) marked by KLF2 and SELL, to exhausted CD4.c16 (IL21^+^Tfh) and CD4.c17, (IFNG^+^Tfh/Th1) marked by SNX9 and TOX2, was observed (**Figure 1D**, **Supplementary Figure S1C**), as was the transition of GZMK to GZMB expression from CD8.c05 (GZMK^+^ early Tem) to CD8.c12 (terminal Tex) (**Supplementary Figures S1E–G**, **S2A**).

We hypothesized that the MPR inherited certain subcluster expansions compared to those in the non-MPR (Non) and pre-ICI (pre) groups, thus revealing the cluster distribution in each group (**Figure 1B**). Indeed, CD4.c16, CD4.c17, CD8.c05, CD8.c11 and CD8.c12 experienced significant gradual expansions from pre to Non groups and then to MPR and were subsequently grouped into a meta-cluster named MPR-E. Those with the most obvious fluctuation in Non against pre, but that showed no change in the MPR, were indicated as the PD1-responding (PD1-R) meta-cluster, which included CD8.c10 (ZHF683^+^CXCR6^+^Trm) and CD4.c14 (CCR6^+^ Th17), and those within a downward trend were classified as the MPR shrinking meta-cluster (MPR-S), which included CD4.c01 (Tn) and CD8.c08 (KIR^+^EOMES^+^NK-like). Other subpopulations, such as CD8.c15 (ISG^+^CD8^+^T), were excluded from further investigation due to their limited sizes (**Figure 1C**).

To decipher the functional signatures of these meta-clusters, we performed functional annotation via irGSEA (method) on all MSigDB C7 and C8 6,049 pathways (49) (**Supplementary Figures S4A, B**). C8, as a sc-derived annotation database, was intended to elucidate cluster definition. CD4.c16 appeared to be a travaglini-lung-cd4-memory-effector-t-cell analog, and CD4.c17 was more likely to be a travaglini-lung-cd4-naïve-t-cell analog (**Figure 1E** and **Supplementary Figure S4A**). CD8.c11 and CD8.c12 cells exhibited fetal-like lymphoid profiles, which indicated their gradually increasing tolerance phenotypes (50–52), which was in accordance with the upregulation of TIGIT, PDCD1 and LAG3. On the other hand, CD8.c05 showed no obvious correlation with any cluster in C8. We further profiled the immunological pathways from C7 (**Figure 1F** and **Supplementary Figure S4B**). All other meta-clusters were associated with certain functional enrichment pathways. However, compared with those in CD8.c11 and CD8.c12, CD8.c05 in MPR-E merely manifested moderate gse26495-naïve-vs.-pd1-high-cd8-t-cell-dn enrichment, suggesting that its functional profile is rather obscure.

We then selected scCODE (26) to determine the MPR-E characteristics in an attempt to capture features of CD8.c05 cells. The MPR-E subgroup was enriched in cytokine- and chemokine-mediated cytotoxicity pathways, as well as cell−cell immunoregulatory interactions (**Figure 1H**). CD4.c16 and CD8.c11 both showed upregulation of CXCL13 in the MPR (**Figure 1G**), which was in line with the indispensable role of CXCL13 in identifying tumor-reactive CD8^+^ expanding T-cell clones in multiple cancer types (16, 53). Moreover, the expression of PER1 and KLRB1 in CD4.c17 cells, as well as that of HLA-DQA1 and KIR2DL4 in CD8.c12 cells (**Figure 1G**), indicated significantly enhanced cytotoxicity of CD4.c17 and CD8.c12. However, in addition to the widely accepted stemness-related markers CST7, CCR7 and TCF7, other cluster-specific markers of CD8.c05, such as SIPR1 and ENC1 (**Figure 1I**, **Supplementary Figure S2A**), as well as MPR-enriched DEGs such as PTGER4 and SRSF2 (**Figure 1G**), still lacked certain research related to their roles in tumor immunology, which made the features of CD8.c05 even more unclear.

To further clarify trajectories of specific clusters in MPR-E, we conducted a Monocle3 analysis. In accordance with our analysis above, from CD4.c16 to CD4.c17, the differentiation trajectory oriented toward CD4.c01 was accompanied by gradually enhanced expression of antigen-presenting molecules such as CD74, DUSP4 and HLA-A, along with decreased expression of SELL, IL7R and CCR7 (**Supplementary Figures S2B**, **S3A, B**). We further identified functional modules associated with the CD4^+^ T cells differential trajectory, among which modules 22 and 41 were the most upregulated in CD4.c16 and CD4.c17 cells, respectively (**Supplementary Figures S2C, D**). LAG3 CXCL13 and CXCR5, the chemokine receptor responsible for TIL retention in the TME (54, 55), and IL21, a cytokine recently reported to be involved in Tfh cytotoxicity in neoadjuvant NSCLC immunotherapy (2), were enriched in module 41, demonstrating that neo-PD1-pulsed terminal differentiation of CD4^+^ T cells is accompanied by enhanced tumor-killing potential.

In contrast, Monocle3 had two origins for CD8^+^ TILs: CD8.c05 and CD8.c10. ZNF683, the core marker of CD8.c10 (**Supplementary Figure S1I**), was recently proven to serve as a marker of responding Trm cells following ICI therapy in HNSCC (9) and Richter syndrome (56) and is indeed located at relatively early differentiation stages in TIL trajectories (48) (**Figure 1J**). Additionally, the differentiation pathway prevailed along with TUB4A vanishing, PRF1 and GZMH enhancing for both origins (**Supplementary Figures S1F, G**). For the trajectory of CD8.c05 to CD8.c11 and CD8.c12, specific distinct features were observed in CD8.c11 compared to those in CD8.c12 (**Figure 1K**). Two exhaustion signatures, one with CD52, CCL5 and LSP1 (**Figure 1L**) and the other with CXCL13, ENTPD1 and LAYN (**Figure 1M**), served as characteristics of CD8.c11 and CD8.c12, respectively, indicating varying degrees of activation. The CD8.c11 signature increased earlier along the trajectory (**Figure 1N**) and decayed rapidly in CD8.c12 cells, followed by a steep increase in the CD8.c12 signature. Indeed, we identified an effector-exhaustion mixture state signature (Miller et al. (57)) enriched in CD8.c11 via a meta-analysis of literature-reported signatures (**Supplementary Table S3**), while a burned-out state of CD8.c12 characterized by Braun_et_al_terminal_differentiation and Braun_et_al_Inhibitory signatures (58) was observed (**Figure 1O**). This further confirmed the rationality of a more significant expansion of CD8.c11 than CD8.c12 in MPR-E.

As has been verified, CD4.c16, CD4.c17, CD8.c11 and CD8.c12 can be categorized into transitional states according to both functional features (Azizi_et_al_Pro_inflADMmatory signature: CCL4, CCL5, PRF1 and IFNG, etc.) and exhaustion features (Azizi_et_al_Immunoselective signature: LGALS1, IGTGAE, CD5 and ENTPD1, etc. (59)), with CD8.c12 and CD4.c16 being relatively more exhausted (**Figure 1O**). In contrast, CD8.c05 was located at the origin of CD8^+^ TIL differentiation and was enriched with the Miller_et_al_progenitor_exh signature (**Figure 1O**); however, it was still difficult to acquire sophisticated functional annotations through the Molecular Signatures Database (MSigDB), scCODE marker identification, or Monocle3 trajectory analysis.

### 3.2 ADGRE5 pathway enhancement from non-to-MPR-E

Since Zada et al. successfully identified mregDC as an indispensable cluster for refueling CXCL13^+^PD1^+^ CD4^+^ Th1 through PIC-seq (60), we wondered whether cell-communication analysis could shed light on the dissection of the MPR-E meta-cluster, especially for CD8.c05.

**Figure 2:**
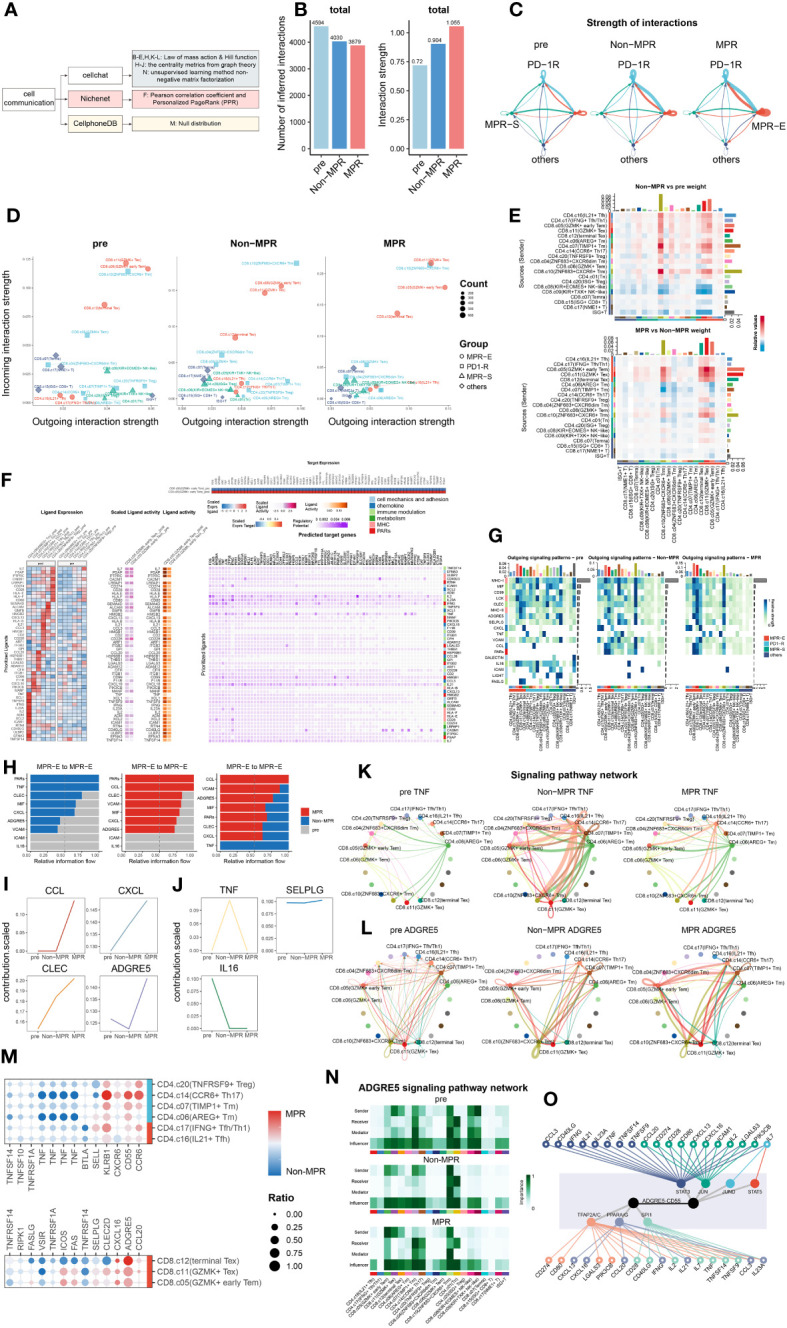
ADGRE5 pathway enhancement from Non to MPR in MPR-E. **(A)** Summary of cell-communication pipeline composed of three methods. **(B)** The total number (left) or strength (right) of CellChat-inferred interactions among the population in each group (light blue: pre, blue: Non, red: MPR). **(C)** Strength of significant ligand-receptor pairs between any pair of two meta-clusters. The edge width is proportional to the indicated strength of ligand-receptor pairs. **(D)** Comparing the outgoing and incoming interaction strength in 2D space in each group. Each dot denotes an individual cluster colored by the meta-cluster group. **(E)** Heat map of interaction strength comparisons of significant ligand-receptor pairs between any pair of two clusters. Red means upregulated in Non(upper) and MPR (down). **(F)** Ligands with top 50 prioritization scores (50-ligands) expression in CD4^+^T cells in MPR-E and PD1-R meta-clusters post and pre (left). The outcome of NicheNet’s ligand activity prediction and scaled ligand activity on DEGs upregulated in CD8.c05 within post-ICIs compared to pre-ICIs (middle). Scaled expression of target genes in CD8.c05 (right upper) and NicheNet’s ligand–target matrix denoting the regulatory potential between 50 ligands and target genes with meta-pathways annotation on the right (right lower). **(G)** The outgoing signaling patterns between CellChat curated pathways and defined cell clusters. The interaction strength reflects the sum of all normalized interactions in each pathway. The top bar graph and the right one summarize the interaction strength per cell type or per pathway respectively. The meta-pathways and meta-clusters were annotated on the left and bottom. **(H)** Select signaling pathways with significant differences in the overall information flow of MPR-E between specific pairs of pre, Non and MPR. **(I)** Scaled contribution of each pathway within MPR-E in pre, Non and MPR. **(J)** Scaled contribution of each pathway within MPR-E in pre, Non and MPR. **(K)** The inferred TNF signaling network among the cell populations represented by the nodes. Subclusters’ color distribution as in G, edge width representing the pathway-specific interaction strength. **(L)** Same as K for ADGRE5 pathway. **(M)** Dot plots showing expression of genes between MPR and Non coding for interacting ligand–receptor proteins (CellPhoneDBV3) in agreement with CellChat. **(N)** Heatmap showing the relative importance of each cell type based on the computed four network centrality measures of the ADGRE5 signaling network. **(O)** Integrated network of 19 signal mediators, 7 irTFs and ADGRE5-CD55 pathway from NicheNet.

Thus, to systematically explore the different aspects of cell communication between various meta-clusters longitudinally, an integrated cell-communication analysis pipeline composed of three methods, CellChat (31), Nichenet (61) and CellPhoneDBV3 (62), was developed (**Figure 2A**). Although we demonstrated that MPR-E significantly expanded in the MPR, it was inappropriate to arbitrarily assume that MPR-E served as the most prominent meta-cluster in terms of cell communication; thus, all 20 subclusters were included in further analysis.

We hypothesized that ICIs could reprogram the interaction mode of TILs based upon the knowledge of a “ligand–receptor pair” model for ICI response prediction developed by Huang et al. (63) Strikingly, although the inferred interaction amounts decreased from the pre to MPR, the interaction strength peaked in the MPR, which was used to assess the probability of certain interactions given the expression levels of the ligand–receptor pairs (L-Rs) (**Figure 2B**). In addition, communication in the MPR-E and PD1-R gradually became in the driving seat from pre to Non and then to MPR (**Figure 2C**, **Supplementary Figure S6A**). We further profiled the distribution of outgoing or incoming signals in specific clusters in each condition. Intriguingly, compared to that in the pre and Non groups, CD8.c05 and CD8.c11 in MPR-E, as well as CD8.c10 in PD1-R, predominantly occupied the most weights of interaction strength in MPR, measured by Euclidean distance between clusters (**Figure 2D**). Consistent with these findings, when comparing signaling between sources and senders quantitatively, MPR-E, especially CD8.c05 and CD8.c11, increased progressively from pre to non and then to MPR (**Figure 2E**, **Supplementary Figure S6B**). Moreover, all the other meta-clusters, such as CD8.c08 and CD4.c20 in MPR-S, quickly faded away, as measured by interaction strength. Overall, we noticed that, according to survival of the fittest, MPR-E gradually seized dominance in cell communication in the MPR group compared to that in the Non and Pre groups, especially for CD8.05 and CD8.c11, followed by CD8.c12, CD8.c10 and CD4.c16/17, which we termed as the “pruning effects” triggered by successful ICIs.

Given the prolific communication in MPR-E, we explored exactly which L-Rs participated in MPR. We adopted Differential NicheNet (33), an extension of the default NicheNet algorithm, to depict L-Rs specific for CD8.c05 and CD8.c11 post-ICIs, mainly focused upon ligands from CD4^+^ T cells in MPR-E and PD1-R, given the widely accepted concept of the delicate interplay between CD4^+^ and CD8^+^ T cells in the tumor immune cycle. However, an inevitable controversy would be the legitimacy of putting CD8.c05/11 TILs in the spotlight instead of CD4.c16/17. The CD4.c16/17 subset, a subset predicted to be CXCL13^+^PD-1^+^ (**Figure 1I**, **Supplementary Figure S1C**), was comprehensively explored by RNA sequencing of physically interacting cells (PIC-seq) developed by Zada et al. (60), a method for exploring physically interacting immune cells. They found that CXCL13^+^PD-1^+^ CD4^+^ T cells (equal to CD4.c16/17) augmented antitumor cytotoxicity through interaction with LAMP3^+^ DCs in tumor-draining lymph nodes (TDLNs). We reanalyzed the PIC-seq data (**Supplementary Figures S5A, B**) and confirmed the enhancement of the CD4.c16/17-LAMP3^+^DC interaction post-ICIs (**Supplementary Figures S5C, D**), possibly through MIF-(CD74^+^CXCR4)L-R (**Supplementary Figures S5F–H**); a detailed analysis of this interaction is provided in the **Supplementary Files**. Therefore, we instead placed poorly defined CD8.c05/11 cells at the center.

Nevertheless, indeed, ligands with the top 50 prioritization scores (50 ligands) exhibited significant cluster-specific upregulation of CD8.c05/11 in post-ICIs compared to that in pre-ICIs (**Figure 2F**, **Supplementary Figure S6C**); for example, SEMA4D in CD4.c17 has been linked to an imbalance of Th17/Treg cells with increased expression of IL-22 and RORyt in Th17 cells (64), and CCL3 in CD4.c16 has been considered an inducer of CD8^+^ T-cell proliferation in the TDLN (65). We then grouped the 50 ligands into 6 meta-pathways: cell mechanics and adhesion, chemokine, immune modulation, metabolism, MHC and PARs (**Figure 2F**). Taking a closer look at the predicted target genes related to the 6 pathways, we detected frequent immune-related transcription factor (irTF) activation in CD8.c05/11 cells. These activated irTFs included FOS-Jun, STAT5, and RUNX3 (**Figure 2F**), which are critical TFs responsible for CD8^+^ Trm redistribution that is repressed in CD4^+^ Trm cells (66).

To cross-validate and further refine the 50 ligands identified by NicheNet, we examined the signaling probabilities of all interactions previously identified by CellChat. In accordance with Nichenet, these interactions fell into the 6 meta-pathways. As proposed, the “pruning effects” of ICIs led to the enrichment of MHC-I/II, MIF, CLEC and ADGRE5 L-R in the CD8.c05/11/12 subgroup in the MPR cohort, while the expression of IL16, ICAM and FASLG L-R gradually decreased (**Figure 2G**, **Supplementary Figures S6D, E**, **S7A, B**). Considering that the interaction strength was more strongly enhanced in MPR than in Non, we focused on interactions within the MPR-E group that underwent stepwise enhancement from pre to Non then to MPR (**Figure 2H**) and identified CCL/CXCL, CLEC and ADGRE5 L-Rs (**Figures 2I, L**, **Supplementary Figures S7D, E**). Simultaneously, TNF, IL16 and SELPLG were characterized as the dominant L-Rs in the pre or Non (**Figures 2J, K**, **Supplementary Figures S7F, G**).

However, the abovementioned L-Rs were rather general; thus, we further explored the specific pathways enriched in these genes. CCL5-CCR4 contributed most to CCL L-Rs (**Supplementary Figure S7C**), which are responsible for the interplay between T cells/NK cells and T cells/endothelial cells post-ICIs (63). Moreover, neoantigen vaccines combined with ICIs can induce an antigen-specific CCL5^+^/CXCR3^+^ CD8^+^ T-cell population (67). CXCL13-CXCR3, a type of CXCL L-R, was upregulated significantly in MPR (**Supplementary Figure S7C**). The combination of features from CXCR3^+^CD8^+^ T cells and CD11c^+^ antigen-presenting cells (APCs) was shown to be associated with OS and progression-free survival (PFS) in patients with HCC (68). CLEC2B/C/D-KLRB1, which is a CLEC L-R (**Supplementary Figures S7C, S7E**), is closely associated with GZMB^+^ CD8^+^ T cells and has tissue homing properties (69). The IL-16 pathway was enriched in L-Rs of pre (**Figure 2J**, **Supplementary Figure S7F**), which compromised T-cell antitumor immunity by upregulating the coinhibitory receptor CD160 (70).

Among these L-Rs, ADGRE5-CD55 (**Figure 2H**) has rarely been investigated in the context of CD8^+^ T-cell communication during ICI therapy. Capasso et al. first identified the activation capacity of ADGRE5 on CD55^+^ CD4^+^ T cells (71), and recently, Abbott et al. confirmed that the CD55-ADGRE5 interaction between monocytes and T cells undermined T-cell responses (72). Recently, Felce et al. further demonstrated that ADGRE5^-/-^ CD4^+^ T cells are unable to respond to superantigens presented by DCs (73). Mechanistically, ADGRE5, which contains extracellular EGF-like repeats that mediate adhesion, localizes to and stabilizes immunological synapses (74). Despite being a well-defined participant in APC-CD4^+^ T-cell interactions, in-depth research on the role of ADGRE5 in tumor immunology is lacking.

Before the thorough exploration of ADGRE5 in CD8.c05 cells, we confirmed that the expression of ADGRE5, CXCL and CCL L-Rs was higher in MPR than in Non by CellPhoneDBV3 based on the principle of the null distribution, with ADGRE5 exhibiting the greatest increase (**Figure 2M**). Notably, we detected cluster-specific differences in ADGRE5 from pre to Non and then to MPR. Notably, ADGRE5 was exclusively enhanced in MPR-E during ICIs (**Figure 2L**), especially in the CD8.c05, in which the most significant enhancement in the ADGRE5 L-R was observed in MPR compared with that in Non. Moreover, ADGRE5 L-R in CD8.c05 was the only one that participated in all four components of L-Rs according to CellChat (sender, receiver, mediator and influencer) analysis compared to all the other subclusters (**Figure 2N**).

Afterward, we adopted centrality metrics from graph theory in Nichenet to identify dominant contributors to the intercellular communication networks of ADGRE5-CD55 (**Figure 2O**). Taking these irTFs into consideration, we narrowed the list of candidate molecules to “19 signaling mediators”, from CCL3 and IL21 to IL23A, and “7 irTFs”, including PPARA/G and STAT5 (**Figure 2O**), which are the most likely regulators of the ADGRE5-CD55 pathway.

The derivation process of ADGRE5 was extremely fragile due to the complex cell-communication analysis pipeline; thus, evidence from multimodal data became necessary. Nevertheless, interactome analysis revealed a “pruning effect” imposed by ICIs, with MPR-E acquiring an exclusive interaction strength above that of PD1-R or MPR-S, among which CD8.c05/11 contributed the most to the upregulation of ADGRE5-CD55, a critical L-R that might be mediated by irTFs.

### 3.3 Multimodal analysis revealing STAT5 regulation of ADGRE5

irTFs, as described by Nichenet, are vital for decoding the ADGRE5 regulatory network. In an attempt to identify specific irTFs that mediate the ADGRE5 pathway, the SCENIC method for determining TF activity was adopted. To enhance resolution, we performed a SCENIC analysis for each cluster in each patient separately and then performed integration. Unfortunately, although some canonical TFs specific to certain clusters, such as STAT2 in CD4.c16 (75), RORC in CD4.c17 (76) and RUNX3, were observed in CD8.c05/11/12 (77) (**Supplementary Figure S10A**), hardly any changes could be detected in MPR compared to that in Non, which was inconsistent with prior knowledge (78). We speculated that the lack of detailed and precise information due to the sparsity of the sc data and “gene dropout” hindered our inquiry (79). Sparsity is tolerable for molecularly distinct cell populations but fails to grasp subtle differences, as in our sorted CD3^+^ TILs. To handle sparsity, numerous strategies, such as imputation methods (80, 81), paired bulk RNA-seq data (82), and VIPER, have been developed. VIPER was generated by Obradovic et al. (83) The protein activity data revealed that C1Q^+^TREM2^+^APOE^+^ macrophages were an indicator of early postsurgical disease recurrence in RCC patients, a finding that was unavailable merely by sc, which was validated by CITE-seq and qmIF. Inspired by this circumstance, we took advantage of VIPER to replenish our existing sc data.

**Figure 3:**
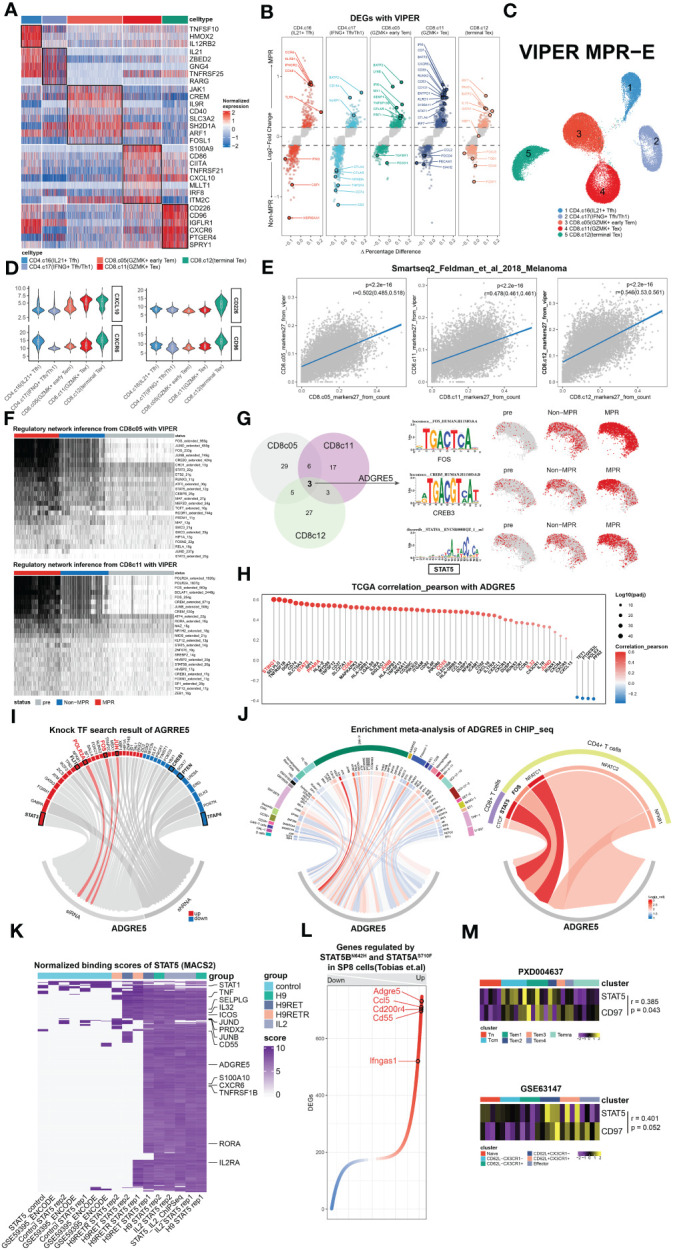
Multimodal analysis revealing STAT5 regulation of ADGRE5. **(A)** Heat map of DEGs found in clusters in MPR-E with VIPER. **(B)** Volcano plot showing DEGs between MPR and Non from MPR-E clusters with VIPER. Each colored dot denoting an individual gene with adjusted P value < 0.05 (scCODE) and |log (Fold change)≥ 0.2. **(C)** UMAP of MPR-E clusters from VIPER; n = 50,037 cells. **(D)** Violin plots showing normalized expression of selected genes in VIPER for MPR-E clusters. **(E)** Relationship of 27 markers of CD8.c05/11/12 from VIPER versus corresponding markers calculated from sc count data through scCODE; fitting with a linear regression model. P values were determined by a two-sided linear regression t-test. **(F)** SCENIC results on CD8.c05/11 with VIPER-pro after filtering; TFs were ranked by the expression levels in MPR. **(G)** Comparison and overlap of TFs from CD8.c05/11/12 (left) and the motif of 3 irTFs (middle) and expression of them in CD8.c05/11/12 in pre, Non and MPR (right). n = 36,412 cells. **(H)** Pearson correlation coefficients of ADGRE5 with all the other genes in pan-cancer TCGA database; ranked by coefficients value. P values were determined by a two-sided linear regression t-test. **(I)** TFs that could upregulate ADGRE5 or not from KnockTF database. **(J)** Meta-analysis of CHIP-seq datasets involving ADGRE5 from different cell types (data index in **Supplementary Table S4**). **(K)** Heat map of genes’ binding scores in STAT5 CHIP-seq with different treatments (calculated through MACS2). **(L)** Genes that were found significantly (P-adj<0.01) up- or downregulated mutually between STAT5A^N642H^ or STAT5B^S710F^ vs. WT mice in SP8 cells (CD8 single-positive T cells). **(M)** Heatmap showing STAT5-ADGRE5 axis among different types of CD8^+^ T cells from two independent proteomics datasets, shown as averaged scaled gene expression for each sample.

We thus used the VIPER pipeline to infer protein activity and generated 3,122 additional proteins (VIPER-pro) for our sc dataset. After the integration of the original transcriptomic data, we managed to acquire clearer clustering effects for MPR-E than for sc in terms of umap visualization and DEG heatmaps (**Figures 3A**, **3D**), before scCODE-irFilter (Methods) or after (**Supplementary Figures S9A, B**). For marker detection, VIPER indeed offered additional biological insights. For example, IL-9R (**Figure 3A**), which is considered a marker of newly discovered Th9 cells (84) and contributes to the expansion of CD8^+^ TILs after ICIs (85), was found to be differentially activated in CD8.c05 cells. VIPER successfully profiled delicate differences between subpopulations and between MPR and Non (**Figures 3B–D**). BATF2, upregulated in CD8.c05 cells in the MPR (**Figure 3B**), was shown to be essential for epigenetic reorganization of chromatin during CD8^+^ Teff differentiation in cooperation with RUNX3 and IRF4 (86).

Similar to our speculation, the results obtained with VIPER-pro were further corroborated by Pearson correlation analysis of the transcriptomic cluster markers in an independent Smart-seq dataset focused on ICIs (**Figure 3E**, **Supplementary Figure S9E**). Proteins ranked within 27 in each MPR-E cluster were taken into account because they exhibited the most significant correlation with the original cluster markers while showing the slightest correlation with markers from other clusters simultaneously (a data processing procedure named “VIPER-irFilter”, see in Methods) (**Supplementary Figures S9D, E**).

Therefore, approximately 135 VIPER-pro proteins were ultimately incorporated into the VIPER-based SCENIC process; these proteins could be retained in MPR-E after VIPER-irFilter. Notably, VIPER-irFilter, considered an exercise dealing with the abovementioned sparsity, greatly improved the capacity of SCENIC to capture the transition of irTF programs from Non to MPR (**Figure 3F**). Strikingly, such a transition did not occur inside CD4.c16/17 cells (**Supplementary Figure S10B**), indicating that CD8^+^ TILs undergo significantly more intense reprogramming than CD4^+^ T cells (87).

Afterwards, following scCODE-based filtering of TFs with log2FC>0.2 (MPR versus Non), we excluded those that were upregulated in pre and retained those exhibiting cluster-specific characteristics. While Fos-Jun (AP-1) dominated the reconstruction of CD8.c05, POLR2A played a leading role in CD8.c11, and FOXN2 gradually prevailed in CD8.c12 (**Figure 3F**, **Supplementary Figure S10B**). With the function of the latter two remaining unknown in tumor immunology, Giuliana et al. recently confirmed that AP-1 is indispensable for the prevention of NFAT1-induced T-cell exhaustion (88).

To extrapolate the SCENIC results to ADGRE5 L-R in CD8.c05/11, we hypothesized that candidate irTFs responsible for ADGRE5 regulation should meet 3 criteria: 1) these irTFs constantly appeared in all three CD8^+^ subpopulations; 2) they underwent gradual downregulation along the trajectory from CD8.c05 to CD8.c12; and 3) they experienced stepwise enhancement from pre to Non to MPR. After taking the intersection, AP-1, CREB3 and STAT5 were ultimately selected for further exploration (**Figure 3G**, **Supplementary Figures S10C, D**).

As demonstrated in the VIPER-irFilter, we adopted a methodological strategy featuring cross-validation of informative reference data with real-world sequencing data; thus, verification of SCENIC-derived TFs with TCGA data was conducted. We performed a Pearson correlation analysis of ADGRE5, CD55 and IL32 in the pan-cancer TCGA database (**Figure 3H**, **Supplementary Figures S10E, F**). Although absent in NicheNet, CellChat or VIPER according to our own analysis, IL32 is considered an activator of ADGRE5 on CD8^+^ T cells according to mFC (89); thus, it was temporally incorporated into our analysis. Astonishingly, STAT5 was tightly correlated to ADGRE5 in the TCGA cohort, following STING1, the core molecule in the cGAS-STING axis that amplifies I-IFN via the TBK1-IKK-NFkB pathway in antitumor immunity (90). In addition to STING1, the aforementioned enriched L-Rs, such as MHC-I/II (CD74, HLA-E, MYD88, MAPKAPK3, etc.), CCL/CXCL (CCL18, CXCL16, CX3CL1, etc.), and NicheNet-derived irTFs (SPI1, PPARA, JUNB/D, etc.), as well as IL32, were closely correlated with ADGRE5, while ADGRF5 linked with IL32 further confirmed the potential STAT5-mediated regulation of ADGRE5 under IL32—which remained a postulation until the KnockTF database and public CHIP-seq data were obtained.

We then questioned exactly which TFs manipulate the ADGRE5 pathway. First, we identified TFs that decreased ADGRE5 expression via KnockTF, which is a comprehensive database with manually curated datasets containing 308 TFs disrupted by different knockdown and knockout techniques (91). Notably, siRNA-mediated disruption of AP-1 dampened ADGRE5 expression, as did that of POLR2A, while CREB1, which has a similar function as CREB3, positively regulated ADGRE5 instead (**Figure 3I**). Unfortunately, STAT5 was not detected in the KnockTF; thus, a meta-analysis of cell type-based Chip-seq datasets was performed (**Supplementary Table S4**). Among the 29 cell types we collected, comprehensive heterogeneity across TFs that control ADGRE5 fate was recapitulated. With SPI1 appearing to be a specific modulator in macrophages, K-562 (human chronic myelogenous leukemia cell line) cells instead rely upon multiple TFs, from AP-1 to FOXJ3. Moreover, with NFATC1 dominating in CD4^+^ T cells, STAT5 appeared to control ADGRE5 in CD8^+^ T cells, surpassing CTCF completely (**Figure 3J**).

Specifically, STAT5 is composed of two highly correlated proteins, STAT5A and STAT5B (92), since most related studies consider STAT5/B as a whole (92–94); thus, in the following analyses, we included STAT5A/B-related datasets.

Based upon the Chip-seq data, we investigated whether STAT5 interferes with ADGRE5 expression. The IL-2/IL-2R pathway is a well-defined axis that depends on STAT5-mediated signals (92). Recently, a PD-1-cis IL-2R agonist that boosts PD-1^+^TCF-1^+^ stem-like CD8^+^ T cells was developed (95), the targeted cells of which were similar to those of CD8.c05 in our analysis. We hypothesized that IL-2R agonists could be an alternative option for reproducing neo-PD1 effects on the STAT5-ADGRE5 axis due to the lack of such Chip-seq data.

**Figure 4:**
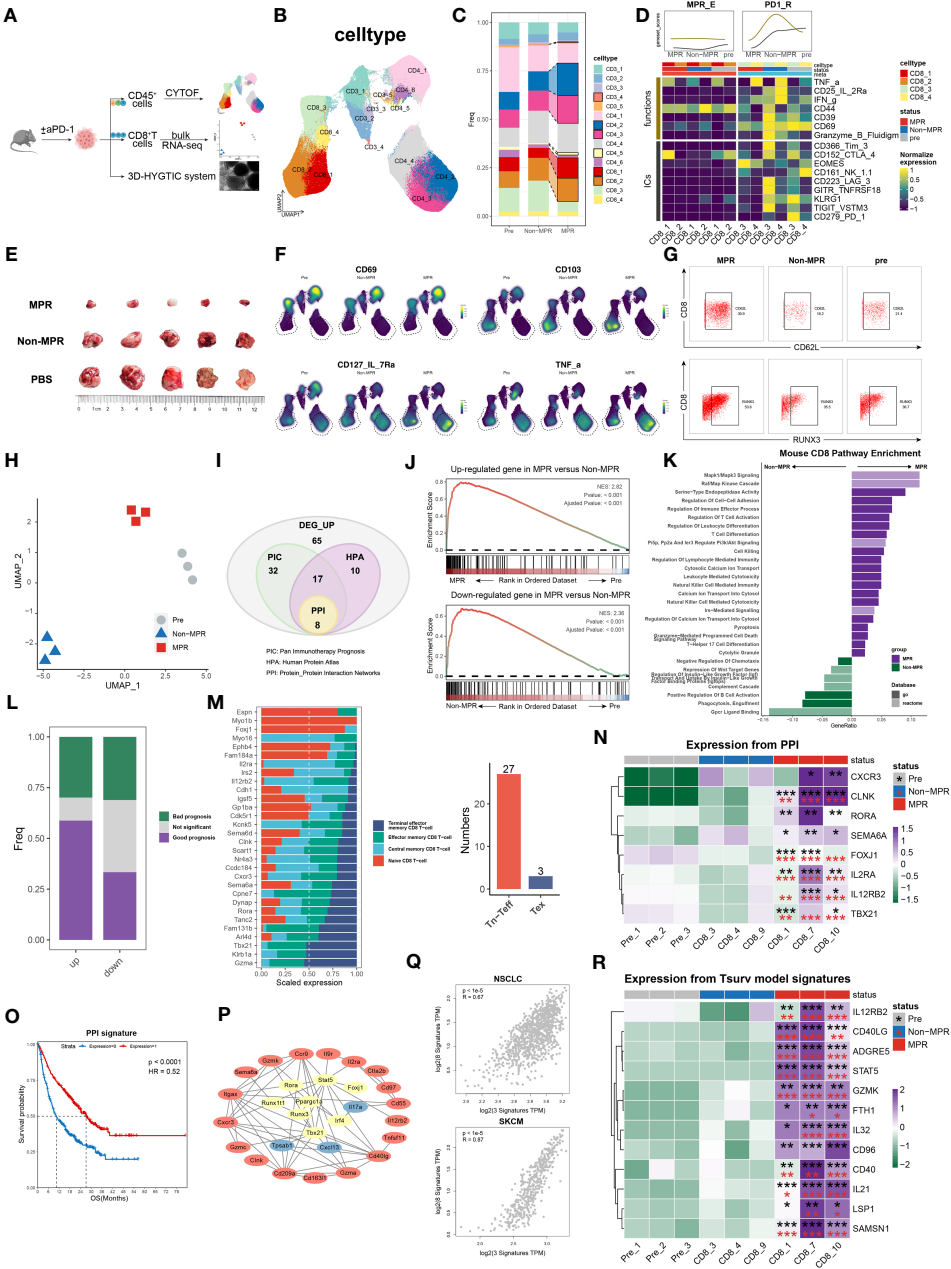
survT sub-cluster in MPR-E mapped across human to murine TME. **(A)** Workflow for sample processing and CYTOF or bulk-RNA-seq and 3D-HYGTIC construction from murine ICI-resistance model (method). **(B)** UMAP of cell clusters from integrated CYTOF of sorted 318,324 CD45^+^ TILs. **(C)** Bar plots of cluster distribution among cells from Pre, Non or MPR. **(D)** Heat map showing functional markers and ICs expression in murine MPR-E and PD1-R. The top smooth lines fit the sum of average expression of functional markers and ICs in each condition. **(E)** Tumor picture for each group (n = 5 mice/group). **(F)** UMAP plots showing the expression levels of certain signature genes. **(G)** CD62L and RUNX3 expression in MPR, Non and Pre from CD8_1 T cluster. **(H)** UMAP of murine CD8^+^ T cells from bulk RNA-seq data (n = 3 samples/group). **(I)** Venn diagram of overlap of DEGs unregulated in MPR versus Non, genes involved in PIC, HPA and PPI. PIC, Pan Immunotherapy Prognosis; HPA, Human Protein Atlas; PPI, Protein-Protein Interaction Networks. **(J)** GSEA results for DEGs up- or downregulated in MPR versus Non in murine CD8^+^ TIL. **(K)** Pathway enrichment analysis of DEGs from murine MPR and Non in GO and REACTOME database and pathways with q-value < 0.01 were shown here. **(L)** Bar plots of genes distribution involved in PIC among DEGs up- or downregulated in MPR versus Non; good prognosis (purple) meaning positive correlation between gene expression level and longer OS in PIC dataset, with bad prognosis (green) meaning the opposite; survival analysis of OS calculated by log-rank test (P-value<0.05 considered significant). **(M)** Bar plots of genes distribution in HPA-based analysis among naive CD8^+^ T cell (Tn), central memory CD8+ T cell (Tcm), effector memory CD8^+^ T cell (Tem), terminal effector memory CD8^+^ T-cell (Tex) (left). Numbers of genes were summarized on the right. **(N)** Heat map of normalized RNA expression through qRT-PCR of genes in the PPI signature. Statistical testing between samples in MPR and CD8_3 (Non, red asterisks) or Pre_1 (Pre, black asterisks) by two-sided t-test (*p < 0.05, **p < 0.01, ***p < 0.001). **(O)** Kaplan-Meier plots showing the PPI signature indicating better OS in PIC. Hazard ratios (HR) were calculated using Cox proportional hazards regression models, and p-value was calculated using log-rank test. **(P)** Significant PPI of the intersection genes of PIC and HPA **(I)** and IL32-ADGRE5-CD55 axis with node colored by their function, red: **(L–R)**, yellow: TFs, blue: others. **(Q)** Relationship of the PPI signature and IL32-ADGRE5-CD55 in TCGA datasets (LUAD, SKCM). P values were determined by a two-sided linear regression t-test. **(R)** Heat map of normalized RNA expression through qRT-PCR of genes in the Tsurv signature upon murine CD8^+^ TIL, except for IL32 that was tested in patients’ CD8^+^ TIL (method). Statistical testing between samples in MPR and CD8_3 (Non, red asterisks) or Pre_1 (Pre, black asterisks) by two-sided t-test (*p < 0.05, **p < 0.01, ***p < 0.001).

H9 is a “superkine” IL2 that augments IL-2Rβ signaling more profoundly than IL2, and H9RET behaves as a partial agonist, while H9RETR serves as a nonagonist (96). With the integrated analysis of STAT5 Chip-seq data, we revealed significant upregulation of ADGRE5 downstream of STAT5 in combination with IL2 or H9 (**Figure 3K**). Astonishingly, only one replication of H9RET retained activation of ADGRE5, H9RETR and the control groups all failed, while IL32 remained strongly enriched under both conditions, which indicated that the regulation of STAT5 by ADGRE5 was stricter than that by IL32. Nevertheless, IL2-mediated STAT5 activation provides indirect evidence to some extent, and we further reanalyzed DEGs from Tobias et al. They constructed the “gain-of-function” mutations STAT5A^N642H^ and STAT5B^S710F^ in CD8^+^ T cells via CRISPR/Cas9 and performed bulk RNA-seq on wild-type CD8^+^ T cells (97). Again, ADGRE5 was upregulated in STAT5^N642H^ and STAT5^S710F^ cells (**Figure 3L**), further confirming the positive regulatory effect of STAT5 on ADGRE5. Finally, since proteins, rather than RNAs, are executors of specific biological activities, we also validated the STAT5-ADGRE5 axis via proteomics datasets. Along the trajectory from Tn, Tcm to Tem and finally to Temra, STAT5 expression peaked within the Tcm and Tem1 stages, located at relatively early differentiation paths, followed by ADGRE5 activation ranging from Tcm to Tem4; these two genes were significantly correlated, as was the case for CD62L^+^ CD8^+^ T cells (**Figure 3M**). Since the phosphorylation of STAT5 (p-STAT5) definitely reveals its activity more precisely, which was not detected here, we hypothesized that the measurement of p-STAT5 would be more accurate and have shown this phenomenon (**Figure 4**).

It should be mentioned that Temra and CD62L^-^ CD8^+^ T cells were both considered as exhausted activation state of T cells, the relatively low expression of ADGRE5 and STAT5 in these T cells further consolidated our aforementioned conclusions.

### 3.4 survT subcluster in MPR-E mapped across the human to murine TME

CD8.c05 in MPR-E, as described above, was a stem-like CD8^+^ TIL cluster that expanded in the MPR, characterized by enhanced STAT5-ADGRE5 activity. However, whether precursor CD8^+^ TILs or terminally differentiated CD8^+^ TILs respond to neo-PD-1 therapy remains controversial. Similarly, Zhang et al. proposed that, compared with renal cell carcinoma (RCC), NSCLC with lower co-inhibitory ligands (CIL score) pre-ICIs is associated with more precursor-like CD8^+^ TILs post-ICIs in the MPR (16), which is consistent with our analysis. We thereby designed an LLC-based murine model to verify our findings *in vivo*. We managed to group mice that underwent the same regimen of anti-PD1 therapy into either MPR or Non according to tumor size, and CD45^+^ TILs or CD8^+^ TILs were sorted for CYTOF or bulk RNA-seq analysis, respectively (**Figures 4A, E**).

After non-redundancy score (NRS)-based selection of CD45, CD44, CD3e, CD4, CD8a and TNFa, CYTOF achieved well classified population identification (**Figure 4B**, **Supplementary Figures S11A, B**). In support of the sc analysis, murine CD3^+^ TILs also exhibited recalibration, with CD8_1, CD8_2, CD4_3 and CD4_4 significantly expanding in the MPR compared to that in the Non or pre groups (**Figure 4C**). Under the same paradigm, murine CD3^+^ TILs were categorized into murine MPR-E, PD1-R, and MPR-S TILs and others (**Figure 4C**, **Supplementary Figure S11C**). In addition, similar to the somewhat indescribable CD8.c05 identified during MSigDB annotation, murine MPR-E also exhibited a sustained ‘silent’ phenotype with weakened functional markers such as CD39, CD69 or ICs such as PD-1, TIGIT, CTLA-4 and KLRG1 (**Figure 4D**, **Supplementary Figures S11C**, **Figure S13A**), indicating a quiescent state even post-ICIs. Intriguingly, compared with those in pre or MPR, CD8_c3 and CD8_c4 in murine PD1-Rs exhibited upregulation of functional markers and ICs in Non (**Figure 4D**, **Supplementary Figure S13D**). In contrast, the expression of stemness markers (IL-7R and RUNX3) and resident-memory markers (CD62L, CD103, and RORg) significantly increased exclusively in MPR compared to that in Non, a phenotype similar to that observed for CD8.c05 in human MPR-E (**Figures 4F, G**, **Supplementary Figures S11D, E**). For the convenience of harmonization, we labeled CD8_c1/2 in murine MPR-E and CD8.c05 in human MPR-E as survT, indicating that T cells preserved the stemness of surviving ICIs under TME pressure.

Due to the limited use of CYTOF markers, bulk RNA-seq was performed for sorted CD8^+^ TILs. UMAP dimensionality reduction analysis revealed considerable heterogeneity (**Figure 4H**, **Supplementary Figure S12A**). A total of 132 upregulated genes in the MPR versus Non (DEG_up) were included in further exploration (**Figure 4I**) after demonstrating their significant enrichment in MPR compared with that in Non via GSEA (**Figure 4J**). Astonishingly, except for T-cell activation and differentiation pathways, DEG_up revealed enhanced enrichment of cell−cell adhesion, indicating potential synapse formation and T-cell migration (**Figure 4K**). Additionally, the human CD8^+^ MPR-E signature was significantly more prevalent in murine MPR than in murine pre (**Supplementary Figure S12F**). Although the CD8.c05 signature was moderately enriched in murine MPR (NES=1.31, P value=0.1), it still strongly outperformed the CD8.c11 signature (NES=1.03, P value=0.39), as well as the CD8.c12 signature (NES=0.77, P value=0.91), further supporting our notion that MPRs prefer stem-like survT rather than terminally functioning Tex, such as CD8.c12, in both the murine TME and the human TME.

To bridge the gap between the molecular features of human and murine TMEs, we utilized the Monaco et al. sc dataset from the Human Protein Atlas (HPA) (98) and integrated pan-cancer immunotherapy cohort datasets (PIC, **Supplementary Table S1**) to project DEG_up into the human ICI scenario. We identified 30 genes among the DEGs with measurable expression along the T-cell differentiation trajectory in the HPA cohort (Methods) (**Figure 4I**). Strikingly, 27 of the 30 genes exhibited higher expression in Tn, Tcm or Tem cells than in Tex cells (**Figure 4M**). In addition, DEGs with favorable prognoses were much more prevalent in the PIC than in the DEG_down (genes down-regulated in MPR compared to Non) subgroup (**Figure 4L**), further supporting the robustness of our murine model. After the intersection of the genes identified via PIC and HPA analyses, a protein−protein interaction (PPI) network was constructed, and the 8-gene signature (PPI signature) with the highest combined score was identified (**Figure 4I**). The PPI signature showed intertwined communication with the aforementioned IL32-STAT5-ADGRE5 axis (**Figure 4P**) and was an indicator of improved OS (**Figure 4O**), even when considered separately (**Supplementary Figure S11D**). Additionally, the PPI signature exhibited a significantly strong correlation with the IL32-STAT5-ADGRE5 signature in multiple TCGA datasets (LUAD, SKCM, BRCA, HNSC, etc.) (**Figure 4Q**). Finally, qRT−PCR was conducted to verify the PPI signature, which was robustly upregulated in MPR compared with both Non and Pre (**Figure 4N**, **Supplementary Figures S13B, C**). Overall, these findings not only confirmed the existence of a species-conserved survT as an MPR-responding cluster post-ICIs in lung cancer but also supplemented additional multimodal data, including CYTOF and bulk RNA-seq data, to support our conclusions.

### 3.5 Verification of the IL32-STAT5-ADGRE5 axis in patients in the MPR-E cohort in an independent ICI cohort

Theoretically, direct induction analysis of sorted CD3^+^ or CD45^+^ TILs indeed abolishes interference from other cell types and improves the resolution of the sparse sc matrix but also introduces imprecision into the data analysis when taking everything in account. We thus analyzed our own cohort of NSCLC patients with all TME cells (Methods). Altogether, 9 cell types were well represented in our own samples, among which neutrophils (S100A8 and CXCL8) and CAFs (COL6A3, FN1, and POSTN) were rather difficult to capture due to primary cell fragility and scarcity (**Supplementary Figures S14A, B**).

**Figure 5:**
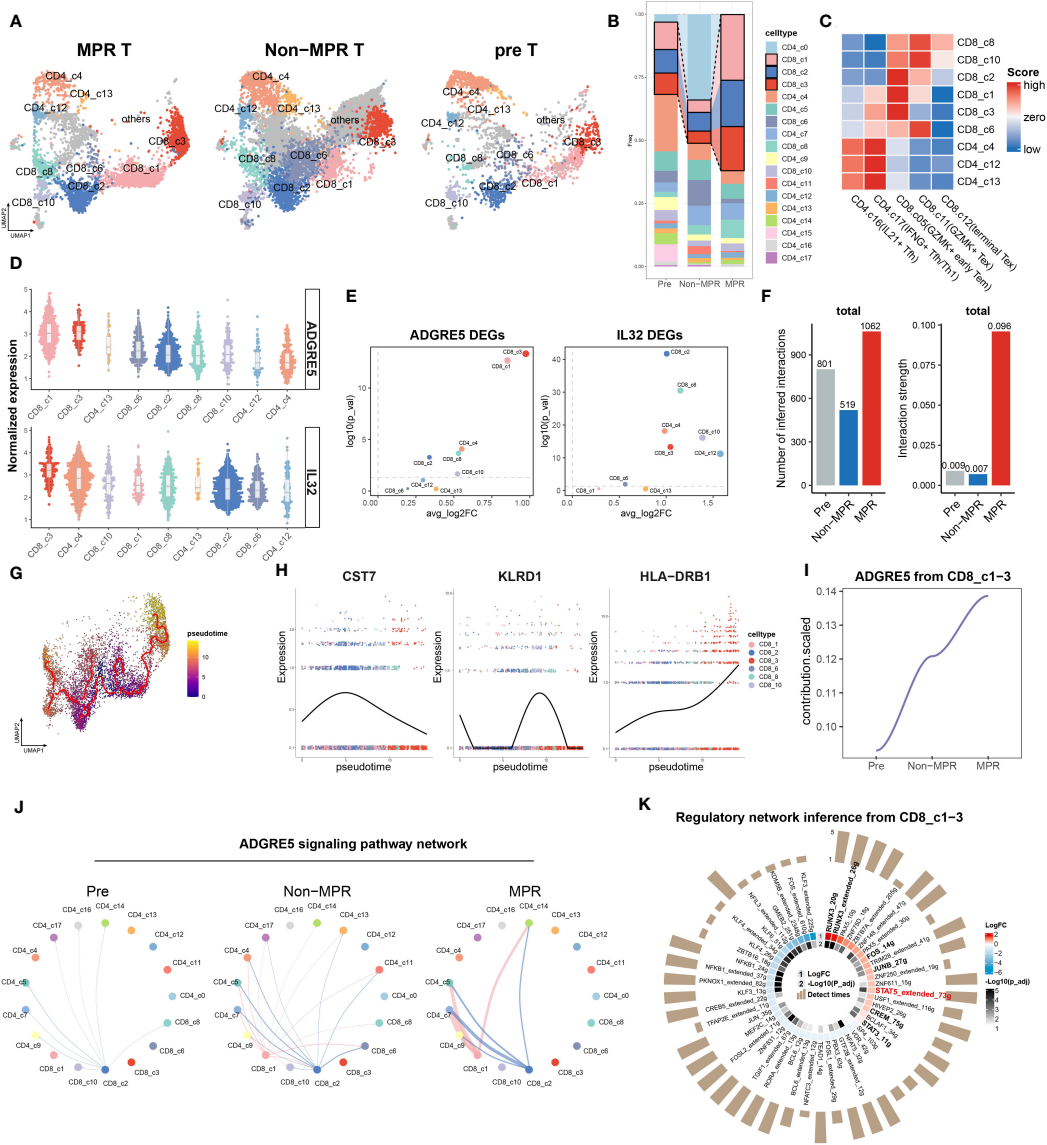
Verification of IL32-STAT5-ADGRE5 axis in MPR-E in an independent ICIs cohort. **(A)** UMAP of 14,819 T cells in MPR, Non and Pre from an independent NSCLC patients’ cohort. **(B)** Bar plots of T cells clusters distribution across MPR, Non and Pre. **(C)** Heat map showing correlation scores calculated by SingleR between certain clusters from B with MPR-E in **Figure 1C**. **(D)** Comparison of the AGDRE5 and IL32 expression in different cell clusters from C, with color coded by clusters. **(E)** Volcano plot showing differential expression of ADGRE5 and IL32 between MPR and Non in cell clusters from C, color coded by clusters. **(F)** The total number (left) or strength (right) of CellChat-inferred interactions among the population in each group (gray: pre, blue: Non, red: MPR). **(G)** CD8^+^ T cells colored by pseudotime inferred by Monocle3 (n = 6,680 cells). **(H)** Scatter plot of certain signature genes among clusters listed (right legend) ordered along pseudo-time. Points are colored by cell clusters. **(I)** Scaled contribution of ADGRE5 pathway within MPR-E in pre, Non and MPR. **(J)** The inferred ADGRE5 signaling network among the cell populations represented by the nodes; edge width representing the pathway-specific interaction strength. **(K)** scCODE results of TFs from SCENIC on CD8_c1-3 between MPR and Non.

In addition to the predominance of T cells in MPR compared to that in Non or pre, moderate expansion of B cells was also detected, while endothelial cells and macrophages were more prevalent in Non than in MPR (**Supplementary Figures S14C, D**). We further divided T cells into 18 clusters, via Louvain clustering, (**Figure 5A**), among which CD8_c1/2/3/8 were substantially enlarged in MPR compared to those in Non and pre, while CD8_c10 exhibited slight expansion in the MPR compared to that in Non but exhibited a contraction compared to that in pre (**Figure 5B**). Importantly, the extremely close correlation of CD8_c1/2/3 with CD8.c05 in MPR-E reinforced the indispensable role of survT in MPR, once again, with CD8_c8/10 in relation to CD8.c11 (**Figure 5C**, **Supplementary Figure S14E**). Although we did not observe prominent CD4^+^ subpopulation expansion in MPR, CD4_c4/12/13 cells exhibited great similarity with CD4.c16/17 cells (**Figure 5C**, **Supplementary Figure S14E**). In summary, we considered CD8_c1/2/3 as survT in this independent dataset and included CD8_c8/10 and CD4_c4/12/3 in further investigations due to the correlation analysis above.

To further validate the ADGRE5 pathway, we examined ADGRE5, IL32, CD55 and Nichenet-derived “19 signal mediators” in the clusters mentioned above. Unfortunately, except for TNF, 18 signaling mediators remained unchanged in MPR compared to Non, and CD55 was downregulated in MPR (**Supplementary Figure S14F**). Due to the inconsistency of CD55 expression pattern within this validation cohort compared to that in the discovery sc dataset above, we next investigated the potential role of IL32 in the regulation of ADGRE5.

Astonishingly, IL32 together with ADGRE5 exhibited significantly higher expression in MPR than in Non within almost all the clusters (except for CD4_c13), which was closely correlated with the MPR-E (**Figure 5E**, **Supplementary Figure S14G**). Strikingly, CD8_1/3 had the highest ADGRE5 expression (**Figure 5D**) and the greatest difference between MPR and Non (**Figure 5E**). Although CD8_c2-related IL32 expression was rather moderate (**Figure 5D**), its expression in MPR markedly surpassed that in Non (**Figure 5E**). In addition, the differential trajectory (originating at the interface of CD8_c06 and CD8_c02 on the umap plot) from CD8_c01/02 to CD8_c03 depicted by Monocle3 was rather similar to that of the discovery sc dataset (**Figures 1J**, **5G**), with CST7 and TUBA4A peaking at CD8_c02 and HLA-DRB1 prevailing at CD8_c3 (**Figure 5H**, **Supplementary Figure S15C**). Interestingly, CXCL13, KLRD1 and GNLY, markers of exhausted and activated TILs, were dominant in CD8_c8 cells and indeed showed the highest correlation with CD8.c12 in MPR-E cells (**Figure 5C**, **Supplementary Figure S15C**).

According to the L-Rs analysis, we accidentally uncovered “pruning effects” beyond CD3^+^ TIL populations. In fact, the interaction amount and strength decreased substantially after ICI therapy; instead, the interaction within T cells increased tremendously, especially in the MPR group, indicating the invincible status of T cells in cell communication (**Figure 5F**, **Supplementary Figures S15A, B**). Closer examination of the specific clusters responsible for “pruning effects” revealed that CD8_c2 outperformed the other clusters and manifested stepwise upregulation of ADGRE5 signaling from pre to Non, together with CD8_c1 (**Figure 5I, J**). Finally, VIPER-based SCENIC inference of irTFs, as mentioned above, revealed STAT5 in survT within MPR compared to that in Non (**Figure 5K**), further supporting the previously revealed role of the STAT5-ADGRE5 axis.

Overall, we reconstructed our analysis pipeline on our independent NSCLC ICIs cohort and successfully mapped the STAT5-ADGRE5 axis to verify the results, incorporating IL32 as a potential regulator.

### 3.6 ADGRE5 regulation by STAT5 is dependent on IL32, as revealed by the 3D HYGITC system

**Figure 6:**
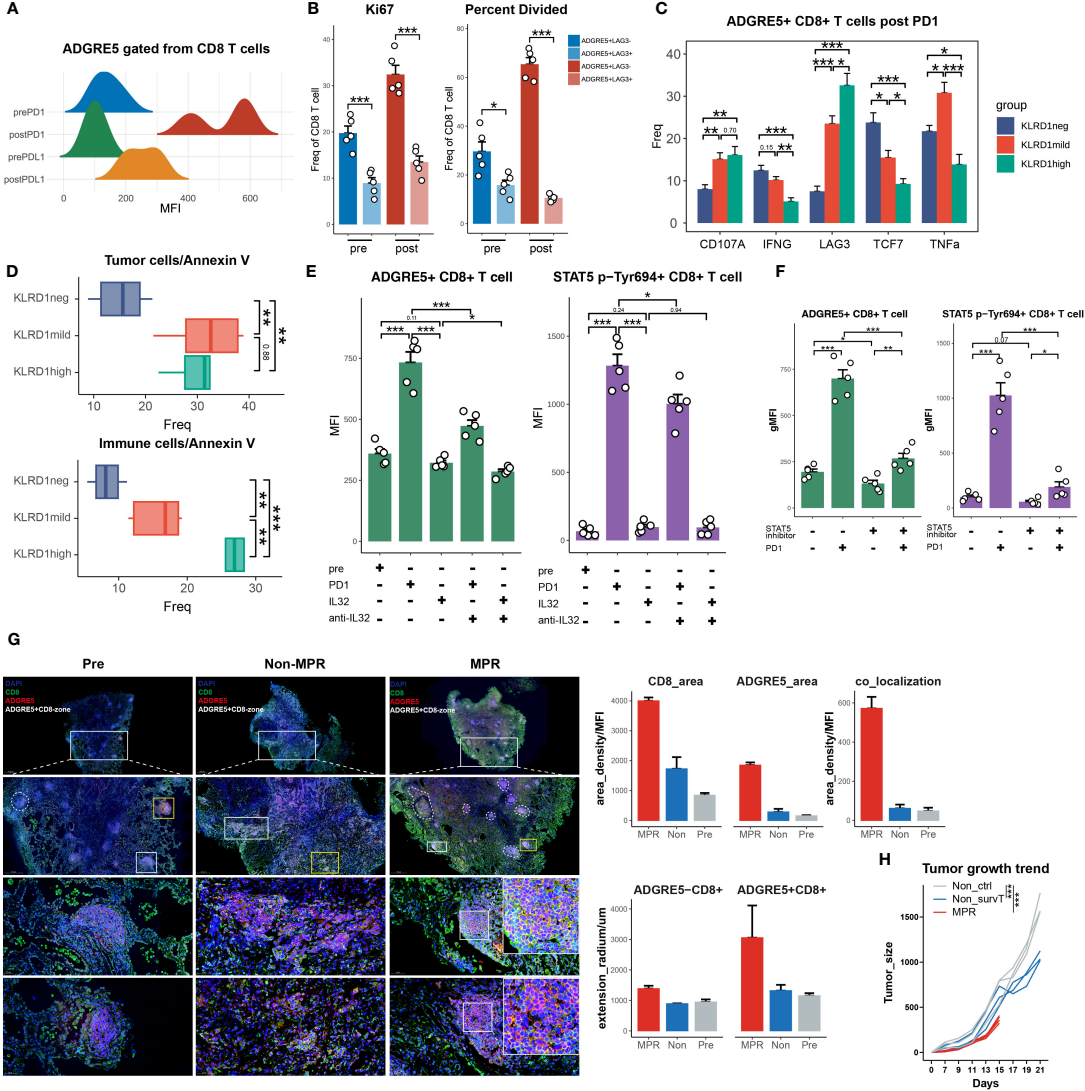
ADGRE5 regulated by STAT5 dependent on IL32 revealed in 3D HYGITC system. **(A)** Ridge plot showing MFI of AGDRE5^+^CD8^+^ T cells pre- and post-PD1 or PD-L1. **(B)** Flow cytometry analysis of Ki67 expression and CFSE results on ADGRE5^+^ or ADGRE5^-^ CD8^+^ T cells pre- and post-PD1. **(C)** Flow cytometry analysis of specific markers’ expression on KLRD^neg^, KLRD^mild^ and KLRD^high^ CD8^+^ T cells gated from AGDRE5^+^CD8^+^ T cells. **(D)** Flow cytometry analysis of calcium-AM of tumor cells (upper) or immune cells (down) in KLRD^neg^, KLRD^mild^ and KLRD^high^ CD8^+^ T cells (gated from AGDRE5^+^CD8^+^ T cells) in separate 3D-HYGTIC systems (method); **(E)** Bar plot showing MFI of ADGRE5 or STAT5 p-Tyr694 expression on CD8^+^ T cells under the anti-PD1, IL32 or anti-IL32. **(F)** Bar plot showing MFI of ADGRE5 or STAT5 p-Tyr694 expression on CD8^+^ T cells under the anti-PD1 or STAT5_IN_1 (STAT5 inhibitor). **(G)** Representative images of mIF from Pre, Non and MPR patients (left). Scale bar, 1000 μm (upper panel), 500 μm (middle panel), 50 μm (lower panel) (left). Cell density and extension radium of ADGRE5^+^CD8^+^ T cells (n = 2) (upper right). **(H)** LLC-tumor-bearing mice treated with anti-PD1 antibody. On day 15, survT sorted from MPR reinfused into Non through intratumoral injection (Non-survT), with non-survT into Non-ctrl (method). Each line represents an individual mouse. (n = as least 3 mice/group, Kaplan–Meier method, two-way ANOVA test). Data in all quantitative panels, except for G and H, are displayed as the mean ± SEM. n = 5, *p < 0. 05, **p < 0.01, ***p < 0.001, two-tailed unpaired t test. T.

The 3D-HYGTIC system is a well-designed nonimmunogenic ex vivo platform for tumor-immune interaction studies, as has been proposed (20). We hypothesized that ADGRE5 could be stimulated by anti-PD-1 through the activation of STAT5 according to previous investigations. Therefore, we constructed 3D-HYGTIC from murine TME-derived tumor fragments (MDTF; methods) and utilized TILs to carry out coculture experiments with or without anti-PD-1 therapy. Indeed, anti-PD-1 therapy strongly increased ADGRE5 expression on CD8^+^ T cells (**Figure 6A**). Strikingly, anti-PD-L1 therapy, on the contrary, had moderate effects, indicating potential specific anti-PD-1 mechanisms (**Figure 6A**).

According to our previous analysis, the ADGRE5-characterized survT manifested stemness phenotype with expansion in MPR. We then wondered whether ADGRE5^+^ CD8^+^ TILs might have improved proliferation ability compared with that in ADGRE5^-^ CD8^+^ TILs. In fact, Ki-67 upregulation in the ADGRE5^+^ CD8^+^ TIL population not only confirmed its proliferative potential but also suggested that it was a subcluster that responded to anti-PD-1 rather than ADGRE5^-^ CD8^+^ TILs (**Figure 6B**, **Supplementary Figure S16A**). In addition, this population also exhibited enhanced production of effector molecules (CD107a, IFNγ, TNFa) as well as stemness markers (TCF-7) post-ICIs therapy (**Supplementary Figure S16D**). In addition, exhausted CD8 TILs could also boost a certain degree of antitumor cytotoxicity (99) at the cost of activation-induced cell death (100). Astonishingly, more ADGRE5^+^ CD8^+^ TILs survived ICIs and exhibited comparable antitumor potential compared to that of ADGRE5^-^ CD8^+^ TILs (**Supplementary Figure S16E**), suggesting that these cells have a more favorable stemness phenotype even within the TME exposed to ICIs.

Specifically, the ADGRE5^+^ CD8^+^ TIL cluster should be heterogeneous, as should the CD8^+^ MPR-E cluster, and we hypothesized that LAG-3 upregulation post-ICIs (**Supplementary Figure S16D**) could be attributed to a more specific exhausted subcluster inside the cluster. First, we further separated ADGRE5^+^ CD8^+^ TILs into LAG3^+^ and LAG3^-^ subclusters according to the differential expression of LAG3 across CD8.c05/11/12 (**Supplementary Figure S16B**). Indeed, compared with LAG3^+^ TILs, LAG3^-^ TILs exhibited an even greater proliferation ability in the carboxyfluorescein succinimidyl ester (CFSE) dilution assay and Ki67 staining (**Figure 6B**, **Supplementary Figure S16C**). Noticeably, although LAG3^-^ cells were capable of identifying CD8.c05 from ADGRE5^+^ CD8 TIL to some extent, CD8.c11 and ZNF683^+^ CD8^+^ TIL in PD1-R all showed enhanced LAG3 expression (**Supplementary Figure S16B**); thus, we further used KLRD1 instead of LAG3 to depict CD8.c05 and CD8.c11 more precisely. Astonishingly, KLRD1^neg^ or KLRD1^mid^ ADGRE5^+^ CD8 TIL cells exhibited a better survival rate than KLRD1^high^ cells under TME selection pressure (**Figure 6D**). KLRD1^mid^ cells exhibited antitumor cytotoxicity comparable to that of KLRD1^high^ cells, with more preserved TCF7 (**Figure 6C**). KLRD1^neg^ cells exhibited a certain degree of activation and markedly improved stemness, as indicated by increased TCF7 expression, decreased LAG3 expression and decreased apoptosis (**Figures 6C, D**). In conclusion, KLRD1^neg^ ADGRE5^+^ CD8 TILs could be considered stem-like survT, as mentioned above, while KLRD1^mid^ and KLRD1^high^ cells could be mapped to CD8.c11 and CD8.c12, respectively.

To better profile survT, we sorted them out and examined whether the modulatory effect of anti-PD1 therapy on ADGRE5 was dependent upon IL-32 and STAT5. Here, we used patient-derived tumor fragment (PDTF)-based 3D HYGTIC because of the unavailability of murine anti-IL32 antibodies (method). First, anti-PD1 therapy successfully enhanced ADGRE5 expression, as did STAT5 phosphorylation at Tyr694 (p-Tyr694 STAT5) (**Figure 6E**). Although supplementation with IL32 could not further stimulate ADGRE5 expression or p-Tyr694 STAT5, inhibition of IL32 after anti-PD1 therapy indeed abrogated the anti-PD1-dependent upregulation of ADGRE5 and STAT5 p-Tyr694 (**Figure 6E**). Furthermore, we validated IL32-STAT5-ADGRE5 regulatory axis by western blot (WB), ELISA and qRT-PCR. Importantly, post-anti-PD-1 MPR samples showed significantly elevated IL32 expression (**Supplementary Figure S25A**). Our WB analysis of 20 samples indicated enhanced expression of both p-STAT5 and ADGRE5 in MPR, both pre and post anti-PD-1 compared to Non (**Supplementary Figure S25B**). Notably, a strong positive correlation between p-STAT5 and ADGRE5 was consistently observed and confirmed by PCR analyses (**Supplementary Figure S25C**). What’s more, we further utilized Jurkat cell line to prove our findings. Two experimental platforms were established: one comprised solely of Jurkat cells, and the second was integrated into A549-based tumor 3D HYGTIC models. Consistent with our primary specimen results (**Supplementary Figures S25A-C**), IL32 demonstrated a noticeable increase following anti-PD-1 treatment (**Supplementary Figure S25D**). Additionally, ADGRE5 and p-STAT5 exhibited significant up-regulation as confirmed by WB and PCR (**Supplementary Figures S25E, F**). Moreover, in accordance with our mFC results, while IL32 alone did not enhance ADGRE5 and p-STAT5 expression, the inhibition of IL32 (anti-IL32) abolished the effects of anti-PD-1. This provides evidence that the anti-PD-1-stimulated STAT5-ADGRE5 axis is indeed dependent on IL32. Additionally, STAT5_IN_1, a STAT5-specific inhibitor, strongly suppressed STAT5 phosphorylation and significantly reversed the increase in ADGRE5 expression after treatment with an anti-PD1 antibody (**Figure 6F**). Overall, these findings suggested that anti-PD1 therapy stimulates ADGRE5 expression via survT, which is regulated by STAT5 phosphorylation and dependent upon IL32.

Overall, ADGRE5 itself was found to be a responsive marker for anti-PD1 therapy and was found to be a meta-cluster with improved proliferation ability and effector function. We next performed multiplex immunofluorescence (mIF) analysis of tumor sections paired with our own aforementioned ICIs cohorts to verify ADGRE5 in a clinical setting (**Figure 6G**, **Supplementary Figure S17**). In terms of spatial infiltration depth as well as density, CD8^+^ TIL in the MPR group exceeded that in Non and pre groups enormously, as well as for ADGRE5 (**Figure 6G**). Notably, ADGRE5 was not exclusively expressed by CD8^+^ TILs. We next calculated the colocalization of CD8 and ADGRE5 further, and ADGRE5^+^ CD8^+^ TILs were significantly enriched in MPR compared with that in pre and Non. In addition, rather than exhibiting a diffuse distribution in Non, ADGRE5^+^ CD8^+^ TILs accumulated in tertiary-lymphoid-structure-like (TLS-like) cell clusters and deeper into the interior tumor bed in the MPR group (**Figure 6G**), some of which presented alongside the tumor microvasculature (**Supplementary Figure S17**). This unique distribution pattern of ADGRE5 not only revealed its correlation with the improved antitumor ability of CD8^+^ TILs but also indicated probable enhanced budding, adhesion and migration of CD8^+^ TILs from the peripheral circulation system into the TME. We previously developed a stepwise digestion process for 3D HYGTIC in which we were able to compare different TIL infiltration modes outside tumor spheroids (outer), at tumor boundaries (inner), or inside tumor core areas within 200 µm (core). Interestingly, in 3D HYGTIC, ADGRE5 exhibited significantly higher expression in the inner compartment than in the outer segment, while the infiltration of ADGRE5^+^ CD8^+^ TILs into the core still exhibited moderate expression (**Supplementary Figure S16F**), which was in accordance with our mIF findings.

Since we have successfully verified the distinct antitumor behavior of ADGRE5^+^ CD8^+^ TILs in MPR patients and in 3D HYGTIC, we decided to conduct adoptive cell therapy (ACT) in a previously established ICI-resistant murine model by reinfusion of survT from MPR mice into Non mice. As soon as the growth curves of MPR mice showed a significant difference with Non on day15 (**Figure 6H**), we sacrificed MPR mice and sorted survT (KLRD1^neg^ ADGRE5^+^ CD8^+^ TIL) from TME and reinfused them into Non mice through intratumoral injection (methods). Astonishingly, mice in the Non-survT ACT group began to exhibit significantly slower tumor growth than those in the Non-survT ACT group (**Figure 6H**). In conclusion, we further highlighted the translational application of survT as an ACT regimen in murine models, which is highly clinically significant considering the unsatisfactory outcomes of traditional ACT therapies in solid tumors, such as NSCLC.

### 3.7 ADGRE5-centered Tsurv model for ICI prognosis prediction and MPR classification

We utilized multimodal analysis of the aforementioned discovery dataset, including scCODE, Monocle3, cell communication (CellChat, Nichenet, and CellPhoneDBV3), VIPER and SCENIC, with elucidative gene sets generated at every step (for example, irTFs in Nichenet). The IL32-STAT5-ADGRE5 axis was thus identified, and the results revealed that MPR classification potential was good in the melanoma ICI cohort (**Supplementary Figure S18A**). In an attempt to generalize the favorable effects of survT to MPR for multiple cancer types, we managed to incorporate the gene sets generated above into a classifier model developmental pipeline (method).

**Figure 7:**
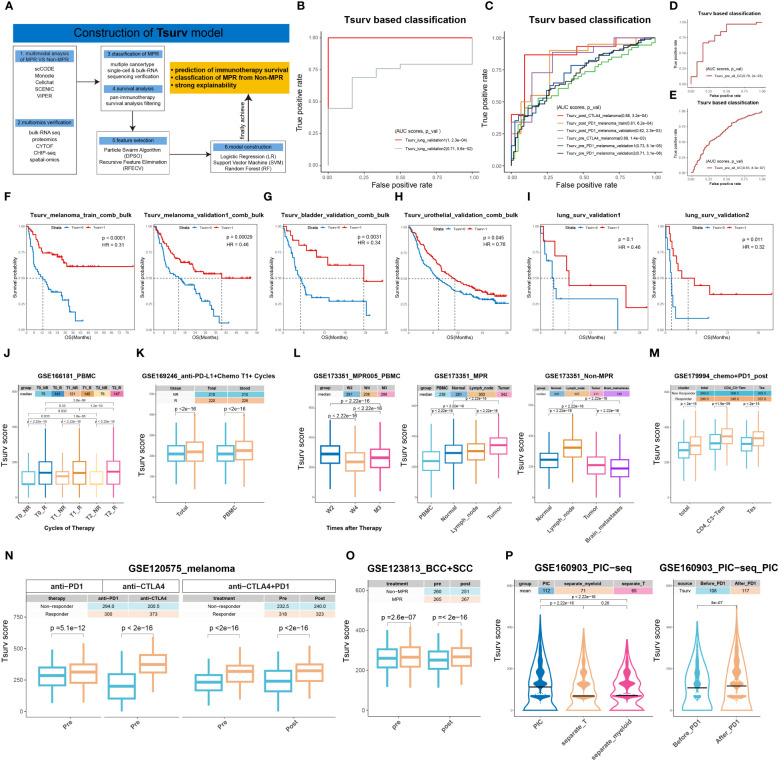
ADGRE5-centered Tsurv model for ICIs prognosis prediction and MPR classification. **(A)** Workflow for construction of Tsurv model. **(B–E)** Receiver operating characteristic (ROC) curves for the performance of Tsurv in NSCLC, SKCM, GC, UC with pre- or post-ICIs bulk-RNA-seq data. Data summarized from n = 16 and 35 samples from NSCLC; n = 26, 35, 51, 26, 92, 160 samples from SKCM (from above to below in the figure legend of **(C)**; n = 45 samples from GC and N = 345 samples from UC. **(F–I)** Kaplan-Meier plots for the performance of Tsurv in SKCM, BC, UC and NSCLC. Hazard ratios (HR) calculated using stratified Cox proportional hazards regression models, and p values calculated using a stratified log-rank test. Data summarized from n = 104 (train) and 145 samples (validation1) from melanoma; n = 73 samples from bladder cancer; n = 348 samples from UC and N = 35 (validation2) or 16 (validation1) samples from NSCLC. **(J–P)** Comparison of the Tsurv scores in cells from **Supplementary Table S1** (sheet2). Each dot representing one cell with the center line indicating the median value. The median of each group’s Tsurv scores shown in the top tables colored by each group. The lower and upper hinges representing the 25th and 75th percentiles, respectively, and whiskers denoting 1.5× interquartile range. Two-sided t-test. Data were summarized from n= 51,701 cells from GSE166181, n= 489,490 cells from GSE169246, n= 409,639 cells from GSE173351, n= 489,490 cells from GSE169246, n= 150,849 cells from GSE179994, n= 16,291 cells from GSE120575, n= 29,004 cells from GSE123813 and n= 31,450 cells from GSE160903. **(J)**, T0: at baseline; T1: at the first cycle of ICIs; T2: at the second cycle of ICIs. **(K)**, T1+: more than one cycles of ICIs; total: cells in PBMC, tumor and metastases. **(L)**, W2: two weeks after ICIs; W4: 4 weeks after ICIs; M3: three months after ICIs. **(P)**, PIC: physically interacting cells; separate_myeloid: single CD11c^+^ cells, separate_T: single CD3^+^ cells).

Generally, we integrated literature-reported ICI cohorts with paired prognosis information and bulk RNA sequencing data (**Supplementary Table S1**) into a PIC database and split them into a training set and a validation set for model fine-tuning (**Supplementary Table S1**). We subsequently collected genes associated with the MPR in the MPR-E meta-cluster and tested their ability to predict ICI prognosis and the MPR/PR classification. Afterward, the genes that remained were subjected to feature selection, model construction and parameter optimization. Finally, a model named Tsurv, composed of ADGRE5, IL32, STAT5 and 12 other genes, was generated (**Figure 7A**).

Strikingly, each individual Tsurv gene exhibited a significant ability to distinguish responders (R) from non-responders (NR) (area under curve (AUC): upper=0.8, lower=0.64) in the training dataset (integrated melanoma post-ICI cohort; **Supplementary Table S1**, **Supplementary Figure S18A**). In addition, qRT−PCR analysis of the Tsurv signature in the aforementioned CD8^+^ TILs from the murine model showed significant upregulation compared to that in Non or pre (**Figure 4R**). IL32, STAT5 and ADGRE5 exhibited AUCs of 0.8, 0.74 and 0.7, respectively (P value<0.001), and the overall Tsurv model had an AUC of 0.81 (P value=6.2e-04) (**Figure 7C**). Here, we should mention that the IL32-STAT5-ADGRE5 axis was present in the Tsurv model and was not artificially selected but was generated from the feature selection algorithm.

With respect to the Tsurv model, we first checked its performance in the NSCLC datasets. In two independent lung cancer cohorts with pre-ICIs bulk RNA-seq data, Tsurv had an outstanding AUC (validation 1 AUC: 1; validation 2 AUC: 0.71) (**Figure 7B**). Since melanoma was the exclusive tumor type for which enough pre-ICIs and post-ICIs were available (**Supplementary Table S1**), we selected the melanoma PIC to further validate the potential of Tsurv for identifying PRs in pre-ICIs and post-ICIs.

Notably, Tsurv exhibited remarkable predictive power in two independent melanoma ICI cohorts, pre-ICIs (validation 1, AUC=0.73; validation 2, AUC=0.71), and in one post-ICI validation dataset (AUC=0.82) (**Figure 7C**). However, the treatments for the validation cohorts mentioned above and for the training dataset were all anti-PD-1-based. We next examined Tsurv in anti-CTLA-4 settings. Moreover, Tsurv exhibited satisfactory performance in predicting anti-CTLA-4 responders (AUC of pre_anti-CTLA-4: 0.68; AUC of post_anti-CTLA-4: 0.88) (**Figure 7C**). Overall, the behavior of Tsurv remained stable pre-ICIs or post-ICIs, regardless of whether it was administered in combination with anti-PD-1 or anti-CTLA-4 therapy for melanoma. We further implemented tests in gastric cancer (GC) and urothelial cancer (UC) patients, and Tsurv again exhibited superior performance (GC AUC=0.78; UC AUC=0.65) pre-ICIs (**Figures 7D, E**). However, due to insufficient post-ICI samples from the remaining tumor types (**Supplementary Table S1**), we were forced to integrate post-ICI samples that were left altogether, and in this case, Tsurv failed to operate regularly (**Supplementary Figure S18B**). For esophageal cancer (ESCA) or HNSCC pre-ICIs, Tsurv was not suitable either (**Supplementary Figures S18 C, D**).

Generally, Tsurv is well-behaved in multiple cancer types for R/NR identification. We next evaluated its ability by performing Kaplan−Meier (KM) survival analysis. Tsurv not only performed well in the training dataset (HR=0.31, p value<0.0001) but also in three independent melanoma datasets (**Figure 7F**, **Supplementary Figure S18E, F**). In addition, Tsurv exhibited excellent prognostic value for bladder cancer (HR=0.34, p value=0.0031), UC (HR=0.76, p value=0.045) and NSCLC (validation 1: HR=0.32, p value=0.011) (**Figures 7G–I**). Tsurv performed rather generally in another NSCLC dataset and glioma cohort, mainly because of the limited sample size (**Supplementary Table S1**, **Figure 7I**, **Supplementary Figure S18H**). From another perspective, Tsurv might not be suitable for all tumor types; for example, it behaved poorly in ESCA (HR=1.64, p value =0.17) (**Supplementary Figure S18G**).

We further compared the performance of Tsurv with that of previously published predictive gene signatures focused on ICI cohorts. Most of them were developed for the prediction of OS from pre-ICIs, and within 5 signatures we tested (blood, IFN_gamma, IMPRES, inflammatory and T_cell_inflamed); all but IMPRESs exhibited little ability to identify R, with IMPRES incapable of predicting response (**Supplementary Figures S18I-M**). Tumor types and treatment regimens were chosen according to published articles (**Supplementary Table S1**), and all 5 signatures were not able to discriminate between PR and PD post-ICIs (**Supplementary Figures S1, 8I-M**). Overall, compared to published ICIs response prediction signatures, the Tsurv signature outperformed them and remained stable across different tumor types; moreover, its malleability and universality are rather rare.

Considering that Tsurv was developed from a restricted gene list that was actually derived from sorted CD3^+^ sc data, we evaluated the prediction power of Tsurv via the aforementioned bulk RNA-seq; thus, we hypothesized that Tsurv might preserve the ability to identify R/NR in sc datasets as well. Biasi et al. reported that CXCR4^+^ GZMB^+^ mucosal-associated invariant T (MAIT) cells in peripheral blood mononuclear cells (PBMCs) were more abundant in melanoma ICIs responders than in healthy controls. We calculated Tsurv model-derived scores from their PBMC-sc data and revealed that the Tsurv score was constantly upregulated in R patients compared with that in HCs before and throughout therapy (T0: baseline; T1: first cycle of ICIs; T2: second cycle of ICIs) (**Figure 7J**). In addition, compared with that at T0, NR exhibited a pulsed increase in Tsurv expression at T1, while R did not increase until T2, with a slight downward trend at T1.

For further validation, we adopted the BC sc dataset from Zhang et al. and the NSCLC sc dataset from Caushi et al., which all contained PBMC sc data. Additionally, Tsurv was significantly upregulated within MPR in the BC and NSCLC ICIs cohorts (**Figures 7K, L** left). In addition, we longitudinally observed the same fluctuation trend in the Tsurv score in NSCLC PBMCs (W4: at the second cycle of ICI therapy before surgery) (**Figure 7L**). We wondered whether transient downregulation of Tsurv at W4 in the MPR together with pulsed upregulation in the Non group could be a sign of survT (the CD8^+^ subcluster marked by our Tsurv signature) migration.

We thus hypothesized that after 2 cycles of ICI therapy, in MPR, survT would experience obvious mobilization from the peripheral circulation to the TME, while in Non, survT would be excluded from the TME (named the “Tsurv-migrate” phenomenon). Therefore, we calculated the Tsurv score from multiregional data at W4, including tumor-adjacent normal tissue (normal), tumor-draining lymph node (lymph node) and brain metastasis data. Astonishingly, the Tsurv score exhibited a significant stepwise upregulation trend in MPR from PBMCs to normal tissue, then to lymph nodes, and finally to the TME (**Figure 7L**). Moreover, the Tsurv score peaked in lymph nodes in Non but decreased from the lymph node to the TME and then to brain metastases (**Figure 7L**). Overall, the Tsurv score in MPR exceeded that in Non, regardless of the sampling region, except for in the lymph nodes in Non (**Supplementary Figure S19C** right). Notably, ADGRE5 was completely consistent with the Tsurv model, emphasizing its indispensable role in survT functionalization and the “Tsurv-migrate” phenomenon (**Supplementary Figure S19C** left and **S19D**).

Afterward, we examined the influence of different ICIs regimens on Tsurv performance in the sc datasets. In another NSCLC cohort that underwent anti-PD1+chemo therapy, Tsurv remained upregulated regardless of the cell subpopulation (**Figure 7M**, **Supplementary Figures S19E, F**). In addition, in melanoma, the Tsurv model performed well in the anti-PD1 regimen, anti-CTLA-4 regimen and combination therapy cohorts (**Figure 7N**). In patients with less aggressive BCC or SCC (nonmelanoma skin cancer), Tsurv also exhibited excellent performance (**Figure 7O**). Overall, these findings indicate that the Tsurv model is suitable for the treatment of various ICIs in clinical settings, further expanding the scope of its application.

Finally, taking advantage of the previously described PIC-seq dataset, we found that the Tsurv score was significantly higher in PICs than in separate T cells and manifested significant up-regulation in PIC post-ICIs compared to that in pre-ICIs (**Figure 7P**, **Supplementary Figure S19H**), indicating that survT might be dependent upon myeloid cells in the TME. Indeed, Cluster 0, which contained CXCL13^+^PD-1^+^CD4^+^ T cells, and Cluster 12, which represented LAMP3^+^ DCs (**Supplementary Figure S5**), were the only two clusters with significantly higher Tsurv scores post-ICIs and in the PIC than in the other subclusters (**Supplementary Figure S19I**).

Additionally, there is room for improvement in the Tsurv model. Frustratingly, Tsurv could not predict CD8^+^ TIL-specific potential for R in clear cell renal cell carcinoma (ccRCC) PBMCs or in the ccRCC TME, regardless of exploration in a multiregional matter (**Supplementary Figure S19A**). In BC, Tsurv lost the ability to predict R in PBMCs or the TME at T0 or T1 (**Supplementary Figure S19B**), with moderate efficacy of R/NR identification at T1+ (**Supplementary Figures S7K**, **S19B**). From another perspective, the limitations of Tsurv in BC and ccRCC were rather reasonable and explainable considering that Zhang et al. described them as CIL^high^ tumors that indeed suffer from reduction of precursor-like CD8^+^ TILs (phenotypically resembling survT) pre-ICIs and post-ICIs, in accordance with our Tsurv model behavior here. Finally, biological insights into the Tsurv model in non-T cells, such as CD45^-^ cells (**Supplementary Figure S19A**) and myeloid cells (**Supplementary Figure S19H**), still remained to be illuminated.

Overall, the ADGRE5-centered Tsurv model indicated that, as has been illuminated, could be used as a “multifunctional toolkit”, not only for excellent prediction of ICIs prognosis in multiple tumor types but also for PBMC-based liquid biopsy monitoring of the MPR/Non tract, even in biology insight mining, for instance, the “Tsurv-migrate” phenomenon and dependence of survT upon LAMP3^+^DC.

### 3.8 Spatial codependences of the IL32-STAT5-ADGRE5 axis

**Figure 8:**
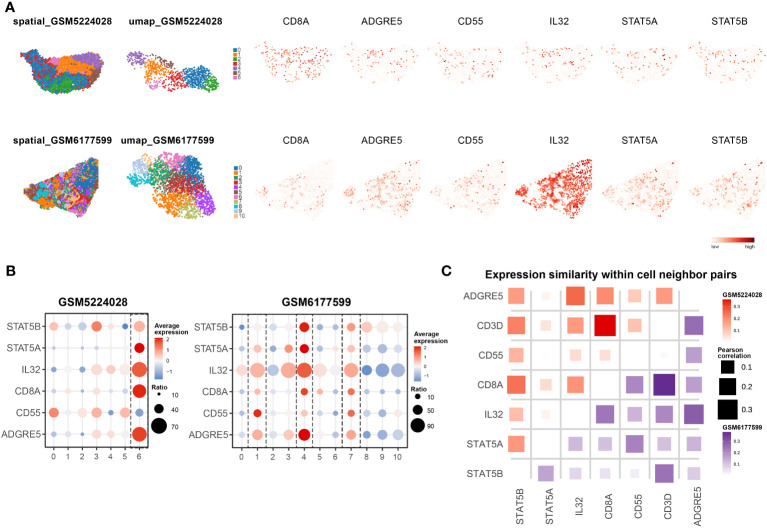
Spatial co-dependences of IL32-STAT5-ADGRE5 axis. **(A)** Spatial distribution (left) and UMAP plot (middle) of clusters in GSM5224028 (upper) and GSM6177599 (down), with feature plot of specific markers spatially (right). **(B)** Dot plot of genes expression of specific markers in clusters manifested in A from GSM5224028 (left) and GSM6177599 (right). **(C)** Expression similarity within cell-neighbor pairs.

We next sought to investigate whether the IL32-STAT5-ADGRE5 axis aligns with specific spatial distribution patterns in spatial transcriptomics data. After cluster discrimination and identification, we classified 7 clusters and 11 clusters in bladder cancer datasetA (GSM5224028) (101) and breast cancer datasetB (GSM6177599) (102), respectively. Although datasetA and datasetB were baseline nonmetastatic cancers that had not undergone ICI therapy (**Supplementary Figure S20**), we still observed obvious similarities in spatial distribution patterns across ADGRE5, IL32 and STAT5A/B (**Figure 8A**). To quantify spatial correlation, we utilized the Delaunay triangulation method to construct neighboring networks and then calculated the co-expression probability through the adjacency list (Methods). When computing the expression similarity within cell−neighbor pairs, we detected strong correlations between ADGRE5, STAT5, IL32 and CD8A in both datasetA and datasetB (**Figure 8C**). Notably, a co-expression pattern also appeared at the cluster level, as was the case for immune cell cluster 6 in dataset A and clusters 1, 4 and 7 in dataset B (**Figure 8B**). In conclusion, the spatial colocalization of the IL32-STAT5-ADGRE5 axis with CD8^+^ TILs further emphasized its probable regulatory network in the TME.

## 4 Discussion

Neo-PD1-based therapeutic strategies offer invaluable opportunities for temporal tracing of TME remodeling (11), which facilitates mapping specific beneficial cell subpopulations in the MPR to Non groups for overcoming ICI resistance (103). However, inter- and intratumor heterogeneity, together with the complexity of different immunotherapy combinations, have made this process challenging.

In this study, we managed to simplify the intricate scientific inquiry by specifically focusing on sc data derived exclusively from CD3^+^ TILs after 2 cycles of nivolumab treatment in resectable primary NSCLC patients. In adopting this approach, we ensured a more targeted and refined analysis, minimizing confounding factors, and successfully identifying IL32-STAT5-ADGRE5 as the axis responsible for the expansion of survT (a specific sub-cluster of MPR-E) within TME of MPR patients. To the best of our knowledge, this marks the inaugural recognition of the ADGRE5 pathway within the realm of tumor immunology.

In our analysis, we confirmed that MPR-E, composed of stem-like CD8.c05, effector-like CD8.c11 and CD4.c16/c17 and terminally-differentiated CD8.c12, was stepwise upregulated from pre to non-MPR and then to MPR. The debate over whether effector CD8, exhausted CD8, or stem-like CD8 constitutes the primary responsive sub-cluster to immunotherapy has persisted. Although both *in vitro*-expanded Teffs (≈ CD8.c11) and Tstems (≈ CD8.c05/survT) exhibited cytotoxicity to some extent in ACT, Meyran et al. confirmed that stemCAR-T cells (≈ CD8.c05/survT) with enhanced expression of SELL, TCF7 and RUNX2 were retained rather than conventional effector-like CAR-T cells that differentiated into anergic states rapidly *in vivo (* 104). Indeed, survT projected from stem-like CD8.c05 cells, compared with the others in MPR-E, exhibited the best consistency across murine and human multimodal validation.

To elucidate the mechanism driving the expansion of survT, we employed a cell communication analysis pipeline. This approach unveiled the ADGRE5 pathway between CD4^+^ T cells and survT in MPR-E, highlighting the significance of multicellular ecosystems in neo-PD1. Interestingly, CD8^+^ stemCAR-T cells failed to function without CD4^+^ stemCAR-T cells (104), and in another study, the TCF-1 Tpex was shown to increase and respond to neo-PD1 only in uninvolved regional lymph nodes (uiLNs) with help from DCs (15). Indeed, we also revealed physical interactions between LAMP3^+^ DCs and CXCL13^+^CD4 (CD4.c16/17 in MPR-E), possibly through MIF-(CD74+CXCR4), through reanalysis of a PIC-seq dataset (60). However, the direct involvement of ADGRE5 remained elusive. Although CD55, reported by Chang et al. to be potential ligand for ADGRE5 (89), exhibiting up-regulation in MPR-E in our discovery datasets, it did not consistently follow this trend in our own neo-PD1 sc datasets. Indeed, Chang et al. neither verified ADGRE5-CD55 interaction by ex-vivo experiments. This prompted us to leverage multimodal analyses ranging from Chip-seq to proteomics, which finally led to the discovery of IL32 and STAT5 as regulators of ADGRE5 expression.

Previous research has shown that IL32 has paradoxical behaviors. The microbiome stimulates protumorigenic IL32 expression in multiple myeloma (MM) cells via TLRs-NFkB (105), and Treg-derived IL32 promotes bladder cancer metastasis and immunosuppression together with TIGIT (106). In contrast, IL32 induces ICI-resistant melanoma by activating DCs to secrete CCL5-primed CD8^+^ TILs (105). In UC, IL32 promoted CD3^+^ TIL infiltration (107). However, the role of IL32 in CD8^+^ TILs in the neo-PD1 scenario has not been fully elucidated. In 3D-HYGTIC, IL32 served as the pillar for ICI-mediated upregulation of ADGRE5, without which ADGRE5 survT decreased exponentially, while IL32 addition did not upregulate ADGRE5. To explore the regulatory network of ADGRE5, we performed VIPER-based SCENIC analysis with ChIP-seq verification and subsequently suggested that STAT5 could be the core TF mediating ADGRE5 expression in survT.

In contrast to IL32 or CD55, STAT5 is a well-defined key TF involved in the antitumor effects of CD8^+^ TILs. Numerous newly discovered ICs, such as the methionine transporter SLC43A2 (108), protein tyrosine phosphatase 1B (PTP1B) (109), and the ubiquitin ligase MDM2, manipulate CD8^+^ TILs through STAT5 (110). Overexpression of STAT5 in the intermediate Tex epigenetically antagonized TOX in an LCMV infection mouse model (111). Moreover, little is known about the influence of STAT5 on neo-PD1 or the ADGRE5 pathway. We demonstrated that CD8.c05/11/12, although they share various degrees of exhaustion, enhanced STAT5 activation, together with CD8.c05 (named survT), was characterized by the most prominent enrichment of STAT5. In 3D HYGTIC, anti-PD-1 promoted ADGRE5 expression on survT through phosphorylation of STAT5 through a mechanism dependent on IL32. Consistent with our study, Wang et al. revealed that IL-2-treated CD8^+^ T cells quenched NFAT1-dependent PD-1 upregulation via competitive binding of the PD-1 promoter domain to STAT5 (112).

Overall, the IL32-STAT5-ADGRE5 axis serves as an essential pathway controlling survT, which makes us wonder whether this axis could be used to construct a classifier for MPR identification; thus, an ADGRE5-centered Tsurv model was constructed. Compared to existing immunotherapy prognosis models, some of which suffer from poor behavior in large cohorts or different tumor types, Tsurv performed markedly better in multiple ICI cohorts, both for pre-ICI prediction and post-ICI classification and was even capable of monitoring the MPR temporally with PBMC bulk RNA-seq data, further extending its translational capacity for low-cost, liquid biopsy-based identification of Non from MPR in the clinic.

Apparently, the limitations of our study are irrefutable. First, paired or extra sc TCR sequencing to decipher clonal expansion was absent, which was necessary for supporting MPR-E beyond a pure increase in the subcluster fraction. In addition, a better, well-designed CYTOF panel for the murine TME equipped with markers such as ADGRE5, IL32 and CD55 could be beneficial. Finally, the mechanisms responsible for the STAT5-ADGRE5 axis, as well as additional ligands for ADGRE5, have yet to be determined.

## Data availability statement

The original contributions presented in the study are publicly available. This data can be found here: https://zenodo.org/records/10096960.

## Ethics statement

The studies involving humans were approved by the Ethics Committee of Shanghai Chest Hospital (KS23014-(H)). The studies were conducted in accordance with the local legislation and institutional requirements. The participants provided their written informed consent to participate in this study. The animal study was approved by the Animal Experimental Ethics Committee of Shanghai Chest Hospital, Shanghai Jiaotong University, School of Medicine (project number KS23014-(A)). The study was conducted in accordance with the local legislation and institutional requirements.

## Author contributions

JL: Writing – original draft. ZM: Writing – original draft. ZC: Writing – review & editing. WL: Writing – review & editing. YY: Writing – review & editing, Funding acquisition, Resources. ZL: Writing – review & editing. SL: Writing – review & editing.
