# Supplementary material for: ADGRE5-centered Tsurv model in T cells recognizes responders to neoadjuvant cancer immunotherapy
Source: Front Immunol. 2024 Jan 26;15:1304183. doi: 10.3389/fimmu.2024.1304183 (PMC10853338; doi:10.3389/fimmu.2024.1304183)
Supplement: Supplementary file 2 [file DataSheet_2.pdf]

Supporting Information

ADGRE5-centered Tsurv model in T cells recognizes responders to neoadjuvant cancer immunotherapy

Jian Li[1]†, Zhouwenli Meng[1]†, Zhengqi Cao[1], Wenqing Lu[1], Ziming Li[1]*, Shun Lu[1]*

†These authors contributed equally to this work and share first authorship

[1]Shanghai Lung Cancer Center, Shanghai Chest Hospital, Shanghai Jiaotong University, School of Medicine, Shanghai, 200030, P. R. China

*** Correspondence:**
Shun Lu. Ziming Li
E-mail:
liziming1980@shsmu.edu.cn
shunlu@sjtu.edu.cn

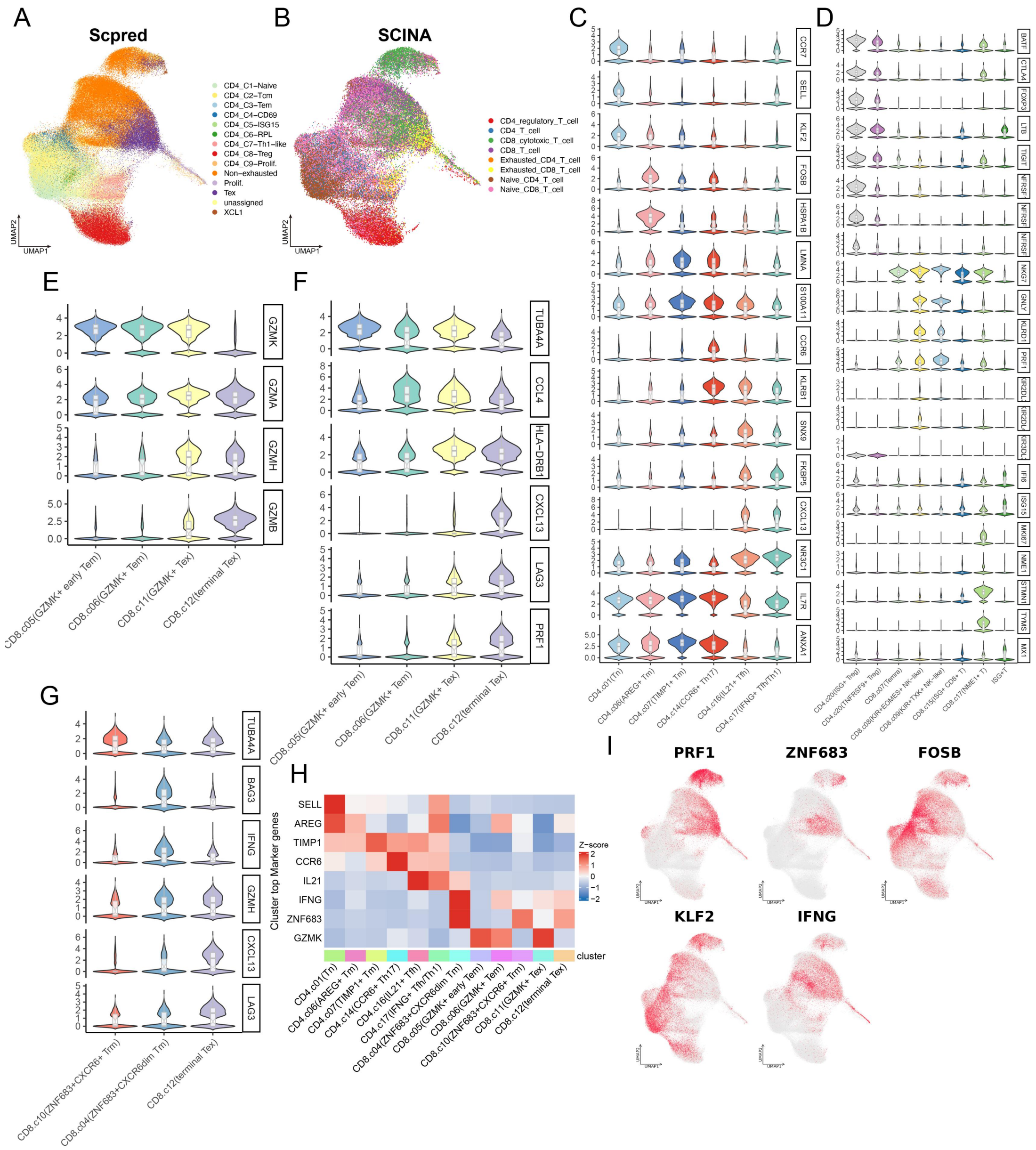

Supplementary Figure 1. Cluster annotation and feature genes presentation.

A. UMAP of cell clusters from integrated scRNA-seq data of 100,248 sorted CD3$^+$ T cells, which are further defined by ScPred.

B. UMAP of cell clusters from integrated scRNA-seq data of sorted 100,248 CD3$^+$ T cells, which are further defined by SCINA.

C-G. Vlnplot of differentially expressed genes found in each cluster.

H. Relationship of TIL atlas and remaining cell clusters.

I. UMAP feature-plots showing the expression levels of certain genes.

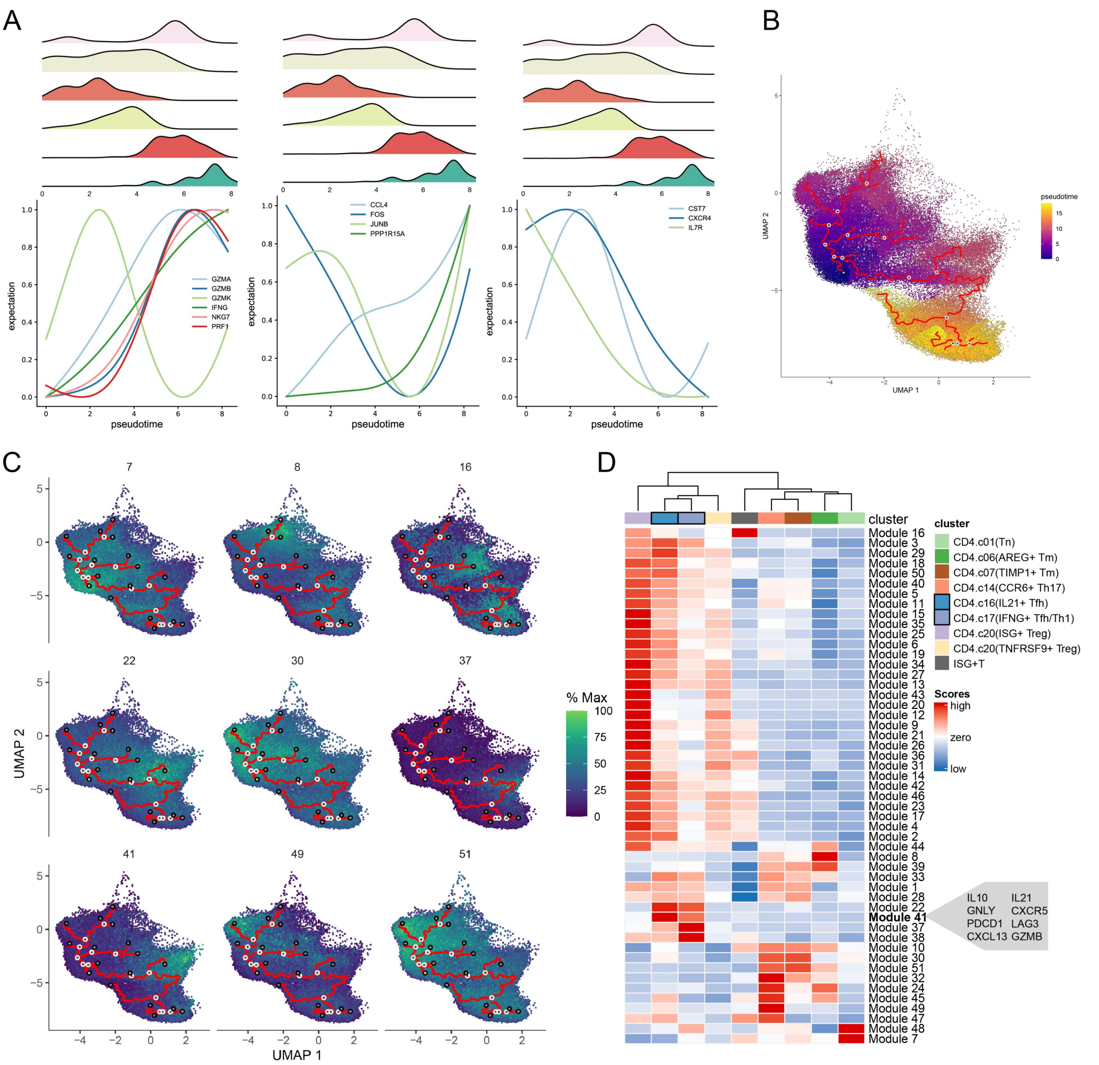

Supplementary Figure 2. Trajectories of CD8^{+} T cells and gene module identification of CD4^{+} T cells

A. The distribution of CD8^{+} T cells along with the pseudo-time (upper panel) and fitting curve for the expression levels of certain signature genes along the pseudo-time (lower panel).

B. 39,309 CD4^{+} T cells coloured by pseudotime inferred by Monocle3.

C. CD4^{+} T cells coloured by module scores.

D. Heat map showing scores of different module in CD4^{+} T cells.

A

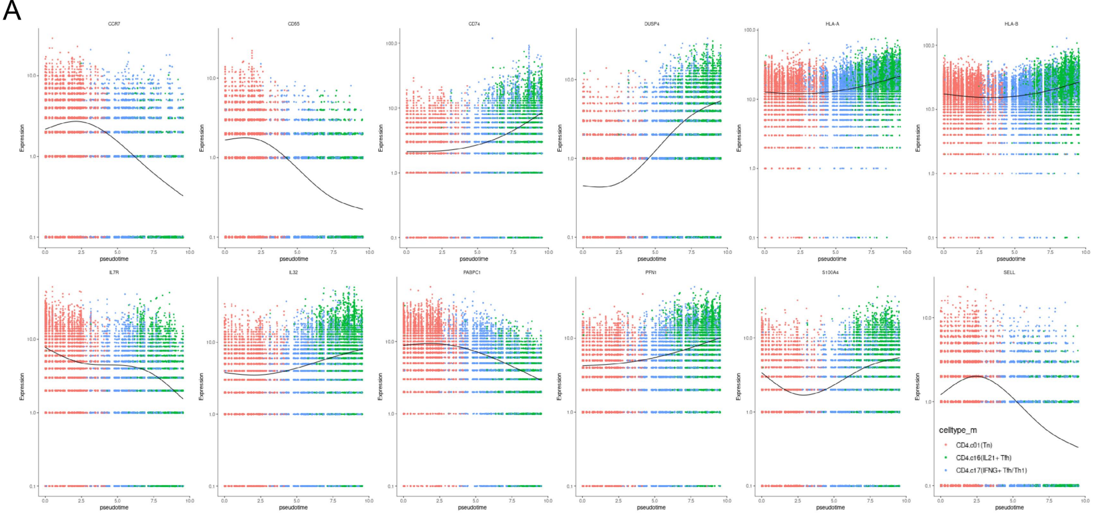

B

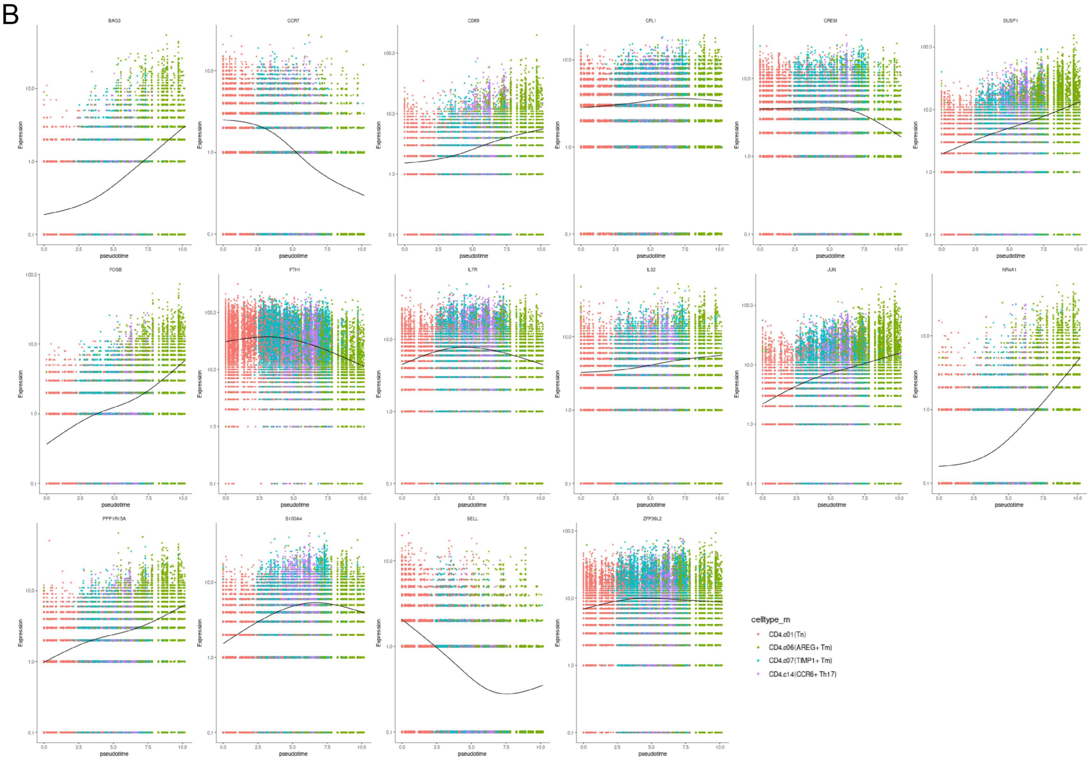

Supplementary Figure 3. Characterization of CD4$^+$ T cells along pseudotime.
A. Scatter plot of certain signature genes among CD4.c01/16/17 ordered along pseudo-time. Points are colored by cell clusters.
B. Scatter plot of certain signature genes among CD4.c01/06/07/14 ordered along pseudo-time. Points are colored by cell clusters.

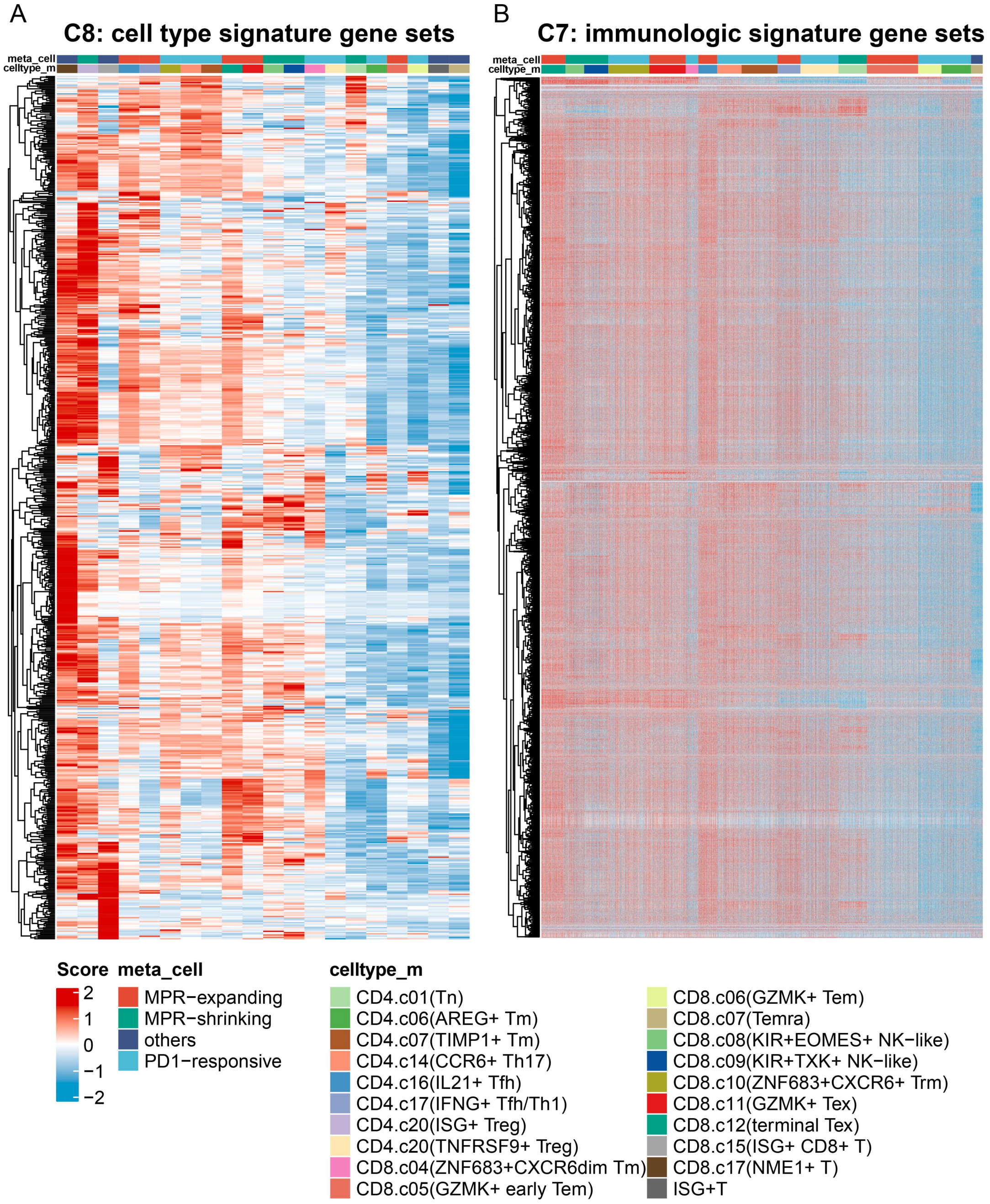

Supplementary Figure 4. Function annotation based on MSigDB.

A. Heat map of 830 MSigDB C8 gene sets scores in each cluster.

B. Heat map of 5,219 MSigDB C7 gene sets scores per cell in each cluster.

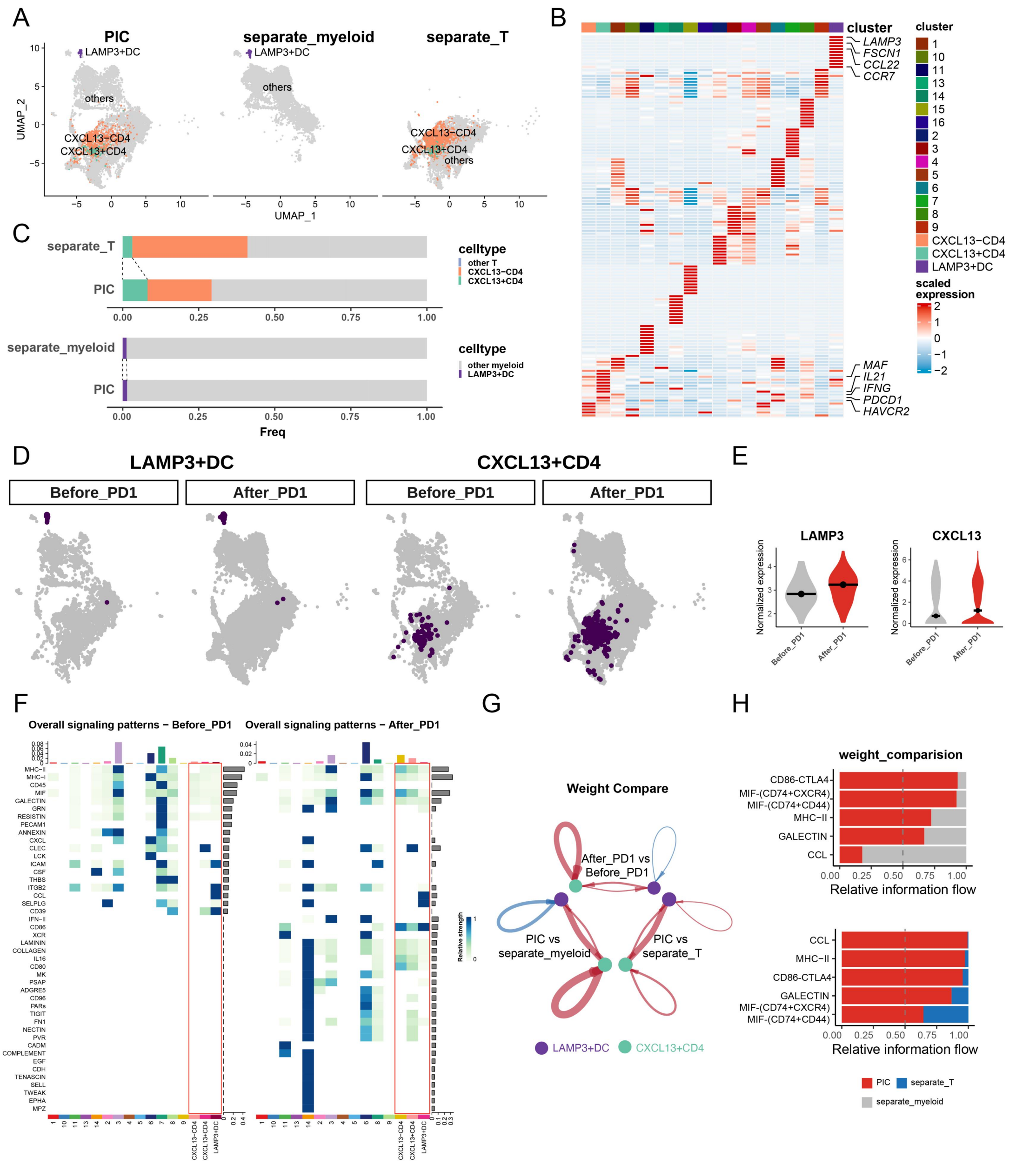

Supplementary Figure 5. Interaction between CXCL13^{+}CD4^{+} T cells and LAMP3^{+} DC strengthened post-ICIs revealed by PIC-seq.

A. UMAP of cell clusters from PIC-seq dataset developed by Zada et al, which are further defined by B; n = 31450 cells.

B. Heat map of DEGs found in clusters from PIC-seq.

C. Bar plots of cluster distribution among cells from PIC (physically interacting cells), separate_myeloid (single CD11c^{+} cells), separate_T (single CD3^{+} cells).

D. UMAP of LAMP3^{+}DC and CXCL13^{+}CD4^{+} T cells distribution before or after PD1.

E. Violin plot showing LAMP3 expression in LAMP3^{+}DC and CXCL13 in CXCL13^{+}CD4^{+} T cells before and after PD1.

F. The overall signaling patterns between CellChat curated pathways and defined cell clusters.

G. Differential interaction strength between LAMP3⁺DC and CXCL13⁺CD4⁺ T cells before versus after PD1, PIC versus separate_myeloid and PIC versus separate_T; red arches: up-regulation; blue arches: down-regulation.

H. Select signaling pathways with significant differences in the overall information flow of LAMP3⁺DC and CXCL13⁺CD4⁺ T cells between PIC versus separate_myeloid or separate_T.

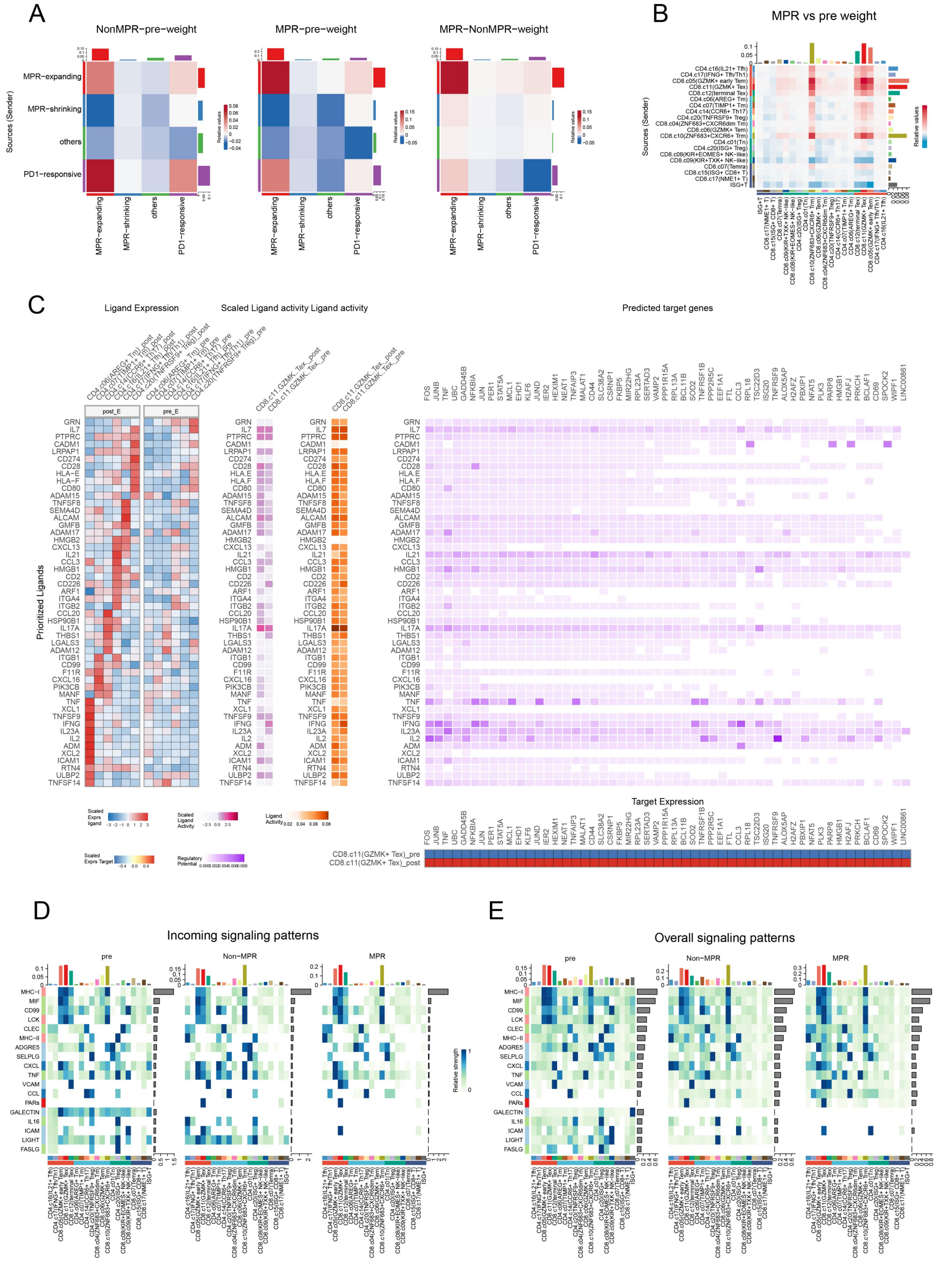

Supplementary Figure 6. Cell communication among MPR-E enhanced in MPR than Non.

A. Heat map of differential interaction strength between any pair of two meta-clusters.
B. Heat map of interaction strength comparisons of significant ligand-receptor pairs between any pair of two clusters; red meaning upregulated in MPR.
C. Ligands with top 50 prioritization scores (50-ligands) expression in CD4^{+} T cells in MPR-E and PD1-R meta-clusters post and pre (left). Outcome of NicheNet's ligand activity prediction and scaled ligand activity on DEGs upregulated in CD8.c11 within post-ICIs comparing to pre-ICIs (middle). Scaled expression of target genes in CD8.c11 (right lower) and NicheNet's ligand–target matrix denoting the regulatory potential between 50-ligands and target genes with meta-pathways annotation on the right (right upper).
D. The incoming (D) and overall (E) signaling patterns between CellChat curated pathways and defined cell clusters.

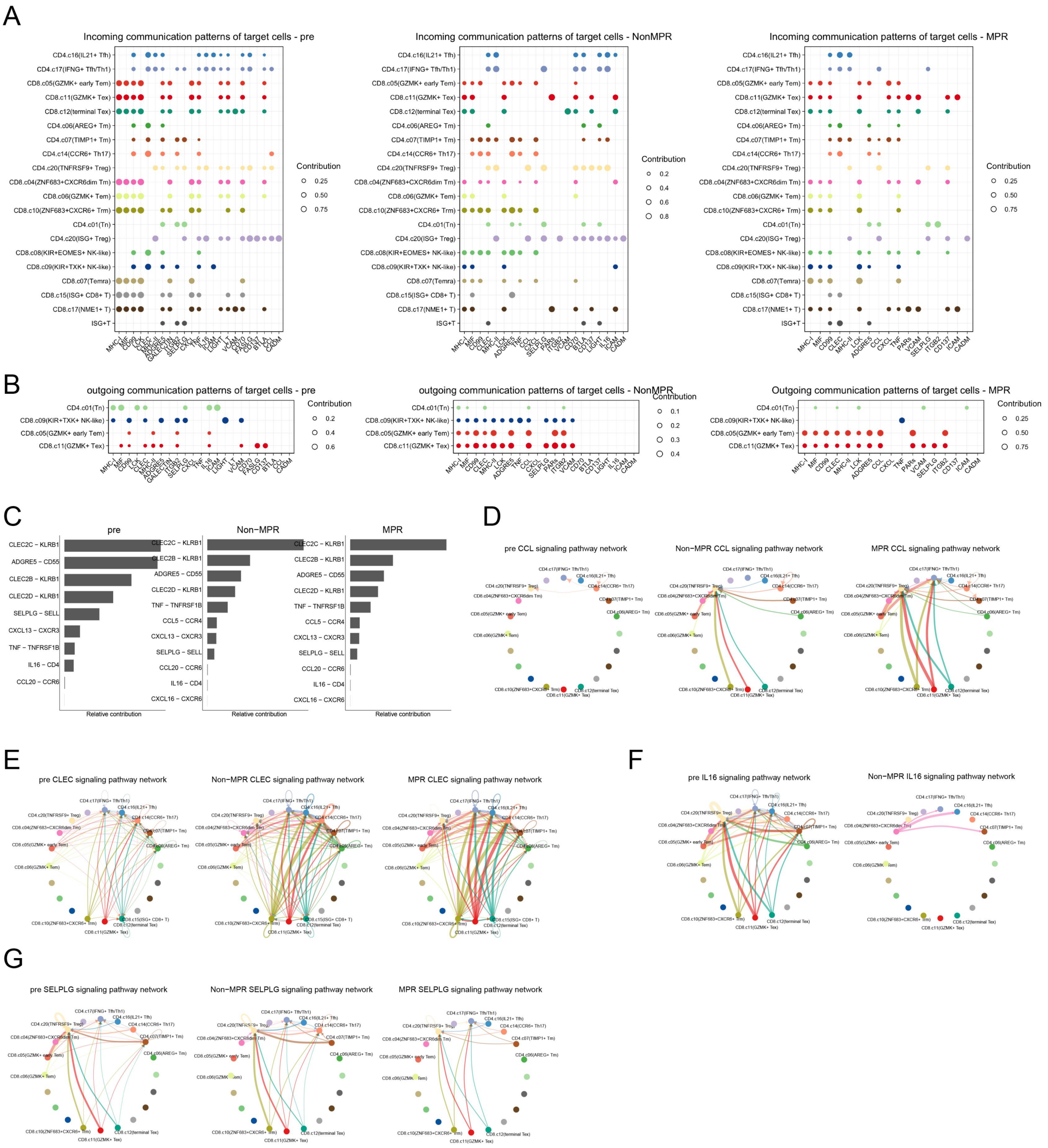

Supplementary Figure 7. Intercellular ligand–receptor prediction revealed by CellChat.

A-C. Relative contribution of each ligand-receptor pair at pre, Non and MPR.

D-G. The inferred CCL, CLEC, IL16, SELPLG signaling network among the cell populations represented by the nodes. Subclusters' color distribution as in Fig2G, edge width representing the pathway specific interaction strength.

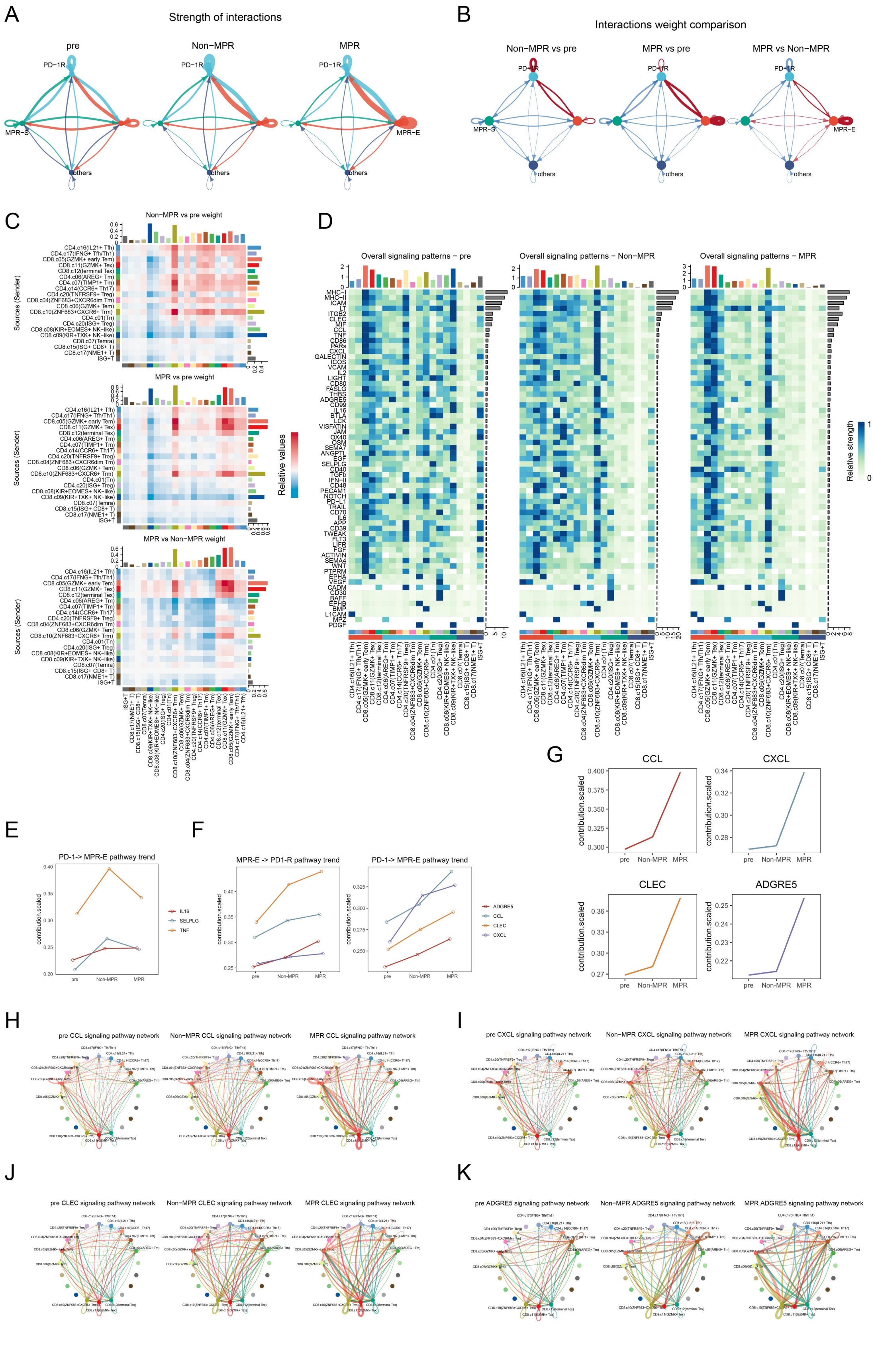

Supplementary Figure 8. Intercellular ligand–receptor prediction with VIPER.

A. Strength of significant ligand-receptor pairs between any pair of two meta-clusters in VIPER. The edge width is proportional to the indicated strength of ligand-receptor pairs.
B. Differential interaction strength between any pair of two meta-clusters in VIPER. Red means upregulated while blue means downregulated.
C. Heat map of interaction strength comparisons of significant ligand-receptor pairs between any pair of two clusters. Red means upregulated in Non (upper), MPR (middle and lower).
D. The overall signaling patterns between CellChat curated pathways and defined cell clusters in VIPER.
E. Scaled contribution of each pathway from PD1-R to MPR-E in pre, Non and MPR in VIPER.
F. Scaled contribution of each pathway between MPR-E and PD1-R in pre, Non and MPR in VIPER.
G. Scaled contribution of each pathway within MPR-E in pre, Non and MPR in VIPER.
H-K. The inferred CCL, CXCL, CLEC, ADGRE5 signaling network among the cell populations represented by the nodes from VIPER. Subclusters' color distribution as in Fig2G, edge width representing the pathway specific interaction strength.

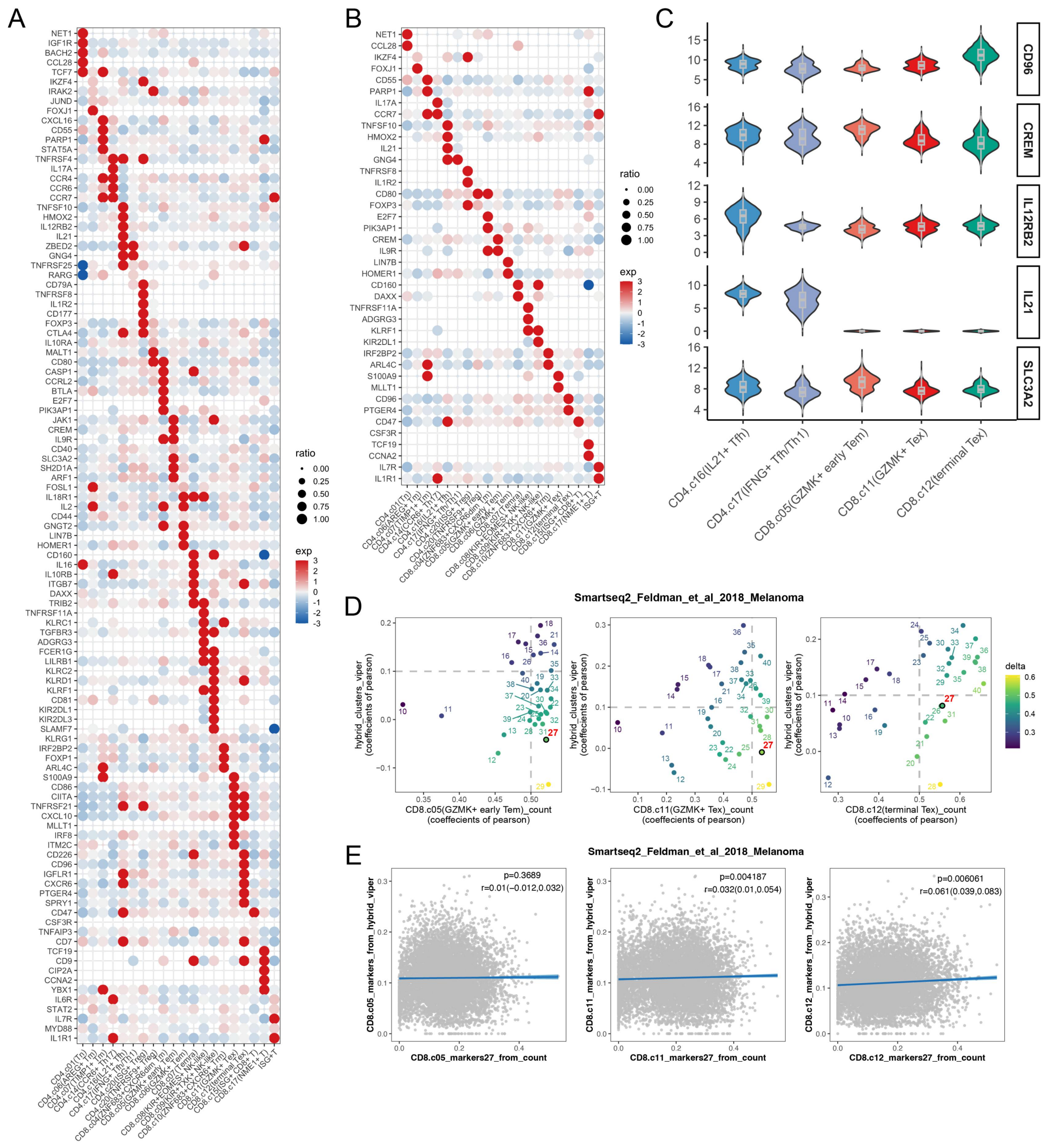

Supplementary Figure 9. Signature genes of cell clusters within VIPER.

A. Dot plot of differentially expressed genes found in each cluster from VIPER after scCODE-filter (method).

B. Dot plot of top20 differentially expressed genes found in each cluster from VIPER without scCODE-filter (method).

C. Violin plot of certain genes selected from DEGs of each cluster.

D. Number aside each dot representing the number of top-ranked genes picked from the feature genes of a given subgroup (called ‘n genes’ below) (CD8.c05 (left), CD8.c11 (middle), CD8.c12 (right)); x axis representing Pearson coefficient values between n genes from VIPER DEGs and n genes from original sc count DEGs; y axis representing Pearson coefficient values between n genes from VIPER DEGs and n genes from feature genes of all the other sub-clusters in MPR-E in VIPER (for example, CD4.c16, CD4.c17, CD8.c11, CD8.c12 (left)); color (delta) representing the value of x axis minus y axis (method).

E. Relationship of 27 markers of CD8.c05/11/12 from hybird_VIPER versus count; fit with a linear regression model. P values were determined by a two-sided linear regression t-test.

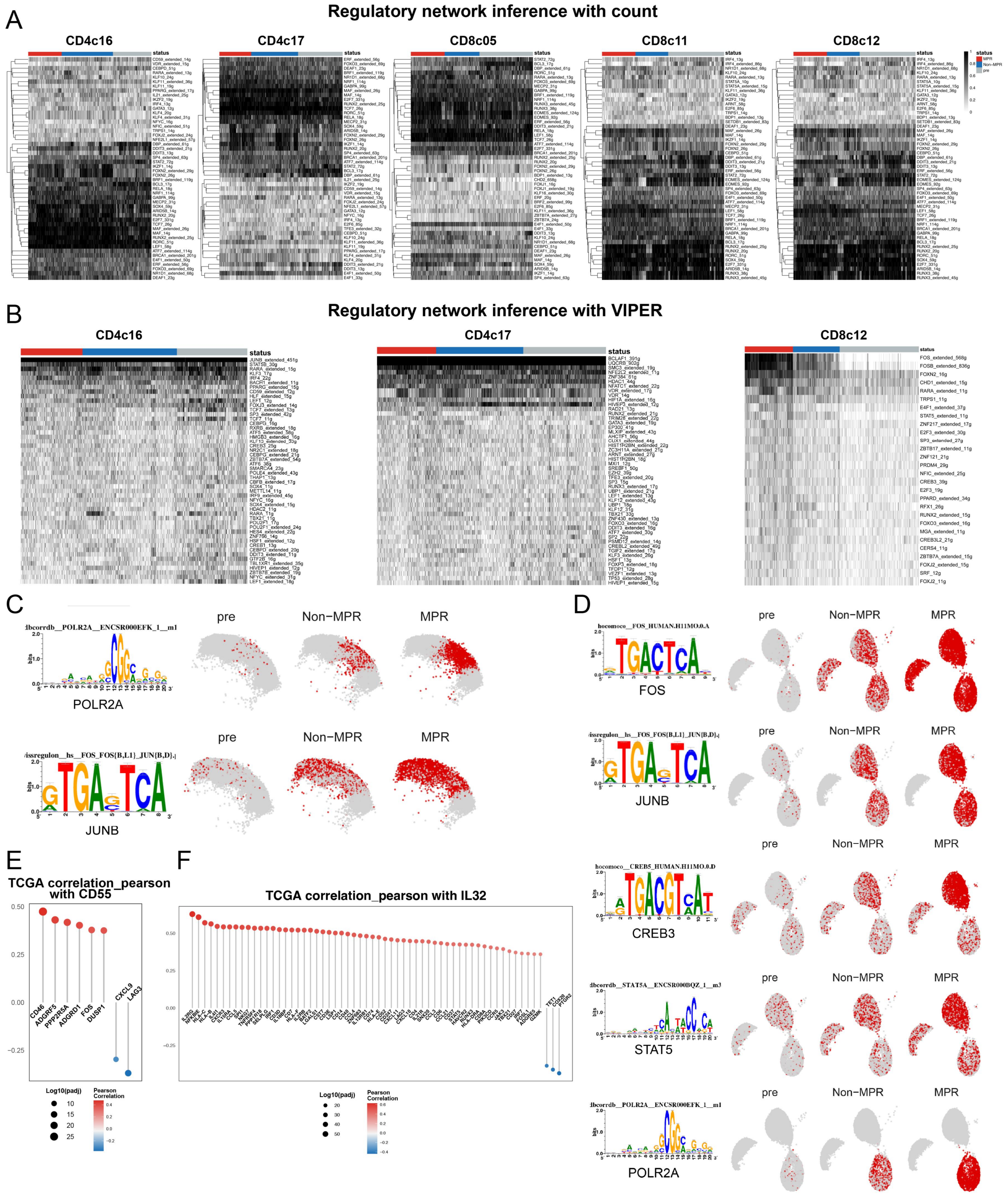

Supplementary Figure 10. Regulatory network along STAT5-IL32_ADGRE5 axis.

A. SCENIC of clusters in MPR-E on original sc count data, before supplements of VIPER-pro.

B. SCENIC results on CD4.c16/17 and CD8.c12 after supplements of VIPER-pro; TFs ranked by the expression levels in MPR.

C. Motifs of PLOR2A and JUNB and expression of them in CD8.c05/11/12 from pre, Non to MPR.

D. The motif of 3 irTFs, PLOR2A and JUNB and expression of them in CD8.c05/11/12 in pre, Non and MPR in VIPER. n = 36,412 cells.

E-F. Pearson correlation coefficients of CD55 (left) or IL32 (right) with all the other genes in pan-cancer TCGA database; ranked by coefficients value. P values were determined by a two-sided linear regression t-test.

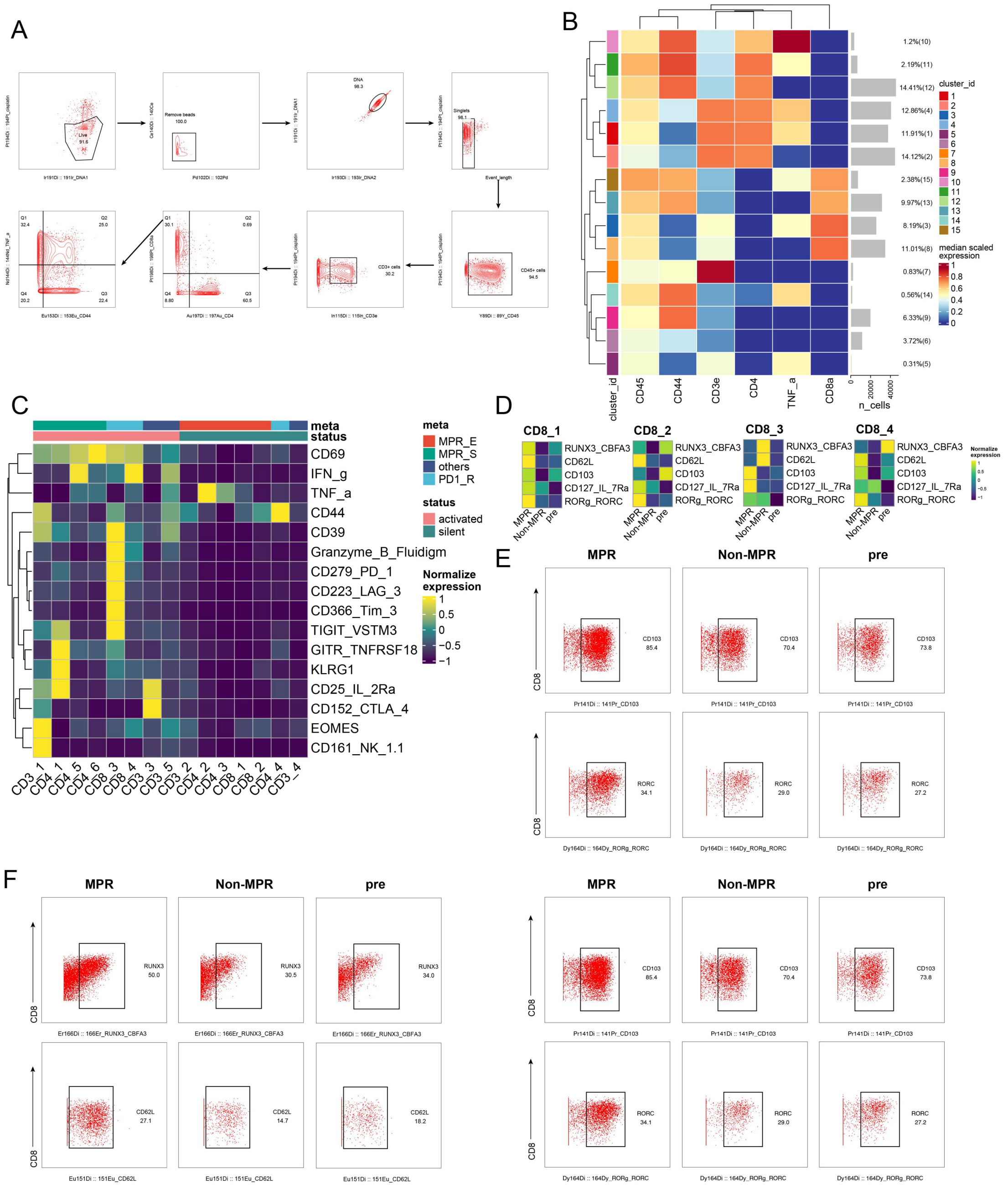

Supplementary Figure 11. Characterization of cell clusters from CYTOF.

A. Gating strategy from CD45^{+} TIL in CYTOF, markers listed in A all appearing in B.

B. Heat map showing NRS-based selection markers in each cluster. The right bar graph sums the cell numbers per cell type.
C. Heat map showing certain markers in each cluster.
D. Heat map showing CD103 (up) and RORC (down) in CD8_1 across MPR, Non and Pre.
E. Heat map showing RUNX3, CD62L. CD103, RORC (from above to below) in CD8_2 across MPR, Non and Pre.

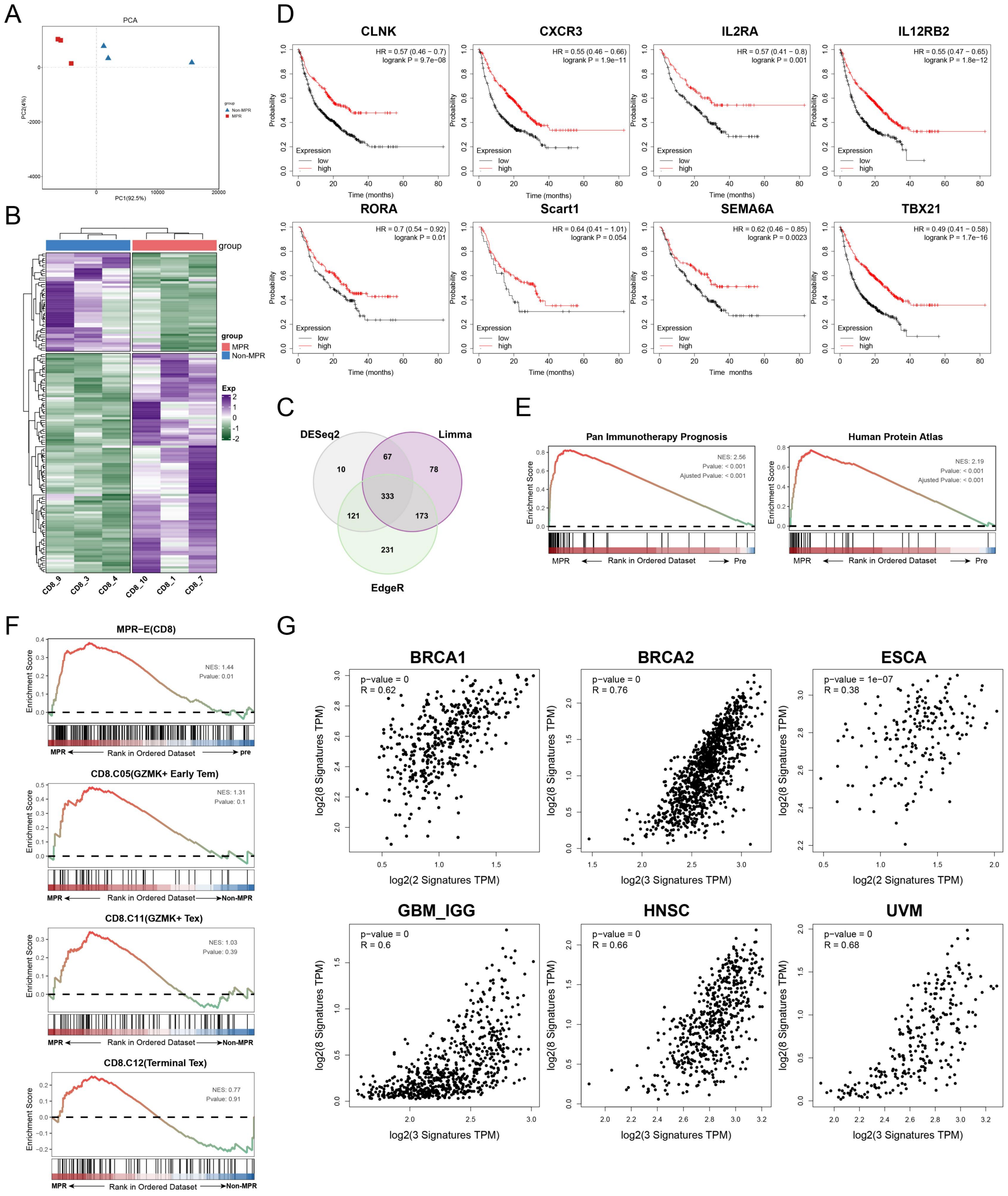

Supplementary Figure 12. survT sub-cluster in MPR-E mapped to murine TME.
A. PCA plot of murine CD8^{+} TIL of MPR and Non from bulk RNA-seq data.
B. Heat map of DEGs in MPR versus Non in murine bulk RNA-seq data.

C. Venn diagram of overlap of DEGs calculated by DEseq2, Limma and EdgeR, intersected genes undergoing down-stream analyses.
D. Kaplan-Meier plots showing each PPI genes indicating better OS in PIC. Hazard ratios (HR) was calculated using stratified Cox proportional hazards regression models, and p values were calculated using a stratified log rank test.
E. GSEA results for PIC and HPA signature (genes in Figure 4I) across MPR and Pre in murine CD8^{+} TIL.
F. GSEA results for MPR-E signature across MPR and Pre (upper) and CD8.c05/11/12 signature across MPR and Non in murine CD8^{+} TIL (signatures mentioned here from Figure 1G-based data).
G. Relationship of PPI signature and IL32-ADGRE5-CD55 axis in TCGA datasets (BRCA, ESCA, GBM_LGG (GBM integrated with LGG because of the same tumor site), HNSC, UVM). P values were determined by a two-sided linear regression t-test.

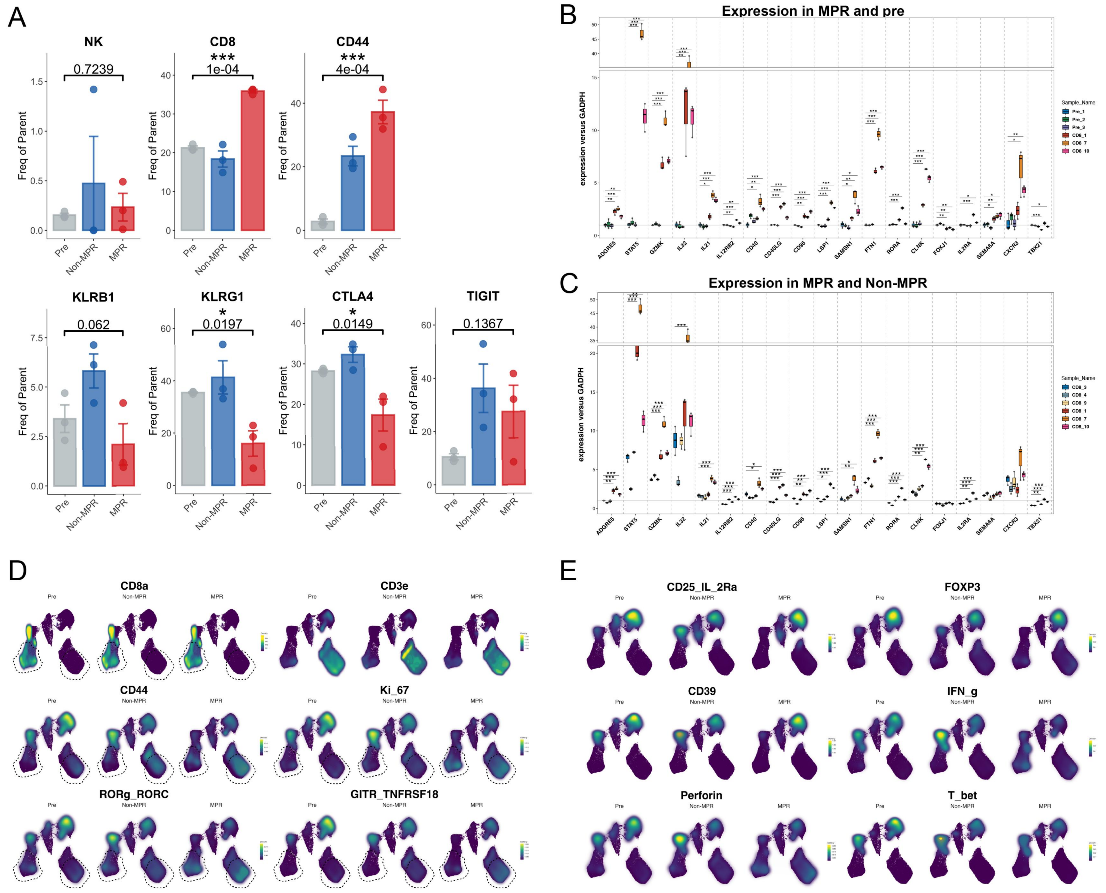

Supplementary Figure 13. Murine TME in pre, Non and MPR.
A. Flow cytometry analysis of the frequency (freq) of NK and CD8^{+} TIL and CD44, KLRB1, KLRG1, CTLA4, TIGIT expression in CD8^{+} TIL. Statistical testing between samples in MPR, Non and Pre by one-way ANOVA test (*$p < 0.05$, **$p < 0.01$, ***$p < 0.001$).
B-C. Expression of other upregulated genes in Fig4I normalized to Pre (B) or Non (C) by qRT-PCR. Statistical testing between samples in MPR and CD8_3 or Pre_1 by two-sided t test (*$p < 0.05$, **$p < 0.01$, ***$p < 0.001$).

D-E. UMAP plots showing the expression levels of certain signature genes in MPR, Non and Pre in CYTOF.

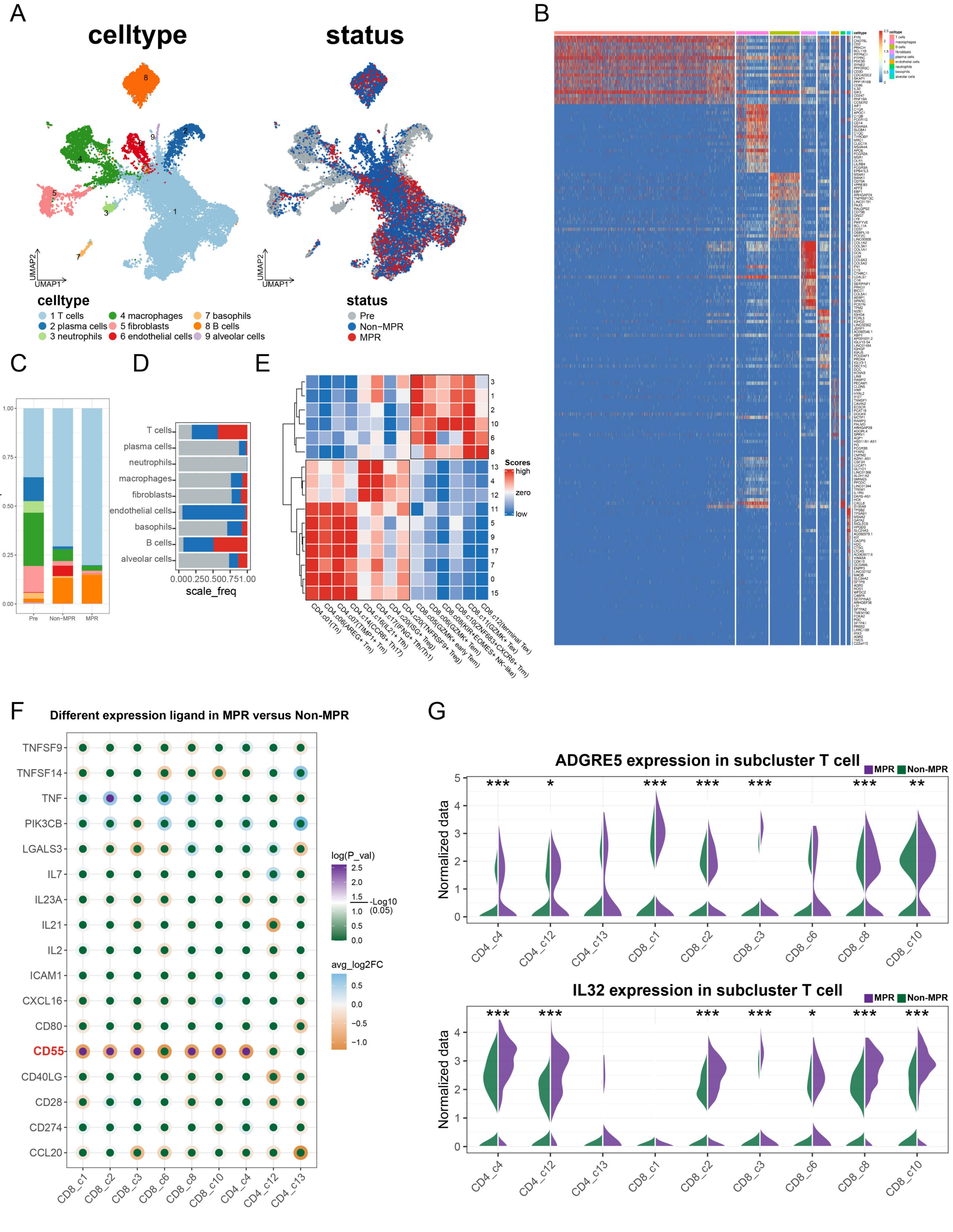

Supplementary Figure 14. Global analysis of scRNA-seq data on TIL in the independent NSCLC ICIs cohort.

A. UMAP of cell clusters and status from the independent NSCLC patients' cohorts (n = 23,601 cells).

B. Heat map showing DEGs of each cluster.

C. Bar plots of cluster distribution across MPR, Non or Pre.

D. Bar plots of status distribution in different cells clusters.
E. Heat map showing correlation scores calculated by SingleR between all clusters from Figure 5B with cell clusters in Figure 1C.
F. Comparison of expression of the 19 ligands from Figure 5C between MPR and Non in different cell clusters in Figure 5B.
G. Semi-violin-plot showing differentially expression of ADGRE5 (up) and IL32(down) between MPR and Non in clusters from Figure 5B. Test by two-sides t test (*p < 0.05, **p < 0.01, ***p < 0.001).

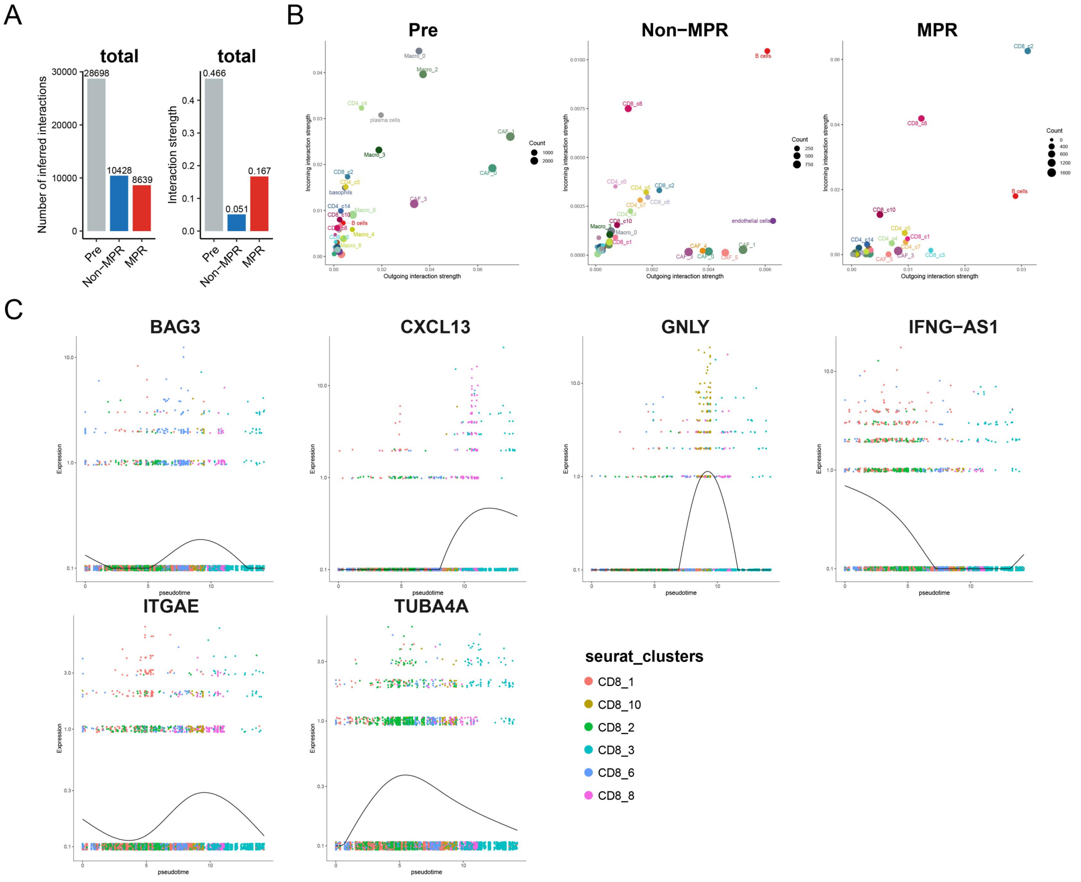

Supplementary Figure 15. Cell communication and trajectory analysis of CD8$^+$ T cells.

A. The total number (left) or strength (right) of CellChat-inferred interactions among each status (gray: pre, blue: Non, red: MPR).
B. Comparing the outgoing and incoming interaction strength in 2D space in each status. Each dot denotes an individual cluster colored by meta-clusters.
C. Scatter plot of certain signature genes among CD8$^+$ T cells in MPR-E ordered along pseudo-time. Points are colored by cell clusters.

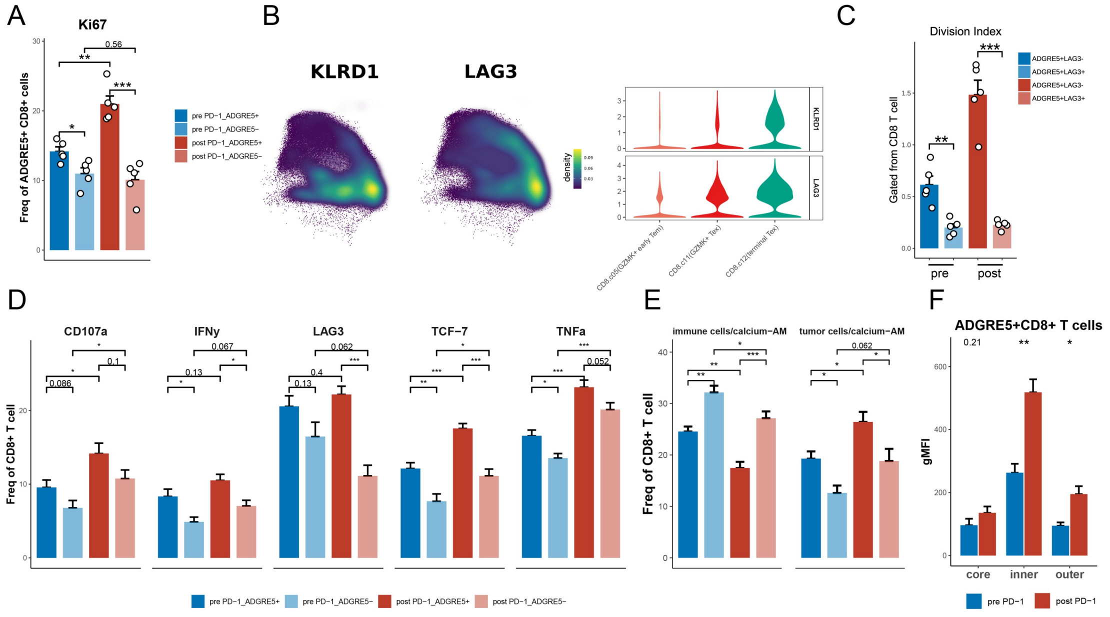

Supplementary Figure 16. ADGRE5^{+} CD8^{+} T cells repsonding to anti-PD-1.

A. Bar plot showing Ki67 expression on AGDRE5^{+} or ADGRE5^{-} CD8^{+} T cells pre- and post-PD1.

B. Umap plot showing KLRD1 and LAG3 expression on CD8^{+} cell clusters, as well as violin plot.

C. Flow cytometry analysis of CFSE results on ADGRE5^{+} or ADGRE5^{-} CD8^{+} T cells pre- and post PD1.

D. Flow cytometry analysis of specific markers expression on AGDRE5^{+} or ADGRE5^{-} CD8^{+} T cells pre- and post-PD1.

E. Flow cytometry analysis of survival ability of AGDRE5^{+} or ADGRE5^{-} CD8^{+} T cells pre- and post-PD1.

F. Bar plot showing MFI of AGDRE5^{+} CD8^{+} T cells in 3D HYGTIC system pre- and post-PD1.

Data in all quantitative panels, are displayed as the mean ± SEM. n = 5, *p < 0. 05, **p < 0.01, ***p < 0.001, two-tailed unpaired t test. T.

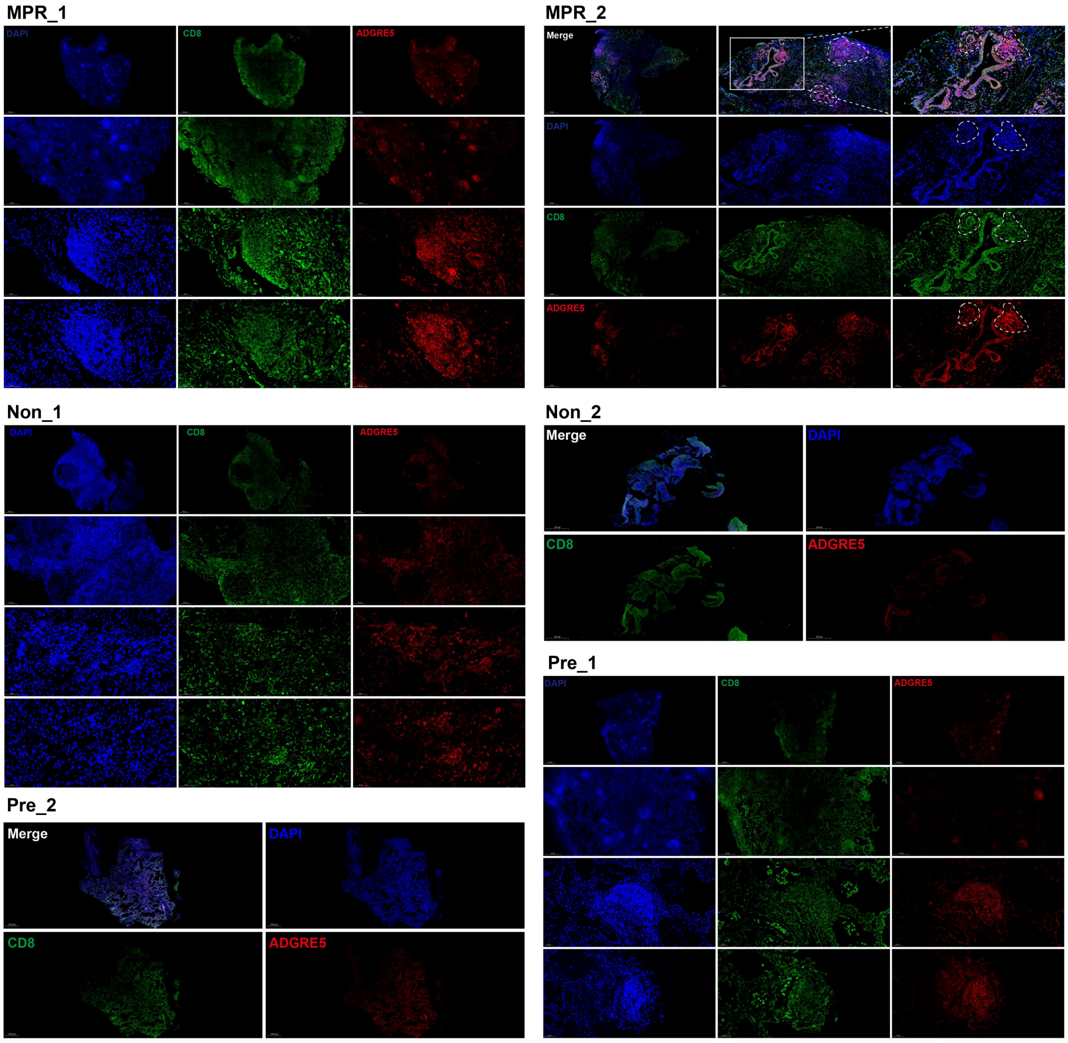

Supplementary Figure 17. Images of mIF in Pre, Non and MPR from NSCLC patients.

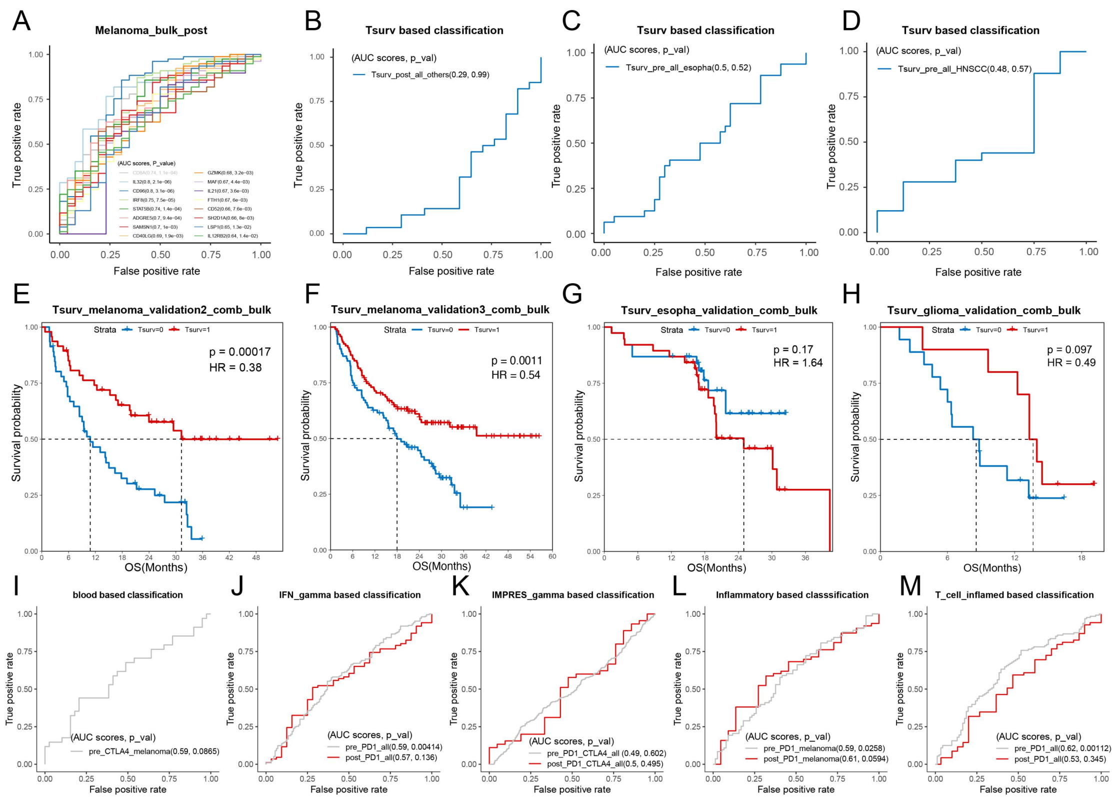

Supplementary Figure 18. Tsurv predicting prognosis to anti-PD1 therapy.

A. ROC curves for the performance of each gene in Tsurv in melanoma post bulk-RNA-seq data (training dataset). Data were summarized from n = 103 samples.

B-D. ROC curves for the performance of Tsurv in other cancer types post-ICIs (B) and ESCA (C) and HNSCC (D) pre-ICIs. Data were summarized from n = 45 samples from others; 72 samples from ESCA; 33 samples from HNSCC.

E-H. Kaplan-Meier plots for the performance of Tsurv in melanoma, ESCA and GBM with combined bulk-RNA-seq data. Data were summarized from n = 93 (validation2) and 211 (validation3) samples from melanoma; n = 76 samples from ESCA; n = 28 samples from glioma. Hazard ratios (HR) was calculated using stratified Cox proportional hazards regression models, and p values were calculated using a stratified log rank test.

I-M. ROC curves for the performance of other 5 published signature in pre- or post bulk-RNA-seq data. Data were summarized from n = 73 samples for blood; n = 337 and 118 samples for IFN_gamma from pre or post; n = 792 and 66 samples for IMPRES from pre or post; n = 160 and 85 samples for Inflammatory from pre or post; n = 219 and 99 samples for T_cell_inflamed from pre or post.

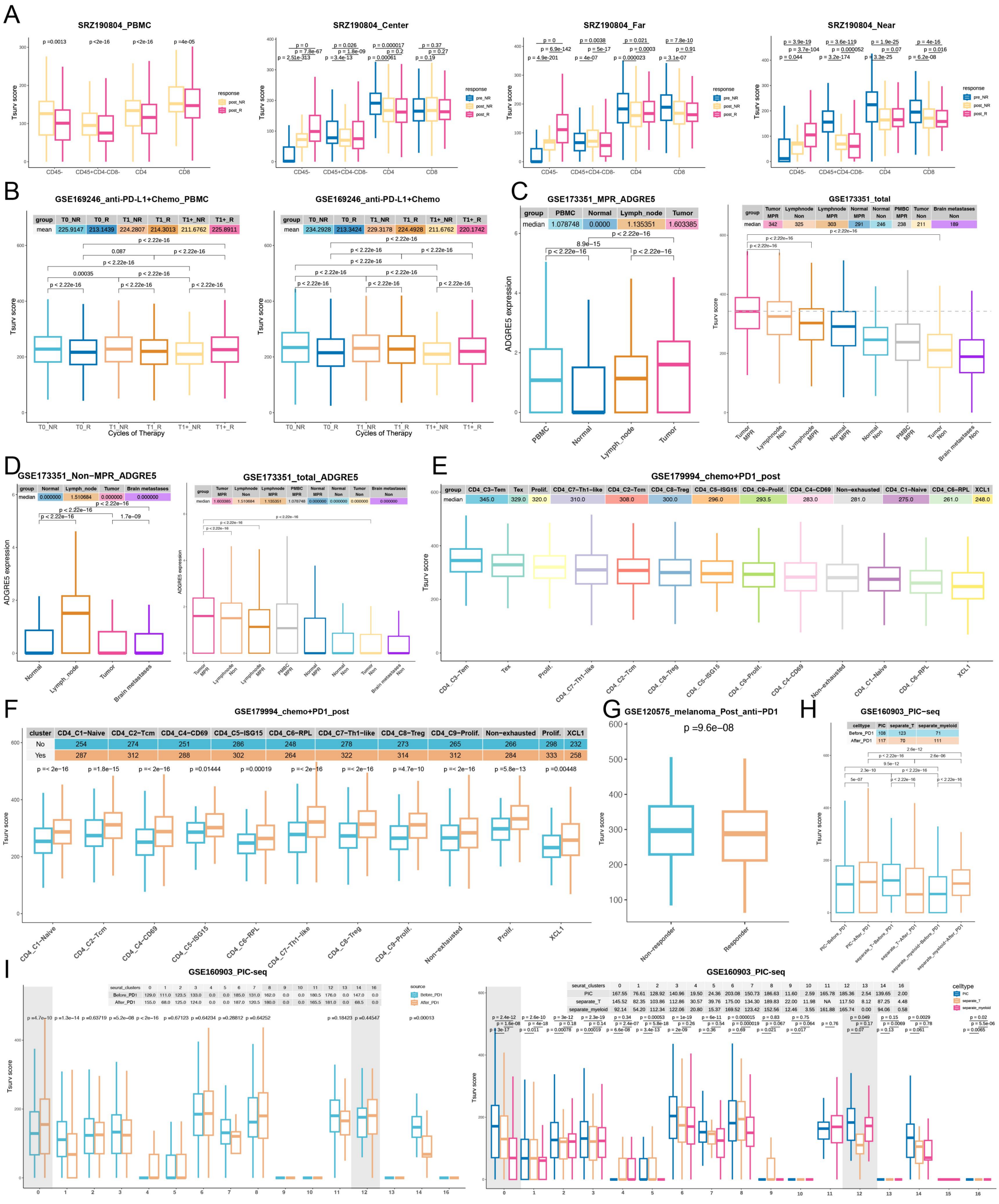

Supplementary Figure 19. Tsurv model classifying MPR/R from Non/NR.

A-I. Comparison of the Tsurv scores in cells from PIC. Each dot represents one cell while the center line indicates the median value. The lower and upper hinges represent the 25th and 75th percentiles, respectively, and whiskers denote 1.5× interquartile range. Two-sided t-test. Data were summarized from n = 167,683 cells from SRZ190804, n= 51,701 cells from GSE166181, n= 489,490 cells from GSE169246, n= 409,639 cells from GSE173351, n= 489,490 cells from GSE169246, n= 150,849 cells from GSE179994, n= 16,291 cells from GSE120575, n= 29,004 cells from GSE123813 and n= 31,450 cells from GSE160903.

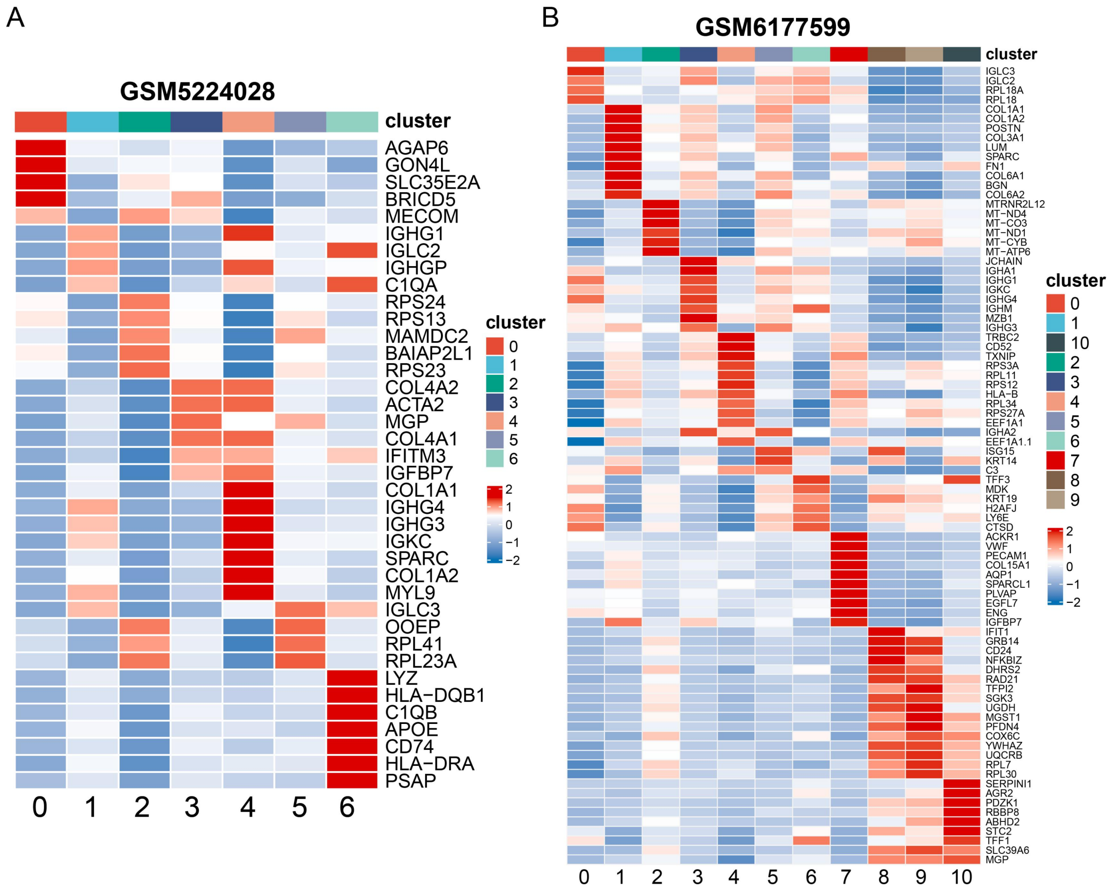

Supplementary Figure 20. Signature genes of cell clusters.

A. Heat map of DEGs found in each cluster from GSM5224028.

B. Heat map of DEGs found in each cluster from GSM6177599.

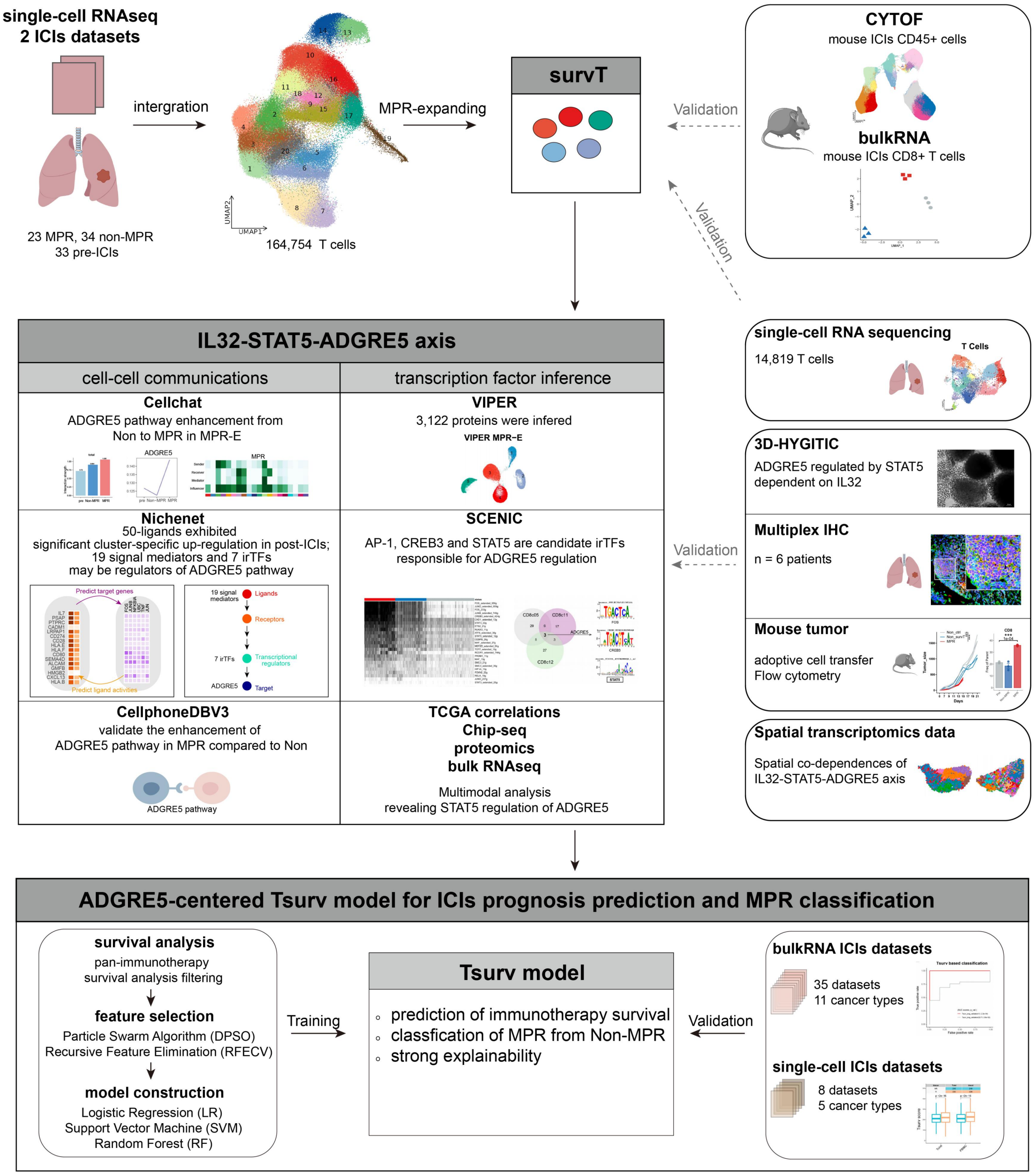

Supplementary Figure 21. Schematic outline of the overall concept used in this study.

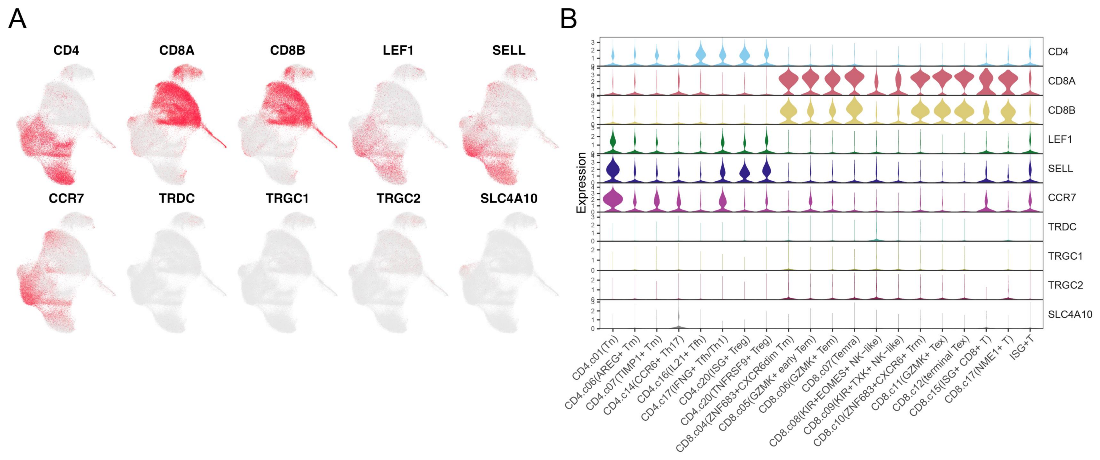

Supplementary Figure 22. Feature genes presentation.

A. UMAP feature-plots showing the expression levels of certain genes.

B. Vlnplot of differentially expressed genes found in each cluster.

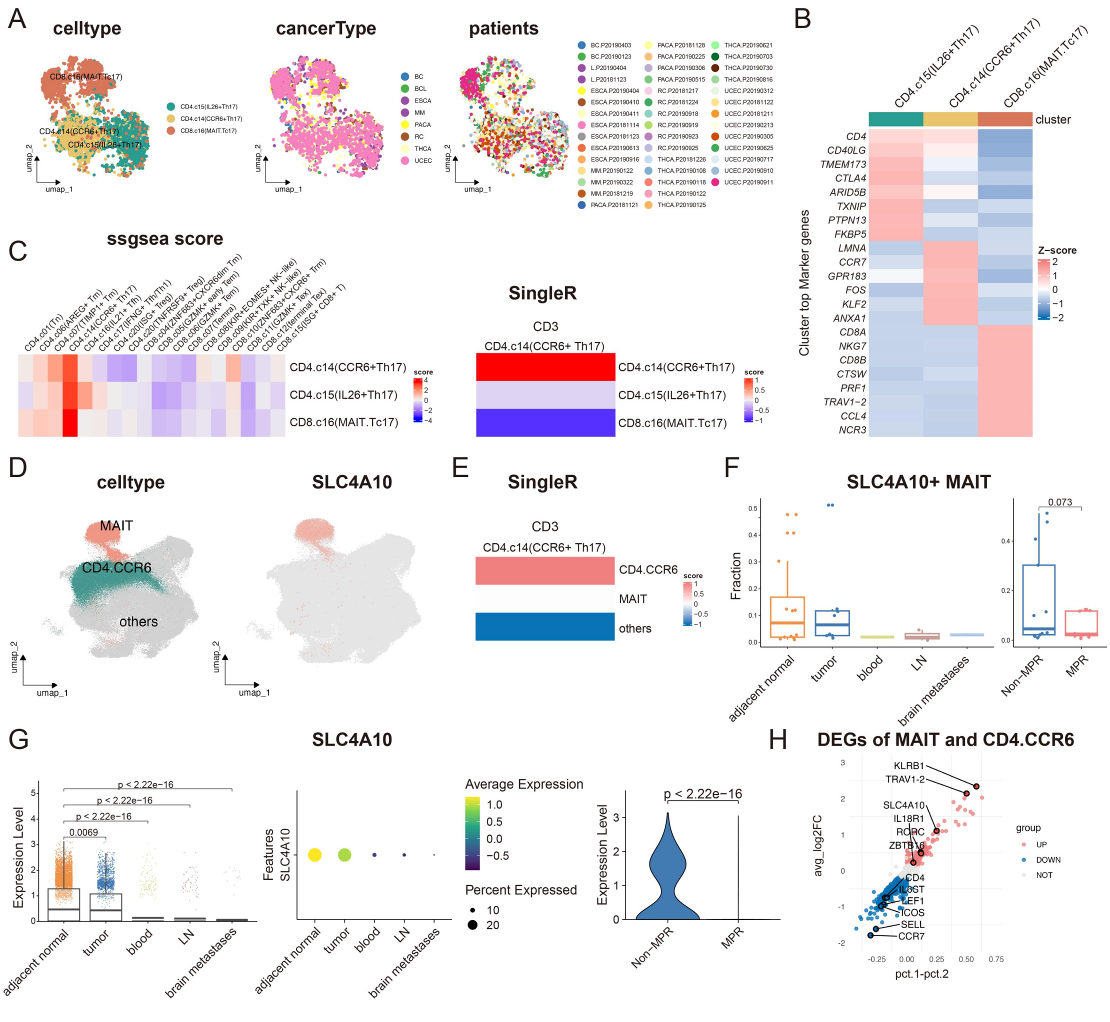

Supplementary Figure 23. Characterization of MAIT in pan-cancer.

A. UMAP plots showing 3 clusters of T cells, colored by clusters, cancerType and patients.
B. Heat map of DEGs found in each cluster.
C. Heat map of CD4.c14(CCR6+Th17), CD4.c15(IL26+Th17) and CD8.c16(MAIT.Tc17) gene sets scores in each cluster in CD3 dataset (left) and CD4.c14(CCR6+Th17) scores in this dataset.
D. UMAP plots showing MAIT and CD4.CCR6 cluster and the expression levels of SLC4A10 in another dataset.
E. Heat map of CD4.c14(CCR6+Th17) scores in this dataset calculated by SingleR.
F. Comparison of the frequency of SLC4A10+ MAIT respectively in different lesions and Non-MPR and MPR patients. Statistical testing was calculated by two-sided t-test.
G. The expression of SLC4A10 of MAIT cluster from different lesions and Non-MPR and MPR patients.
H. Volcano plot showing differentially expressed genes between MAIT and CD4.CCR6 clusters; each colored dot denoting an individual gene with adjusted P value < 0.05 and |log (Fold change) | ≥ 0.15.

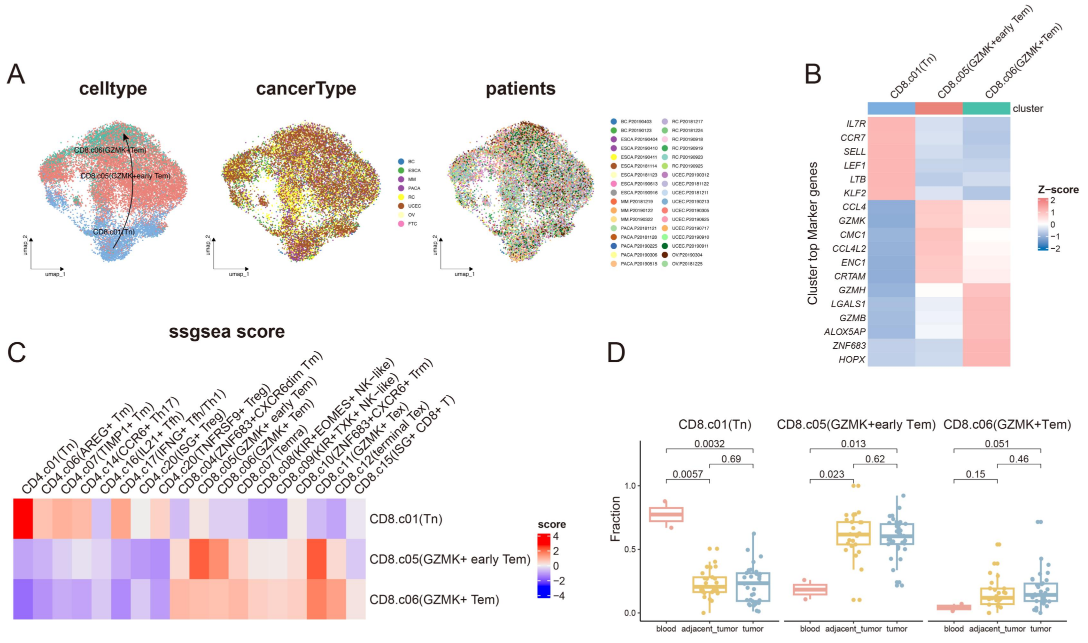

Supplementary Figure 24. Characterization of naïve CD8+ T cells in pan-cancer.

A. UMAP plots showing 3 clusters of T cells, colored by clusters, cancerType and patients.
B. Heat map of DEGs found in each cluster.
C. Heat map of CD8.c01(Tn), CD8.c05(GZMK+ early Tem) and CD8.c06(GZMK+ Tem) gene sets scores in each cluster in CD3 dataset.
D. Comparison of the frequency of each cluster respectively in different lesions. Statistical testing was calculated by Wilcox-test.

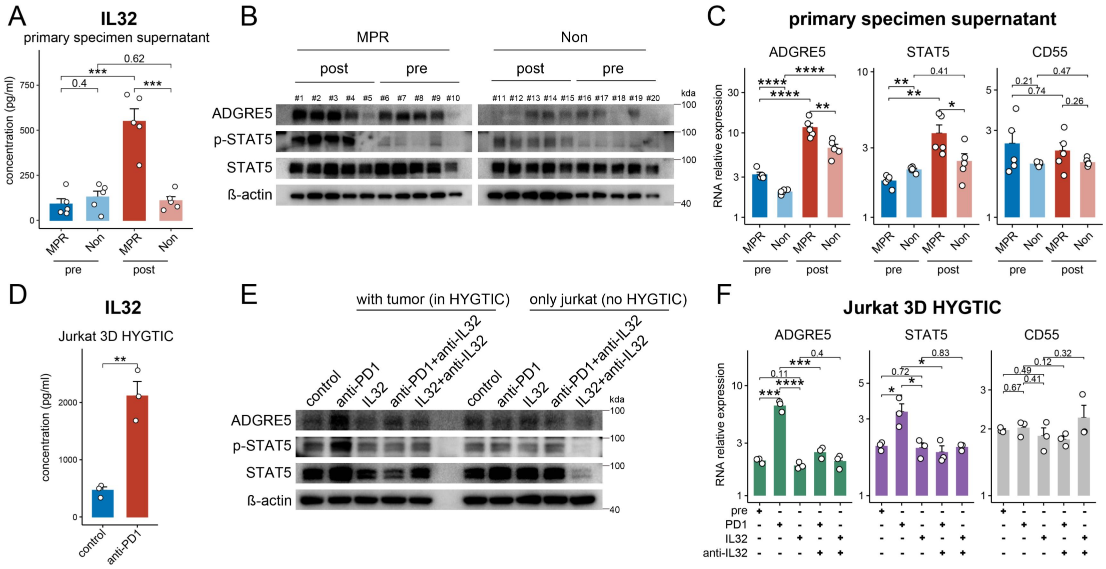

Supplementary Figure 24. **ADGRE5 regulated by STAT5 dependent on IL32.**

A. The concentration of IL32 detected from primary specimen supernatant from MPR and Non patients pre- and post-PD1.
B. The expression of ADGRE5, Phospho-STAT5 (Tyr694) and STAT5 in primary specimen from MPR and Non patients pre- and post-PD1 examined by immunoblotting.
C. The expression of ADGRE5, STAT5 and CD55 in primary specimen from MPR and Non patients pre- and post-PD1 examined by qPCR.
D. The concentration of IL32 from Jurkat T cells in 3D HYGTIC pre- and post-PD1.
E. The expression of ADGRE5, Phospho-STAT5 (Tyr694) and STAT5 of Jurkat T cells with or without tumor under the anti-PD1, IL32 or anti-IL32, examined by immunoblotting.
F. The expression of ADGRE5, STAT5 and CD55 of Jurkat T cells under the anti-PD1, IL32 or anti-IL32, examined by qPCR.
